# Base-promoted isomerization of CF_3_-containing allylic alcohols to the corresponding saturated ketones under metal-free conditions

**DOI:** 10.3762/bjoc.13.149

**Published:** 2017-08-01

**Authors:** Yoko Hamada, Tomoko Kawasaki-Takasuka, Takashi Yamazaki

**Affiliations:** 1 Division of Applied Chemistry, Institute of Engineering, Tokyo University of Agriculture and Technology, 2-24-16 Nakamachi, Koganei 184-8588, Japan

**Keywords:** allylic alcohols, chirality, computation, 1,3-proton shift, trifluoromethyl

## Abstract

Following to the computational expectation, our previously reported intriguing 1,3-proton shift of 4,4,4-trifluorobut-2-yn-1-ols was successfully extended to the 4,4,4-trifluorobut-2-en-1-ol system under metal-free conditions to afford the corresponding saturated ketones in high to excellent chemical yields using such a convenient and easy-to-handle base as DBU at the toluene refluxing temperature, and utilization of the corresponding optically active substrates unambiguously demonstrated that this transformation proceeded in a highly stereoselective fashion.

## Introduction

**Scheme 1:**
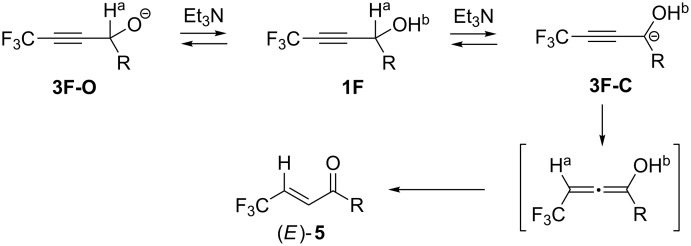
Et_3_N-promoted isomerization of propargylic alcohols **1F**.

We have previously reported [1–3] an extremely efficient isomerization process of γ-trifluoromethylated propargylic alcohols **1F** to the corresponding α,β-unsaturated ketones (*E*)-**5** by the action of a weak base like Et_3_N under THF reflux conditions (Scheme 1). From the mechanistic point of view, deprotonation of the propargylic proton H^a^ was considered to be the initial step of this reaction and this step would be in competition with the proton abstraction from the OH^b^ group in **1F**. Thus, it was our first understanding that during the equilibration between **3F-O** and **3F-C**, the irreversible reprotonation by way of the latter intermediate would be occurred at the CF_3_-attached carbon atom to produce the corresponding allenyl type intermediate. Furthermore, its keto–enol interconversion would result in construction of the isomerized α,β-unsaturated ketones (*E*)-**5** [4–6]. For a better comprehension of this interesting and efficient protocol, computation was performed [7] for obtaining the rough indication of the acidity of both protons H^a^ and H^b^ in **1F**. Moreover, the corresponding allylic alcohols **2F** as well as their non-fluorinated counterparts **1H** and **2H** were also employed for comparison whose results were summarized in Table 1.

**Table 1:** Destabilization energy Δ*E* of propargylic (**1**) and allylic (**2**) alcohols.

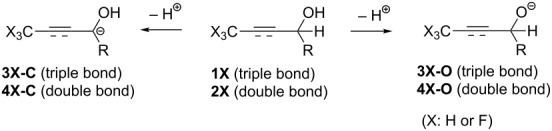					

C–C bond	X	R	Δ*E* (kcal/mol)		ΔΔ*E*^c^
	
			Δ*E*_C_^a^	Δ*E*_O_^b^	(kcal/mol)

triple	F	CH_3_ (**a**)	310.657	311.705	–1.048
triple		Ph (**b**)	301.759	309.628	–7.869
double		CH_3_ (**a**)	–^d^	316.344	–^d^
double		Ph (**b**)	313.332	313.653	–0.321

triple	H	CH_3_ (**a**)	338.596	319.647	18.949
triple		Ph (**b**)	324.957	317.154	7.803
double		CH_3_ (**a**)	351.919	324.077	27.842
double		Ph (**b**)	332.565	320.943	11.622

^a^Δ*E*_C_: *E*_3X-C_ − *E*_1X_ or *E*_4X-C_ − *E*_2X_. ^b^Δ*E*_O_: *E*_3X-O_ − *E*_1X_ or *E*_4X-O_ − *E*_2X_. ^c^ΔΔ*E*: Δ*E*_C_ − Δ*E*_O_. ^d^The stable **4F-Ca** was failed to be located using the IEFPCM method.

For this purpose, evaluation was performed on the basis of the energetic difference between neutral compounds and the corresponding carbanion or alkoxide species: thus, in the former instance, Δ*E*_C_ as *E*_3X-C_ − *E*_1X_ (or *E*_4X-C_ − *E*_2X_) or the latter Δ*E*_O_ as *E*_3X-O_ − *E*_1X_ (or *E*_4X-O_ − *E*_2X_) in the case of propargylic (or allylic) alcohols, respectively, as the simple measure of destabilization. In the case of the non-fluorinated **1H** and **2H** series, as expected, a clear alkoxide preference was noticed irrespective of the carbon–carbon unsaturation pattern. For the corresponding fluorinated counterparts, in spite of the failure to locate the energy minimum for **4F-Ca**, it was found out in other cases that Δ*E*_C_ values were unanimously smaller than Δ*E*_O_ which led to the definitive conclusion that protons at the propargylic and allylic positions should be more acidic than the ones at the corresponding OH groups. The strongly electron-withdrawing property of a CF_3_ group is considered to play a key role in stabilization of both **3F-C** and **4F-C**, and the phenyl group as well as the carbon–carbon multiple bonds would also provide the additional preferable effect by their efficient resonance. In the case of the propargylic alcohol **1Fa** with a methyl moiety as R, its electron-donating ability unambiguously destabilized the carbanionic species **3F-Ca** while the electron-withdrawal of a CF_3_ group nicely compensated this disadvantage, resulting in a better stability of **3F-Ca** by 1 kcal/mol with respect to the alkoxide **3F-Oa**. In the case of **1Fb**, the phenyl group worked nicely for increase of the energetic preference of **3F-Cb** to **3F-Ob** of about 7.9 kcal/mol. For the allylic alcohol **2Fb** (R = Ph), although the ΔΔ*E* value was small, the carbanionic species **4F-Cb** was calculated to be more (or at least almost equally) preferable to **4F-Ob**. This result allowed us to similarly anticipate the successful isomerization of CF_3_-containing allylic alcohols **2F** to the corresponding saturated ketones **7** when the substrates possessed appropriate aromatic substituents as R.

With reference to these computational results, we decided to employ the CF_3_-containing allylic alcohols (*E*)-**6** at the γ-position as substrates for the amine-promoted isomerization, instead of the corresponding propargylic alcohols **1F**. Although a similar Ru- [8–12] or Fe-catalyzed processes [13] have been previously demonstrated, our present work is considered to draw a clear line with these instances because of the apparently convenient metal-free process. Moreover, the same type of proton shift has also reported by two groups. For example, during the reaction of (*E*)-2-(trifluoromethyl)vinylsilane and benzaldehyde in the presence of an excess amount of CsF, the resultant product (*E*)-**2Fb** was further converted in situ to the corresponding saturated ketone (*E*)-**5b** (R = Ph). Confirmation of this process was performed by the action of DBU, resulting in 95% conversion of the starting allylic alcohol (*E*)-**2Fb** [14]. Quite recently [15], the same proton shift was published by using the bicyclic guanidine-type base, 1,5,7-triazabicyclo[4.4.0]dec-5-ene (TBD), as a catalyst. In spite of these two preceding studies, we have also succeeded in attaining a similar level of chemical yields as well as stereoselectivity to the latter process by using far-less expensive DBU (1,8-diazabicyclo[5.4.0]undec-7-ene), whose results are reported in this article in detail.

## Results and Discussion

Preparation of substrates (*E*)-**6** was carried out by way of our recently reported one-pot procedure [16]: thus, after reaction of an appropriate Grignard reagent and ethyl trifluoroacetate at −80 °C, a Horner–Wadsworth–Emmons reagent [17] activated by LiBr and Et_3_N [18–20] was introduced to this solution at room temperature, leading to the formation of (*E*)-**6** after the NaBH_4_ reduction of the resultant products (see Supporting Information File 1 for the detailed procedure).

As shown in Table 2, we at first checked a type of bases suitable for this isomerization in THF under reflux for 3 h. Although Et_3_N was appropriate in the case of isomerization of propargylic alcohols **1F** [1], this was not the case for the allylic alcohol (*E*)-**6a**, forming the desired **7a** only in a trace amount with recovery of (*E*)-**6a** in a large amount (Table 2, entry 1). Because Et_3_N was proved to be inadequate, utilization of DBU with the higher basicity was tried at the next stage although this base gave detrimental results for the isomerization of **1F**. Actually, DBU was found to work nicely in the present instance to afford the desired compound **7a** in 67% isolated yield (Table 2, entry 2). The result that the stronger tertiary amine DBU worked more effectively than Et_3_N was a clear support of our computation at least from a qualitative point of view which estimated the lower acidity of the allylic proton rather than the one in the propargylic position (ΔΔ*E* of −0.321 and −7.869 kcal/mol for the allylic and propargylic series, respectively). Due to the unacceptable efficiency of K_2_CO_3_ and NaOH (Table 2, entries 3–5), the solvent effect was surveyed at the next stage with fixing the base to DBU. Polarity seemed to affect the reaction significantly, and aprotic MeCN, DMF, and DMSO (Table 2, entries 9, 10, and 12, respectively) showed a similar potency to THF with respect to chemical yields of **7a**. Further investigation clarified that toluene was the best solvent from the standpoint of the material balance, and with taking efficiency into consideration, we eventually decided that, as described in Table 2, entry 18, 3 h reflux in toluene with 0.5 equiv of DBU were the conditions of choice.

**Table 2:** Optimization of reaction conditions.

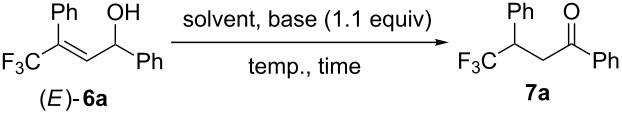						

					Yield^a^ (%)	
Entry	Solvent	Base	Temp. (°C)	Time (h)	**7a**	(*E*)-**6a**

1	THF	Et_3_N	reflux	3	3	[93]
2	THF	DBU	reflux	3	(67)	[20]
3	THF	K_2_CO_3_	reflux	3	–	[99]
4	THF	NaOH^b^	reflux	3	–	[99]
5	THF	NaOH^c^	reflux	3	(45)	[4]
6	MeOH	NaOH^b^	reflux	3	(49)	[–]
7	MeOH	DBU	reflux	3	4	[84]
8	MTBE	DBU	reflux	3	9	[95]
9	MeCN	DBU	70	3	(61)	[1]
10	DMF	DBU	70	3	64	[29]
11	DMA	DBU	70	3	54	[11]
12	DMSO	DBU	70	3	67	[15]
13	DCM	DBU	reflux	3	–	[94]
14	toluene	DBU	70	3	(46)	[44]
15	THF	DBU	reflux	24	(87)	[0]
16	toluene	DBU	70	24	(93)	[0]
17	toluene	DBU	reflux	3	(91)	[0]
18^d^	toluene	DBU	reflux	3	(91)	[0]
19^e^	toluene	DBU	reflux	24	(95)	[0]
20^d^	toluene	DABCO	reflux	10	30	[78]

^a^ All yields were determined by ^19^F NMR and in brackets were described the recovery of (*E*)**-6a**. In the parentheses were shown the isolated yields. ^b^A 6 M aqueous solution was used. ^c^Solid NaOH was used. ^d^0.5 equiv of base was used. ^e^0.1 equiv of base was used.

Because we have successfully determined the appropriate reaction conditions for the present intriguing isomerization, clarification of its scope and limitation was carried out whose results were summarized in Table 3. Entry 1 depicts the result already shown in entry 18 of Table 2 where the substrate (*E*)-**6a** was transformed into the saturated ketone **7a** in 91% yield. A slower reaction was suspected for (*E*)-**6b** in Table 3, entry 2 because of the possible destabilizing effect of the partial anionic charge at the 3 position by the electron-donating 4-MeOC_6_H_4_ group as R^2^. However, the effect was only quite limited and isomerization of (*E*)-**6b** was occurred in the same 3 h period as entry 1 with recording 89% isolated yield. Moreover, as expected, the electron-withdrawing 4-fluorophenyl moiety worked nicely to quantitatively furnish the ketone **7c** in shorter time (Table 3, entry 3). However, as shown in Table 3, entries 4 and 5, clear retardation of this process was noticed for substrates with alkyl groups as R^2^. Different from the case of R^2^, an electronic effect of the substituent on the benzene ring of R^1^ affected this transformation quite significantly, and the substrate (*E*)-**6f** with 4-MeOC_6_H_4_ and Ph(CH_2_)_2_- groups as R^1^ and R^2^, respectively, experienced “double retardation” to require 48 h for attainment of an adequate level of conversion. Because of such substituent sensitivity of R^1^, alkyl groups at this position completely inhibited the reaction (Table 3, entries 8 and 9). Entry 10 described the result for the non-fluorinated substrate (*E*)-**6j** possessing a CH_3_ group instead of a CF_3_ moiety, and its simple recovery was observed. Exchange of the strongly electron-withdrawing CF_3_ group to CH_3_ led to complete loss of the good stabilizing factor of the developing carbanionic species. As a result, the alkoxide was more energetically preferred and thus this proton shift became difficult.

**Table 3:** Transformation of allylic alcohols (*E*)-**6** to the corresponding ketones **7**.

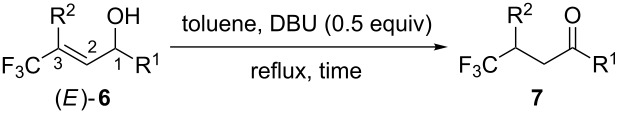					

				Yield^a^ (%)	
Entry	R^1^	R^2^	Time (h)	**7**	(*E*)-**6**

1	Ph	Ph	3	91 (**a**)	[–]
2	Ph	4-MeOC_6_H_4_-	3	89 (**b**)	[–]
3	Ph	4-FC_6_H_4_-	2	>99 (**c**)	[–]
4	Ph	Et	24	78 (**d**)	[3^b^]
5	Ph	Ph(CH_2_)_2_-	24	93 (**e**)	[–]
6	4-MeOC_6_H_4_-	Ph(CH_2_)_2_-	48	76 (**f**)	[–]
7	4-BrC_6_H_4_-	Ph(CH_2_)_2_-	3	91 (**g**)	[–]
8	Ph(CH_2_)_2_-	Ph	24	– (**h**)	[75]
9	Et	Ph	24	– (**i**)	[89]
10^c^	Ph	Ph	3	– (**j**)	[77]

^a^Isolated yields were shown and in brackets were described the recovery of the starting materials (*E*)-**6**. ^b^Yield determined by ^19^F NMR. ^c^This substrate (*E*)-**6j** contains a CH_3_ group instead of a CF_3_ moiety with CH_3_ and Ph located at the opposite positions.

For the purpose of obtaining mechanistic information on the present reaction, three representative optically active substrates (*R*,*E*)-**6** were selected and submitted to the identical conditions as above (Table 4). Furthermore, in the case of (*R*,*E*)-**6a**, conditions in entries 6 (6 M NaOH aq in MeOH) and 19 (DABCO in toluene) in Table 2 were also tried for comparison (see entries 2 and 3, respectively). Like the case of the corresponding racemic compounds, the DBU-mediated proton shift was realized in a similar fashion to afford the ketones **7** in excellent isolated yields and, moreover, accomplished a quite high degree of chirality transmission (CT) on the basis of the chiral HPLC analysis (Table 4, entries 1, 4, and 5). Unanimous formation of (*R*)-stereoisomers at the 3 position of **7** from (*R*,*E*)-**6** led to confirmation that the proton attached to C^1^ was migrated to C^3^ from its *si* face, thus from the same back side if (*R*,*E*)-**6** possessed its conformation as shown in Table 4. Although DABCO attained the same level of CT (Table 4, entry 3) albeit a slower reaction rate, this is not the case for the conditions of 6 M NaOH aq in MeOH and only 21% CT was observed. The latter result would be because of the competing occurrence of intermolecular reprotonation by the solvent.

**Table 4:** DBU-promoted proton shift of the chiral allylic alcohols (*R*,*E*)-**6**.

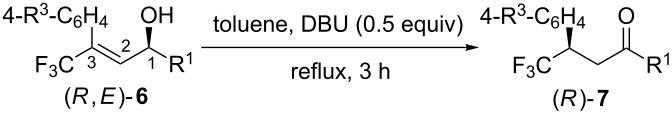					

Entry	R^3^	eeS^a,b^ (% ee)	Yield (%)	eeP^a,b^ (% ee)	CT^c^ (%)

1	H	83	88 (**a**)	85	>99
2^d^	H	86	63 (**a**)	18	21
3^e^	H	87	60 (**a**)	86	99
4	MeO	80	85 (**b**)	77	96
5	F	80	88 (**c**)	78	98

^a^eeS and eeP are the enantiomeric excess values for (*R*,*E*)-**6** and (*R*)-**7**, respectively. ^b^Determined by HPLC analysis using CHIRALPAK OD and AD columns for substrates and products, respectively. ^c^CT: Chirality transmission. ^d^6 M NaOH aq (6 equiv) in MeOH was used instead of DBU and toluene, and the reaction was continued for 24 h (30% of (*R*,*E*)-**6a** was isolated). ^e^DABCO was used instead of DBU and the reaction was continued for 24 h (42% of (*R*,*E*)-**6a** was detected by ^19^F NMR).

**Figure 1:**
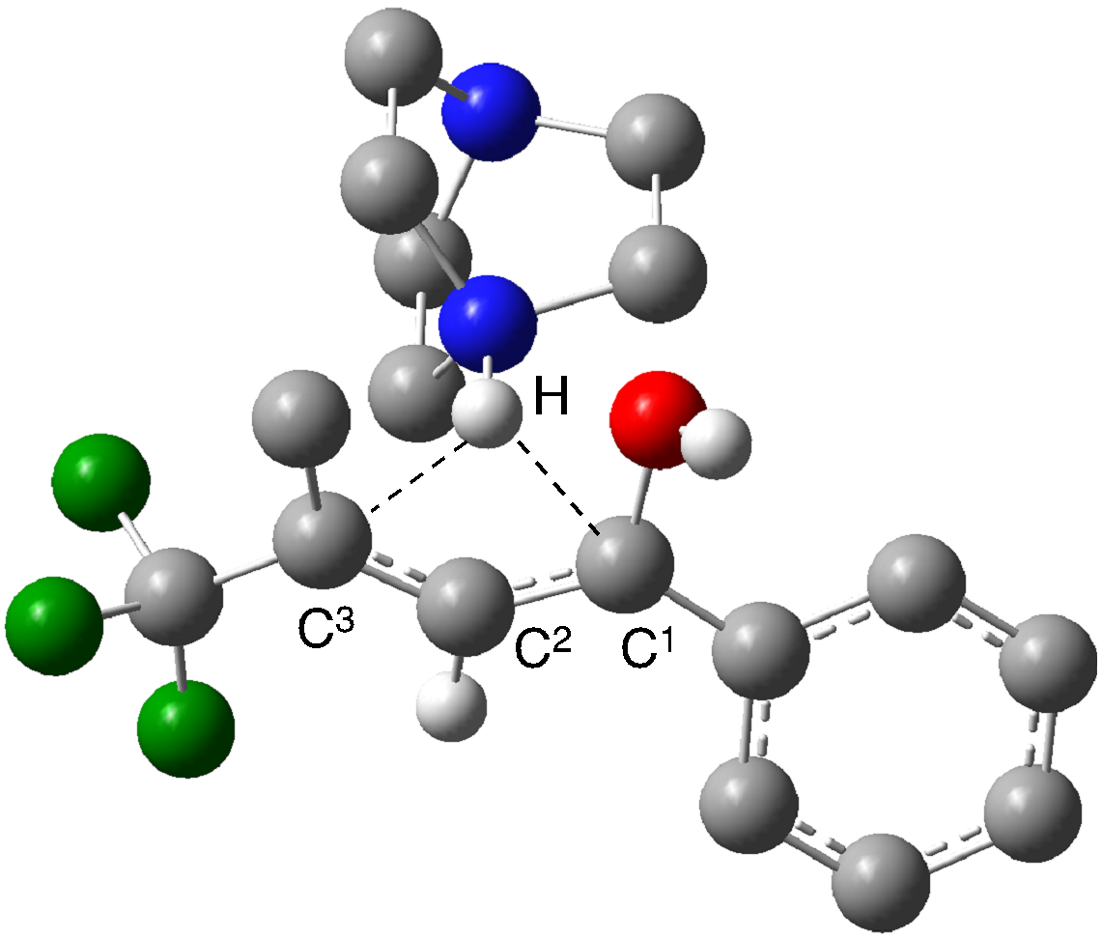
Calculated transition state model TS-**8h** for the present proton shift starting from (*R*,*E*)-**6h** (some hydrogen atoms are omitted for clarity).

The present interesting proton shift reaction was also computationally simulated [7] by employing (*R*,*E*)-**6h** with the substitution pattern of R^1^ = Ph and R^2^ = Me as the model substrate. For simplicity, 1,4-diazabicyclo[2.2.2]octane (DABCO) was employed as the representative base rather than DBU. Successful location of the transition state TS-**8h** at the B3LYP/6-311++G** level of theory led to clear analysis that TS-**8h** was 27.10 kcal/mol higher in energy than the combination of the substrate (*R*,*E*)-**6h** and the reactant DABCO under vacuum. This barrier became lowered to 24.71 kcal/mol after the single point calculation by consideration of the solvent effect of toluene by the conductor-like polarizable continuum model (CPCM). This energy barrier is considered to qualitatively correspond to the requirement of toluene reflux temperature (110–111 °C) for promotion of the desired proton shift. The cleaving C^1^···H and forming C^3^···H bond lengths in TS-**8h** were calculated to be 220.0 and 238.1 pm which were significantly elongated from the ones of the corresponding substrate (*R*,*E*)-**6h** and product (*R*)-**7h** of 109.5 and 109.3 pm, respectively (Figure 1). The partial charge at C^3^ became more negative (–0.338, –0.110, and –0.299 for TS-**8h**, (*R*,*E*)-**6h**, and (*R*)-**7h**, respectively) and H more positive (0.528, 0.172 and 0.244). The DABCO molecule with activating the proton was found to be situated right behind the C^2^ atom with the C^2^···H distance of 201.6 pm. The N···H distance of 104.4 pm was found to be only 2.4 pm longer than the same bond in protonated DABCO which would be one of the major reasons why the weaker base Et_3_N did not work for this reaction.

## Conclusion

As shown above, our original isomerization of CF_3_-containing propargylic alcohols **1F** to the corresponding α,β-unsaturated ketones (*E*)-**5** was successfully extended to the transformation of allylic alcohols (*E*)-**6** to saturated ketones **7**. Different from the previously reported transition metal-catalyzed protocols by Cahard [8–9,13] and Liu [10–12], our transformation nicely proceeded with such a convenient and tractable tertiary amine as DBU, thus under metal-free conditions like the cases of the Ando [14] and Martín-Matute groups [15]. Moreover, this intriguing proton shift was clarified to be applied for optically active allylic alcohols whose chirality was transmitted almost in a perfect fashion. These results should stem from the acidic nature of the allylic proton which was successfully estimated from our independent computation. Further utilization of this reaction is being studied in this laboratory and the results obtained will be reported in due course.

## Supporting Information

File 1 Experimental procedures, characterization data, copies of ^1^H and ^13^C NMR spectra, HPLC charts for optically active compounds, and computational details.
