# Supplementary material for: Base-promoted isomerization of CF3-containing allylic alcohols to the corresponding saturated ketones under metal-free conditions
Source: Beilstein J Org Chem. 2017 Aug 1;13:1507–12. doi: 10.3762/bjoc.13.149 (PMC5550803; doi:10.3762/bjoc.13.149)
Supplement: File 1 — Experimental procedures, characterization data, copies of 1H and 13C NMR spectra, HPLC charts for optically active compounds, and computational details. [file Beilstein_J_Org_Chem-13-1507-s001.pdf]

Supporting Information

for

Base-promoted isomerization of CF_3-containing allylic alcohols to the corresponding saturated ketones under metal-free conditions

Yoko Hamada, Tomoko Kawasaki-Takasuka, and Takashi Yamazaki*

Address: Division of Applied Chemistry, Institute of Engineering, Tokyo University of Agriculture and Technology, 2-24-16 Nakamachi, Koganei 184-8588, Japan

Email: Takashi Yamazaki - tyamazak@cc.tuat.ac.jp

*Corresponding author

Experimental procedures, characterization data, copies of ^{1}H and ^{13}C NMR spectra, HPLC charts for optically active compounds, and computational details

1. General information ········· S2
2. Experimental procedures and characterization data ········· S2
General method for the preparation of Horner–Wadsworth–Emmons reagents ········· S2
General method for the preparation of α,β-unsaturated ketones S4
General procedure for the preparation of allylic alcohols ········· S8
General procedure for the preparation of optically active allylic alcohols ········· S11
General procedure for the isomerization of racemic allylic alcohols (*E*)-6 to the corresponding saturated ketones ········· S13
3. ^{1}H and ^{13}C NMR charts ········· S17
4. HPLC charts ········· S50
5. Computational details ········· S62
6. References ········· S81

1. General information

^{1}H (300.40 MHz), ^{13}C (75.45 Hz), and ^{19}F (282.65 Hz) NMR spectra were recorded on a JEOL AL 300 spectrometer in $CDCl_3$ unless otherwise noted and chemical shifts were recorded in parts per million (ppm), downfield from internal tetramethylsilane (Me_4Si: δ 0.00, for ^{1}H and ^{13}C) or hexafluorobenzene (C_6F_6: δ –163.00 for ^{19}F). Data were tabulated in the following order: number of protons, multiplicity (s, singlet; d, doublet; t, triplet; q, quartet; quint, quintet; sex, sextet; m, multiplet; b, broad peak), coupling constants in Hertz. Infrared (IR) spectra were obtained on a JASCO A-302 spectrometer and reported in wave numbers (cm^{-1}). Elemental analyses were performed by Perkin-Elmer SeriesII CHNS/O analyzer. JEOL JMS-700 was used for obtaining high resolution mass spectrometry data by the positive ionization mode.

Most of reactions where an organic solvent was employed were performed under argon with magnetic stirring using flame-dried glassware. Anhydrous THF, Et_2O, and CH_2Cl_2 were purchased and used without further purification. Unless otherwise noted, materials were obtained from commercial suppliers and were used without further purification. Analytical thin-layer chromatography (TLC) was routinely used for monitoring reactions by generally using a mixture of hexane (Hex) and ethyl acetate (AcOEt) (v/v). Spherical neutral silica gel (63–210 μm or 40–50 μm) was employed for column chromatography and flush chromatography, respectively.

2. Experimental procedures and characterization data

General method for the preparation of Horner–Wadsworth–Emmons reagents: Dimethyl 2-oxo-2-phenylethylphosphonate (9a) [1]

To a 200 mL flame-dried three-necked flask under argon atmosphere were introduced 42.6 mL of BuLi (1.55 M, 66.0 mmol) and 9.7 mL of THF so as to control the concentration to ca 1 M, where 1,1,1,3,3,3-hexamethyldisilazane (14.0 mL, 66.0 mmol) was added slowly and stirring was continued for 15 min at room temperature. Dimethyl methylphosphonate (3.6 mL, 33 mmol) was added with keeping the temperature inside and then, ethyl benzoate (4.3 mL, 30 mmol) was added at lower than 5 °C. When complete consumption of ethyl benzoate was confirmed by TLC, the reaction mixture was quenched with water and extracted three times with AcOEt. The

combined AcOEt phase was further washed with water and brine successively, and dried over anhydrous Na_2SO_4. After filtration, evaporation of the volatiles afforded the crude material which was purified by silica gel column chromatography using AcOEt as an eluent to furnish 6.8 g of the title compound, dimethyl 2-oxo-2-phenylethylphosphonate **9a** was obtained (29.7 mmol, 99% yield) as a color- less oil. Rf = 0.43 (AcOEt). ^{1}H NMR: δ 3.65 (2H, d, *J* = 22.5 Hz), 3.79 (6H, d, *J* = 11.1 Hz), 7.46-7.52 (2H, m), 7.58-7.63 (1H, m), 7.99-8.02 (2H, m). ^{13}C NMR: δ 37.1 (d, *J* = 130.9 Hz), 52.8 (d, *J* = 6.9 Hz), 128.5, 128.7, 133.6, 136.1 (d, *J* = 2.5 Hz), 191.5 (d, *J* = 6.8 Hz).

Dimethyl 2-oxo-4-phenylbutylphosphonate (9b) [2]

Ethyl 3-phenylpropionate (5.1 mL, 30.0 mmol) and diisopropylamine (9.3 mL, 66.0 mmol) were employed instead of ethyl benzoate and 1,1,1,3,3,3-hexamethyldisilazane, and 7.3 g of dimethyl 2-oxo-4-phenylbutylphosphonate **9b** was obtained (28.6 mmol, 95% yield) as a colorless oil. Rf = 0.30 (AcOEt). ^{1}H NMR: δ 2.88-3.00 (4H, m), 3.08 (2H, d, *J* = 22.8 Hz), 3.75 (6H, d, *J* = 11.1 Hz), 7.18-7.31 (5H, m). ^{13}C NMR: δ 29.1, 41.2 (d, *J* = 127.8 Hz), 45.3 (d, *J* = 1.8 Hz), 52.8 (d, *J* = 6.8 Hz), 125.9, 128.1, 128.2, 140.3, 200.7 (d, *J* = 6.2 Hz).

Dimethyl 2-oxobutylphosphonate (9c) [3]

Ethyl propionate (3.4 mL, 30.0 mmol) was employed instead of ethyl benzoate, and 3.8 g of dimethyl 2-oxobutylphosphonate **9c** was obtained (19.6 mmol, 65% yield) as a colorless oil. Rf = 0.43 (AcOEt). ^{1}H NMR: δ 1.07 (3H, t, *J* = 7.2 Hz), 2.66 (2H, q, *J* = 7.0 Hz), 3.11 (2H, d, *J* = 22.5 Hz), 3.79 (6H, d, *J* = 11.1 Hz). ^{13}C NMR: δ 7.2, 37.2 (d, *J* = 1.9 Hz), 40.7 (d, *J* = 128.4 Hz), 52.7 (d, *J* = 6.8 Hz), 202.2 (d, *J* = 6.2 Hz).

Dimethyl 2-(4-methoxyphenyl)-2-oxoethylphosphonate (9d) [4]

Ethyl *p*-anisate (5.4 g, 30.0 mmol) was employed instead of ethyl benzoate, and 7.4 g of dimethyl 2-(4-methoxyphenyl)-2-oxoethylphosphonate **9d** was obtained (28.8 mmol, 96% yield) as a colorless oil. Rf = 0.20 (AcOEt). ^{1}H NMR: δ 3.60 (2H, d, *J* = 55.5 Hz), 3.78 (6H, d, *J* = 11.1 Hz), 3.88 (3H, s), 6.93-6.98 (2H,m), 7.97-8.02 (2H, m). ^{13}C NMR: δ 37.2 (d, *J* = 130.9 Hz), 53.1 (d, *J* = 6.2 Hz), 55.5, 113.8, 129.4, 131.4, 164.0, 190.0 (d, *J* = 8.1 Hz).

Dimethyl 2-(4-nitrophenyl)-2-oxoethylphosphonate (9e) [5]

Ethyl 4-nitrobenzoate (2.0 g, 10.0 mmol) was employed instead of ethyl benzoate, and 2.1 g of dimethyl 2-(4-nitrophenyl)-2-oxoethylphosphonate **9e** was obtained (7.6

mmol, 76% yield) as a yellow-colored oil. Rf = 0.19 (AcOEt). ^{1}H NMR: δ 3.69 (2H, d, *J* = 23.1 Hz), 3.81 (6H, d, *J* = 11.1 Hz), 8.26-8.22 (2H, m), 8.32-8.37 (2H, m). ^{13}C NMR: δ 37.8 (d, *J* = 130.2 Hz), 53.1 (d, *J* = 6.8 Hz), 123.6, 129.9, 140.3 (d, *J* = 1.9 Hz), 150.3, 190.3 (d, *J* = 6.8 Hz).

Dimethyl 2-(4-bromophenyl)-2-oxoethylphosphonate (9f)

Ethyl 4-bromobenzoate (6.9 g, 30.0 mmol) was employed instead of ethyl benzoate, and 9.0 g of dimethyl 2-(4-bromophenyl)-2-oxoethylphosphonate **9f** was obtained (29.4 mmol, 98% yield) as a colorless oil. Rf = 0.23 (AcOEt). ^{1}H NMR: δ 3.61 (2H, d, *J* = 22.5 Hz), 3.79 (6H, d, *J* = 11.4 Hz), 7.62-7.86 (2H, m), 7.87-7.89 (2H, m). ^{13}C NMR: δ 36.6 (d, *J* = 130.2 Hz), 52.4 (d, *J* = 6.2 Hz), 128.3, 129.8, 131.2, 134.3 (d, *J* = 2.5 Hz), 190.2 (d, *J* = 6.8 Hz). IR (neat): ν 3466, 2997, 2956, 2853, 1682, 1586, 1568, 1486, 1462, 1397, 1256. HRMS (FAB+, *m/z*): $[M+2H]^+$ calcd for $C_{10}H_{14}BrO_4P$, 307.9813; Found, 307.9871.

General method for the preparation of α,β-unsaturated ketones: (*E*)-4,4,4-Trifluoro-1,3-diphenylbut-2-en-1-one ((*E*)-10a) [6,7,8,9]

To a flame-dried two-necked 200 mL flask containing ethyl trifluoroacetate (2.4 mL, 20.0 mmol) and 40.0 mL of Et_2O was added PhMgBr (prepared in Et_2O, 0.95 *M*, 21.0 mL, 20.0 mmol) under an argon atmosphere at –80 °C, then the mixture was stirred for 1 h at that temperature and another 1 h at 0 °C with the aid of an ice bath where 1.8 mL of H_2O (100.0 mmol) was added.

To a different two-necked flask containing 2.8 g of lithium bromide (32.0 mmol) and THF (60 mL) were added 6.0 g of dimethyl 2-oxo-2-phenylethylphosphonate (26.0 mmol) and 3.8 mL of triethylamine (28.0 mmol) at 0 °C under argon atmosphere, and the whole mixture was stirred for 10 min at room temperature. After cooling to 0 °C, the above solution was introduced to this flask with the aid of cannula, and the resultant mixture was stirred for 5 h at 40 °C. 1 M HCl aq. was added to this mixture which was further extracted three times with AcOEt. To the combined AcOEt phase was added anhydrous $MgSO_4$ and after filtration, concentration and purification by silica gel chromatography (Hex:AcOEt = 20:1) furnished 4.1 g of the title compound **(*E*)-10a** (14.7 mmol, 73% yield, *E* only) as a yellow oil. Rf = 0.54 (Hex:AcOEt = 4:1). ^{1}H NMR: δ 7.27-7.28 (6H, m), 7.37-7.43 (2H, m), 7.50-7.56 (1H, m), 7.81-7.84 (2H, m). ^{13}C NMR: δ 122.8 (q, *J* = 274.8 Hz), 128.3, 128.6, 128.8, 129.0, 129.3, 130.8, 130.8 (q, *J* = 5.0 Hz), 133.8, 136.0, 138.8(q, *J* = 30.8 Hz), 192.0. ^{19}F NMR: δ –67.54 (s).

4,4,4-Trifluoro-3-(4-methoxyphenyl)-1-phenylbut-2-en-1-one (10b) [6,7,8,9,10]

Dimethyl 2-oxo-2-phenylethylphosphonate (9.4 g, 39.0 mmol) and *p*-MeO-C_6H_4MgBr/THF were employed instead of dimethyl 2-oxo-2-phenylethyl-phosphonate and PhMgBr/Et_2O, respectively, and chromatographic separation afforded the pure **10b** (*E* form: 4.9 g, 15.9 mmol, *Z* form: 0.74 g, 2.4 mmol, *E*:*Z* = 96:4).

(*E*)-10b Yield: 57% (yellow oil)**,** Rf = 0.40 (Hex:AcOEt = 10:1). ^{1}H NMR: δ 3.75 (3H, s), 6.78 (2H, dt, *J* = 9.3, 2.6 Hz), 7.20-7.21 (2H, m), 7.23 (1H, s), 7.38-7.43 (2H, m), 7.51-7.56 (1H, m), 7.82-7.85 (2H, m). ^{13}C NMR: δ 55.0, 113.8, 122.85, 122.92 (q, *J* = 274.4 Hz), 128.6, 128.9, 130.1 (q, *J* = 5.2 Hz), 130.4, 133.8, 135.9, 138.3 (q, *J* = 30.6 Hz), 160.3, 192.5. ^{19}F NMR: δ –67.55 (s).

(*Z*)-10b Yield: 9% (yellow oil), Rf = 0.22 (*n*-Hex:AcOEt = 10:1). ^{1}H NMR: δ 3.85 (3H, s), 6.78 (1H, s), 6.97 (2H, d, *J* = 9.0 Hz), 7.48-7.54 (4H, m), 7.61-7.66 (1H, m), 7.98-8.01 (2H, m). ^{13}C NMR: δ 55.2, 114.1, 122.7 (q, *J* = 276.0 Hz), 125.6, 128.7, 129.0, 132.9 (q, *J* = 3.7 Hz), 134.0, 135.3 (q, *J* = 31.0 Hz), 135.64, 135.66, 160.6, 192.6. ^{19}F NMR: δ –60.58 (s). IR (neat): 2937, 2841, 1675, 1609, 1515, 1450, 1363, 1171, 1035, 834, 752. HRMS (FAB+, *m/z*): $[M+H]^+$ calcd for $C_{17}H_{14}F_3O$, 307.0946; Found, 307.0970.

(*E*)-4,4,4-Trifluoro-3-(4-fluorophenyl)-1-phenylbut-2-en-1-one ((*E*)-10c) [8,10,11]

Dimethyl 2-oxo-2-phenylethylphosphonate (6.3 g, 27.5 mmol) and *p*-F-C_6H_4MgBr·LiBr/Et_2O [12] were employed instead of dimethyl 2-oxo-2-phenyl-ethylphosphonate and PhMgBr/Et_2O, respectively, and chromatographic separation afforded 2.3 g of the pure **(*E*)-10c** (7.7 mmol, 37% yield, *E* only) as a yellow oil. Rf = 0.34 (Hex: AcOEt = 15:1). ^{1}H NMR: δ 6.93-6.99 (2H, m), 7.23-7.30 (3H, m), 7.39-7.44 (2H, m), 7.53-7.58 (1H, m), 7.81-7.83 (2H, m). ^{13}C NMR: δ 115.5 (d, *J* = 21.7 Hz), 122.7 (qd, *J* = 274.2, 1.2 Hz), 126.7 (d, *J* = 3.7 Hz), 128.7, 128.8, 131.1 (d, *J* = 8.7 Hz), 131.2 (q, *J* = 4.9 Hz), 134.0, 135.9, 137.8 (q, *J* = 31.0 Hz), 163.2 (d, *J* = 249.3 Hz), 191.7. ^{19}F NMR: δ –67.86 (s), –112.34 to –112.48 (m).

(*E*)-1-Phenyl-3-(trifluoromethyl)pent-2-en-1-one ((*E*)-10d) [11]

Dimethyl 2-oxo-2-phenylethylphosphonate (9.4 g, 39.0 mmol) and EtMgBr /Et_2O were employed instead of dimethyl 2-oxo-2-phenylethylphosphonate and PhMgBr/Et_2O, respectively, and chromatographic separation afforded 4.3 g of the pure **(*E*)-10d** (18.8 mmol, 63% yield, *E* only) as a yellow oil. Rf = 0.57 (Hex:AcOEt = 20:1). ^{1}H NMR: δ 1.20 (3H, t, *J* = 7.5 Hz), 2.57 (2H, q, *J* = 7.5 Hz), 7.22 (1H, q, *J* = 1.5 Hz), 7.49-7.54

(2H, m), 7.60-7.63 (1H, m), 7.93-7.96 (2H, m). ^{13}C NMR: δ 13.4, 20.7, 123.8 (q, *J* = 274.8 Hz), 126.1 (q, *J* = 5.6 Hz), 128.5, 128.8, 133.7, 137.1, 144.6 (q, *J* = 28.5 Hz), 190.9. ^{19}F NMR: δ –69.77 (s).

(*E*)-1,5-Diphenyl-3-(trifluoromethyl)pent-2-en-1-one ((*E*)-10e)

Dimethyl 2-oxo-2-phenylethylphosphonate (9.4 g, 39.0 mmol) and $Ph(CH_2)_2MgBr/Et_2O$ were employed instead of dimethyl 2-oxo-2-phenylethylphosphonate and $PhMgBr/Et_2O$, respectively, and chromatographic separation afforded 7.5 g of the pure **(*E*)-10e** (24.8 mmol, 83% yield, *E* only) as a yellow oil. Rf = 0.46 (Hex: AcOEt = 20:1). ^{1}H NMR: δ 2.87 (4H, s), 7.13 (1H, sex, *J* = 4.2 Hz), 7.24-7.29 (5H, m), 7.46-7.52 (2H, m), 7.58-7.61 (1H, m), 7.87-7.90 (2H, m). ^{13}C NMR: δ 29.4, 34.9, 123.7 (q, *J* = 274.8 Hz), 126.2, 126.9 (q, *J* = 5.6 Hz), 128.38, 128.43 (2C), 128.7, 133.7, 137.0, 140.5, 142.5 (q, *J* = 28.6 Hz), 190.6. ^{19}F NMR: δ –69.65 (s). IR (neat): ν 3064, 3030, 2944, 2872, 1676, 1597, 1496, 1450, 1233, 1126, 749, 701. Calcd for $C_{18}H_{15}F_3O$: C, 71.04; H, 4.97. Found: C, 71.33; H, 4.99.

(*E*)-1-(4-Methoxyphenyl)-5-phenyl-3-(trifluoromethyl)pent-2-en-1-one ((*E*)-10f)

Dimethyl 2-(4-methoxyphenyl)-2-oxoethylphosphonate (3.3 mg, 13 mmol) and $Ph(CH_2)_2MgBr/Et_2O$ were employed instead of dimethyl 2-oxo-2-phenylethylphosphonate and $PhMgBr/Et_2O$, respectively, and chromatographic separation afforded 2.5 g of the pure **(*E*)-10g** (7.4 mmol, 78% yield, *E* only) as a white solid. mp 61.6 °C, Rf = 0.37 (Hex:AcOEt = 20:1). ^{1}H NMR: δ 2.79-2.92 (4H, m), 3.90 (3H, s), 6.96 (2H, dt, *J* = 9.3, 2.4 Hz), 7.10-7.17 (1H, m), 7.21-7.26 (5H, m), 7.86-7.91 (2H, m). ^{13}C NMR: δ 29.4, 35.0, 55.4, 113.9, 123.8 (q, *J* = 274.7 Hz), 126.1, 127.3 (q, *J* = 5.6 Hz), 128.3, 128.4, 130.1, 130.9, 140.7, 141.4 (q, *J* = 28.5 Hz), 164.0, 189.1. ^{19}F NMR: δ –69.56 (s). IR (KBr): ν 3070, 3030, 2974, 2938, 1752, 1673, 1636, 1595, 1509, 1456, 1264, 1054. Calcd for $C_{19}H_{17}F_3O_2$: C, 68.26; H, 5.13. Found: C, 68.50; H, 5.13.

(*E*)-1-(4-Bromophenyl)-5-phenyl-3-(trifluoromethyl)pent-2-en-1-one ((*E*)-10g)

Dimethyl 2-(4-bromophenyl)-2-oxoethylphosphonate (2.3 g, 7.4 mmol) and $Ph(CH_2)_2MgBr/Et_2O$ were employed instead of dimethyl 2-oxo-2-phenylethylphosphonate and $PhMgBr/Et_2O$, respectively, and chromatographic separation afforded 1.8 g of the pure **(*E*)-9g** (4.7 mmol, 63% yield, *E* only) as a white solid. mp 55.7 °C, Rf = 0.31 (Hex:AcOEt = 20:1). ^{1}H NMR: δ 2.88 (4H, s), 7.12 (1H, m), 7.21-7.23 (5H, m), 7.60-7.64 (2H, m), 7.70-7.75 (2H, m). ^{13}C NMR: δ 29.3, 34.8, 123.6 (q, *J* = 274.7 Hz), 126.2, 126.3 (q, *J* = 5.4 Hz), 128.4, 128.5, 129.0, 129.9, 132.1, 135.7, 140.3, 143.2

(q, J = 29.2 Hz), 189.5. ^{19}F NMR: δ –69.67 (s). IR (KBr): ν 3301, 3087, 3032, 2943, 2873, 1919, 1664, 1586, 1136, 813, 703. Calcd for $C_{18}H_{14}BrF_3O$: C, 56.42; H, 3.68. Found: C, 56.81; H, 3.86.

6,6,6-Trifluoro-1,5-diphenylhex-4-en-3-one (10h)

Dimethyl 2-oxo-4-phenylbutylphosphonate (9.5 g, 37 mmol) was employed instead of dimethyl 2-oxo-2-phenylethylphosphonate, and chromatographic separation afforded 4.76 g of the pure **9h** (*E* form: 4.4 g, 14.5 mmol, *Z* form: 0.36 g, 1.2 mmol, *E*:*Z* = 93:7).

(*E*)-10h Yield: 51% (yellow oil), Rf = 0.42 (Hex:AcOEt = 10:1). ^{1}H NMR: δ 2.49-2.54 (2H, m), 2.74 (2H, t, J = 7.5 Hz), 6.71 (1H, q, J = 1.4 Hz), 6.95-6.98 (2H, m), 7.16-7.25 (5H, m), 7.36-7.48 (3H, m). ^{13}C NMR: δ 29.2, 44.5, 122.7 (q, J = 274.1 Hz), 126.0, 128.1, 128.3, 128.6, 128.9, 129.6, 130.7, 131.3 (q, J = 5.0 Hz), 138.3 (q, J = 28.1 Hz), 140.1, 200.6. ^{19}F NMR: δ –68.32 (s). IR (neat): ν 3088, 3028, 2930, 1708, 1603, 1496, 1455, 1281, 1178, 749, 700. Calcd for $C_{18}H_{15}F_3O$: C, 71.04; H, 4.97. Found: C, 71.01; H, 4.96.

(*Z*)-10h Yield: 4% (yellow oil), Rf = 0.28 (Hex:AcOEt = 10:1). ^{1}H NMR: δ 2.99 (4H, s), 6.40 (1H, s), 7.21-7.39 (10H, m). ^{13}C NMR: δ 29.2, 44.7 (q, J = 1.2 Hz), 122.5 (q, J = 276.1 Hz), 126.2, 127.6, 128.3, 128.5, 128.6, 129.4, 133.3, 134.6 (q, J = 31.6 Hz), 135.9 (q, J = 3.7 Hz), 140.3, 201.1. ^{19}F NMR: δ –60.38 (s). IR (neat): 3029, 1710, 1604, 1496, 1454, 1364, 1282, 1169, 1132, 762, 698. HRMS (FAB+, *m/z*): $[M+H]^+$ calcd for $C_{18}H_{16}F_3O$, 305.1153; Found, 305.1179.

(*E*)-6,6,6-Trifluoro-5-phenylhex-4-en-3-one ((*E*)-10i) [13]

Dimethyl 2-oxobutylphosphonate (7.4 g, 39 mmol) was employed instead of dimethyl 2-oxo-2-phenylethylphosphonate, and chromatographic separation afforded 4.4 g of the pure **(*E*)-10i** (19.5 mmol, 66% yield, *E* only) as a yellow oil. Rf = 0.34 (Hex:AcOEt = 20:1). ^{1}H NMR: δ 0.91 (3H, t, J = 7.2 Hz), 2.23 (2H, q, J = 7.2 Hz), 6.75 (1H, s), 7.30-7.44 (5H, m). ^{13}C NMR: δ 13.4, 20.7, 123.8 (q, J = 274.8 Hz), 126.1 (q, J = 5.6 Hz), 128.5, 128.8, 133.7, 137.1, 144.6 (q, J = 28.5 Hz), 190.9. ^{19}F NMR: δ –68.39 (s). IR (neat): ν 3734, 2981, 2941, 1711, 1496, 1409, 1281, 1179, 944, 770, 705. HRMS (FAB+, *m/z*): $[M]^+$ calcd for $C_{12}H_{11}F_3O$, 228.0762; Found, 228.0776.

(*E*)-1,3-Diphenylbut-2-en-1-one ((*E*)-10j) [14,15]

To a flame-dried two-necked 50 mL flask containing 0.58 mL of acetophenone

(5.0 mmol) and 10 mL of CH_2Cl_2 were added $TiCl_4$ (0.60 mL, 5.5 mmol) and 1.4 mL of tributylamine (6.0 mmol), and the whole mixture was stirred for 30 min at room temperature. After addition of 0.58 mL of acetophenone (5.0 mmol) and stirring for 1 h at the same temperature, 2.0 mL of pyridine (25.0 mmol) was added and the mixture was stirred for 5 h at room temperature. Filtration of the reaction mixture after addition of Et_2O and hexane (25 mL each) with the aid of Celite and concentration of the organic phase furnished crude mixture which was chromatographed on silica gel using a mixture of Hex:AcOEt = 10:1 as an eluent to give 0.35 g of the title compound **(*E*)-9j** (1.6 mmol, 32% yield, *E* only) as a yellow oil. Rf = 0.57 (Hex:AcOEt = 6:1). ^{1}H NMR: δ 2.60 (s, 3H), 7.17 (s, 1H), 7.39-7.59 (m, 8H), 8.00-8.01 (m, 2H). ^{13}C NMR: δ 18.7, 121.9, 126.3, 128.1, 128.4, 128.5, 129.0, 132.4, 139.2, 142.6, 154.9, 191.2.

General procedure for the preparation of allylic alcohols: racemic (*E*)-4,4,4-tri-fluoro-1,3-diphenylbut-2-en-1-ol ((*E*)-6a) [6,16,17,18]

To a flame-dried 100 mL round-bottomed flask containing (*E*)-4,4,4-trifluoro-1,3-diphenylbut-2-en-1-one **(*E*)-10a** (2.8 g, 10.0 mmol) and MeOH (50 mL) was added at 0 °C 0.38 g of $NaBH_4$ (10.0 mmol) all at once and stirred the reaction for 10 min at the same temperature. After addition of water and evaporation of the volatiles, the mixture was extracted with AcOEt three times and the combined AcOEt phase was dried with anhydrous Na_2SO_4 and concentrated. The resultant crude material was purified by silica gel column chromatography using a mixture of Hex:AcOEt = 4:1 as an eluent to afford 2.8 g of **(*E*)-6a** (10.0 mmol) in quantitative yield as a colorless oil. Rf = 0.30 (Hex:AcOEt = 4:1). ^{1}H NMR: δ 1.94 (1H, d, *J* = 3.6 Hz), 5.14 (1H, dd, *J* = 9.3, 3.3 Hz), 6.61 (1H, dq, *J* = 9.2, 1.5 Hz), 7.28–7.45 (10H, m). ^{13}C NMR: δ 70.3, 123.1 (q, *J* = 272.1 Hz), 126.1, 128.3, 128.5, 128.8, 129.0. 129.6. 131.3, 132.0 (q, *J* = 30.1 Hz), 136.5 (q, *J* = 5.2 Hz), 141.5. ^{19}F NMR: δ –67.81 (s).

(*E*)-4,4,4-Trifluoro-3-(4-methoxyphenyl)-1-phenylbut-2-en-1-ol ((*E*)-6b) [7,8,17,19]

(*E*)-4,4,4,-Trifluoro-3-(4-methoxyphenyl)-1-phenylbut-2-en-1-one (0.91 g, 3.0 mmol) was employed instead of (*E*)-4,4,4-trifluoro-1,3-diphenylbut-2-en-1-ol and chromatography on silica gel using a mixture of Hex:AcOEt = 3:1 as an eluent afforded the title compound 0.91 g of **(*E*)-6b** (2.9 mmol) in 96% yield as a colorless oil. Rf = 0.49 (Hex:AcOEt = 3:1). ^{1}H NMR: δ 1.94 (1H, d, *J* = 3.6 Hz), 3.85 (3H, s), 5.17 (1H, dd, *J* = 9.3, 3.3 Hz), 6.58 (1H, dq, *J* = 9.3, 1.5 Hz), 6.92-6.97 (2H, m), 7.18-7.22 (2H,

m), 7.26-7.40 (5H, m). ^{13}C NMR: δ 55.1, 70.3, 113.9, 123.3, 126.1, 126.8 (q, *J* = 281.0 Hz), 128.2, 128.8, 130.8, 131.6 (q, *J* = 32.0 Hz), 136.3 (q, *J* = 5.2 Hz), 141.6, 159.9. ^{19}F NMR: δ –67.97 (s).

(*E*)-4,4,4-Trifluoro-3-(4-fluorophenyl)-1-phenylbut-2-en-1-ol ((*E*)-6c) [20,21]

(*E*)-1-Phenyl-3-(trifluoromethyl)pent-2-en-1-one (2.1 g, 7.2 mmol) was employed instead of (*E*)-4,4,4-trifluoro-1,3-diphenylbut-2-en-1-ol and chromatography on silica gel using a mixture of Hex:AcOEt = 6:1 as an eluent afforded the title compound 2.1 g of **(*E*)-6c** (7.2 mmol) in 99% yield as a colorless oil. Rf = 0.29 (Hex:AcOEt = 6:1). ^{1}H NMR: δ 2.05 (1H, s), 5.08 (1H, dd, *J* = 9.3, 2.1 Hz), 6.62 (1H, dq, *J* = 9.0, 1.8 Hz), 7.07-7.15 (2H, m), 7.22-7.39 (7H, m). ^{13}C NMR: δ 70.4, 115.7 (d, *J* = 21.7 Hz), 122.9 (q, *J* = 273.6 Hz), 126.1 (d, *J* = 1.2 Hz), 127.1 (q, *J* = 3.7 Hz), 128.4, 128.9, 131.0 (q, *J* = 31.0Hz), 131.5 (d, *J* = 8.7 Hz), 137.0 (q, *J* = 5.6 Hz), 141.3, 163.1 (d, *J* = 248.8 Hz). ^{19}F NMR: δ –67.99 (s), –113.23 to –113.32 (m). IR (neat): ν 3339, 2920, 1605, 1513, 1494, 1455, 1174, 1014, 930, 842, 700. HRMS (FAB+, *m/z*): $[M+H]^+$ calcd for $C_{16}H_{13}F_4O$, 297.0903; found, 297.0899.

(*E*)-1-Phenyl-3-(trifluoromethyl)pent-2-en-1-ol ((*E*)-6d)

(*E*)-1-Phenyl-3-(trifluoromethyl)pent-2-en-1-one (0.69 g, 3.0 mmol) was employed instead of (*E*)-4,4,4-trifluoro-1,3-diphenylbut-2-en-1-ol and chromatography on silica gel using a mixture of Hex:AcOEt = 3:1 as an eluent afforded the title compound 0.66 g of **(*E*)-6d** (2.9 mmol) in 96% yield as a colorless oil. Rf = 0.51 (Hex:AcOEt = 3:1). ^{1}H NMR: δ 1.12 (3H, t, *J* = 7.7 Hz), 1.97 (1H, d, *J* = 3.6 Hz), 2.36 (2H, qd, *J* = 7.5, 2.1 Hz), 5.52 (1H, dd, *J* = 8.6, 2.9 Hz), 6.30 (1H, dq, *J* = 8.9, 1.4 Hz), 7.33-7.40 (5H, m). ^{13}C NMR: δ 13.7, 19.3, 69.5, 124.2 (q, *J* = 273.6 Hz), 126.2, 128.2, 128.8, 132.2 (q, *J* = 28.1 Hz), 134.4 (q, *J* = 5.8 Hz), 141.7. ^{19}F NMR: δ –68.82 (s). IR (neat): ν 3324, 3033, 2980, 1455, 1321, 1254, 1175, 1121, 927, 699. Calcd for $C_{12}H_{13}F_3O$: C, 62.60; H, 5.69. Found: C, 62.49; H, 5.66.

(*E*)-1,5-Diphenyl-3-(trifluoromethyl)pent-2-en-1-ol ((*E*)-6e)

(*E*)-1,5-Diphenyl-3-(trifluoromethyl)pent-2-en-1-one (0.93 g, 3.0 mmol) was employed instead of (*E*)-4,4,4-trifluoro-1,3-diphenylbut-2-en-1-ol and chromatography on silica gel using a mixture of Hex:AcOEt = 3:1 as an eluent afforded the title compound 0.89 g of **(*E*)-6e** (2.9 mmol) in 95% yield as a colorless oil. Rf = 0.46 (Hex:AcOEt = 3:1). ^{1}H NMR: δ 1.17 (1H, d, *J* = 3.3 Hz), 2.60-2.76 (2H, m), 2.84 (2H, td, *J* = 6.9 Hz), 5.08 (1H, dd, *J* = 9.0, 3.3 Hz), 6.25 (1H, d, *J* = 9.3 Hz), 7.22-7.38 (10H,

m). ^{13}C NMR: δ 28.0, 34.5, 69.4, 124.3 (q, J = 274.0 Hz), 125.9, 126.5, 128.1, 128.6, 128.7, 128.8, 129.1 (q, J = 27.9 Hz), 136.4 (q, J = 5.8 Hz), 140.6, 141.2. ^{19}F NMR: δ –67.81 (s). IR (neat): ν 3379, 3029, 2939, 1495, 1454, 1325, 1118, 903, 747, 699. Calcd for $C_{18}H_{17}F_3O$: C, 70.58; H, 5.59. Found: C, 70.48; H, 5.59.

(*E*)-1-(4-Methoxyphenyl)-5-phenyl-3-(trifluoromethyl)pent-2-en-1-ol ((*E*)-6f)

(*E*)-1-(4-Methoxyphenyl)-5-phenyl-3-(trifluoromethyl)pent-2-en-1-on (1.9 g , 5.7 mmol) was employed instead of (*E*)-4,4,4-trifluoro-1,3-diphenylbut-2-en-1-ol and chromatography on silica gel using a mixture of Hex:AcOEt = 3:1 as an eluent afforded the title compound 1.8 g of **(*E*)-6f** (5.3 mmol) in 93% yield as a colorless oil. Rf = 0.43 (Hex:AcOEt = 3:1). ^{1}H NMR: δ 1.15 (1H, d, J = 3.0 Hz), 2.57-2.72 (2H, m), 2.82 (2H, t, J = 7.5 Hz), 3.79 (3H, s), 5.03 (1H, dd, J = 9.0, 3.0 Hz), 6.26 (1H, dq, J = 9.0, 1.5 Hz), 6.84-6.89 (2H, m), 7.14-7.19 (2H, m), 7.22-7.29 (3H, m), 7.33-7.38 (2H, m). ^{13}C NMR: δ 28.0, 34.5, 55.2, 69.1, 114.0, 124.3 (q, J = 273.6 Hz), 126.5, 127.3, 128.50 (q, J = 28.5 Hz), 128.54, 128.8, 133.4, 136.5 (q, J = 5.8 Hz), 140.6, 159.3. ^{19}F NMR: δ –67.84 (s). IR (neat): ν 3375, 2938, 1611, 1513, 1455, 1252, 1164, 1116, 1035, 906, 833, 761. Calcd for $C_{19}H_{19}F_3O_2$: C, 67.85; H, 5.69. Found: C, 67.99; H, 5.64.

(*E*)-1-(4-Bromophenyl)-5-phenyl-3-(trifluoromethyl)pent-2-en-1-ol ((*E*)-6g)

(*E*)-1-(4-Bromophenyl)-5-phenyl-3-(trifluoromethyl)pent-2-en-1-one (1.1 g, 3.0 mmol) was employed instead of (*E*)-4,4,4-trifluoro-1,3-diphenylbut-2-en-1-ol and chromatography on silica gel using a mixture of Hex:AcOEt = 4:1 as an eluent afforded the title compound 0.99 g of **(*E*)-6g** (2.6 mmol) in 86% yield as a colorless oil. Rf = 0.42 (Hex:AcOEt = 4:1). ^{1}H NMR: δ 1.07−1.08 (1H, m), 2.61-2.94 (4H, m), 4.96 (1H, dd, J = 9.0, 3.0 Hz),6.14 (1H, dq, J = 9.3, 1.2 Hz), 7.04- 7.45 (9H, m). ^{13}C NMR: δ 27.9, 34.4, 68.7, 121.9, 124.2 (q, J = 274.2 Hz), 126.7, 127.5, 128.6, 129.0, 129.4, 131.7, 135.9 (q, J = 5.6 Hz), 139.9, 140.4. ^{19}F NMR: δ –67.72 (s). IR (neat): ν 3556, 3392, 3028, 2934, 1487, 1454, 1326, 1187, 1163, 1119, 1011. Calcd for $C_{18}H_{16}BrF_3O$: C, 56.12; H, 4.19. Found: C, 56.33; H, 4.53.

(*E*)-6,6,6-Trifluoro-1,5-diphenylhex-4-en-3-ol ((*E*)-6h)

(*E*)-6,6,6-Trifluoro-1,5-diphenylhex-4-en-3-one (0.91 g, 3.0 mmol) was employed instead of (*E*)-4,4,4-trifluoro-1,3-diphenylbut-2-en-1-ol and chromatography on silica gel using a mixture of Hex:AcOEt = 4:1 as an eluent afforded the title compound 0.92 g of **(*E*)-6h** (3.0 mmol) in quantitative yield as a colorless oil. Rf = 0.40 (Hex:AcOEt = 4:1). ^{1}H NMR: δ 1.54 (1H, s), 1.76-1.99 (2H, m), 2.53-2.74 (2H, m), 4.09-4.17 (1H,

m), 6.40 (1H, dq, J = 7.5, 1.5 Hz), 7.07-7.38 (10H, m). ^{13}C NMR: δ 31.1, 38.1, 67.4, 123.0 (q, J = 270.7 Hz), 125.9, 128.2, 128.4, 128.5, 128.8, 129.3, 131.3, 132.1 (q, J = 41.3 Hz), 137.5 (q, J = 4.8 Hz), 141.0. ^{19}F NMR: δ –67.79 (s). IR (neat): ν 3346, 3027, 2932, 2863, 1496, 1455, 1275, 1121, 750, 704. Calcd for $C_{18}H_{17}F_3O$: C, 70.58; H, 5.59. Found: C, 70.13; H, 5.79.

(*E*)-6,6,6-Trifluoro-5-phenylhex-4-en-3-ol ((*E*)-6i)

(*E*)-6,6,6-Trifluoro-5-phenylhex-4-en-3-one (0.68 g, 3.0 mmol) was employed instead of (*E*)-4,4,4-trifluoro-1,3-diphenylbut-2-en-1-ol and chromatography on silica gel using a mixture of Hex:AcOEt = 4:1 as an eluent afforded the title compound 0.64 g of **(*E*)-6i** (2.8 mmol) in 92% yield as a white solid. mp 33.9 °C, Rf = 0.40 (Hex: AcOEt = 4:1). ^{1}H NMR: δ 0.88 (3H, t, J = 7.5 Hz), 1.47-1.67 (3H m), 4.00 (1H, m), 6.35 (1H, dq, J = 9.2, 1.5 Hz), 7.24-7.27 (2H, m), 7.38-7.43 (3H, m). ^{13}C NMR: δ 9.3, 29.7, 69.3, 123.1 (q, J = 272.6 Hz), 128.5, 128.8, 129.5, 131.5, 132.3 (q, J = 29.4 Hz), 137.4 (q, J = 4.8 Hz). ^{19}F NMR: δ –67.89 (s). IR (KBr): ν 3313, 2977, 2936, 1174, 1119, 1058, 1014, 945, 903, 876, 774. Calcd for $C_{12}H_{13}F_3O$: C, 62.60; H, 5.69. Found: C, 62.78; H, 5.77.

(*E*)-1,3-Diphenylbut-2-en-1-ol ((*E*)-6j) [22]

(*E*)-1,3-Diphenylbut-2-en-1-one (0.22 g, 1.0 mmol) was employed instead of (*E*)-4,4,4-trifluoro-1,3-diphenylbut-2-en-1-ol and chromatography on silica gel using a mixture of Hex:AcOEt = 4:1 as an eluent afforded the title compound 0.19 g of **(*E*)-6j** (0.84 mmol) in 84% yield as a colorless oil. Rf = 0.2 (*n*-Hex:AcOEt = 6:1). ^{1}H NMR: δ 1.94 (1H, s), 2.21 (3H, d, J = 0.9 Hz), 5.65 (1H, d, J = 8.4 Hz), 6.01 (1H, dq, J = 8.4, 1.4 Hz), 7.22-7.47 (10H, m). ^{13}C NMR: δ 16.2, 70.8, 125.8, 125.9, 127.2, 127.3, 128.1, 128.4, 130.0, 136.7, 142.6, 143.6.

General procedure for the preparation of optically active allylic alcohols [23,24]**:**
(*R*,*E*)- 4,4,4-Trifluoro-1,3-diphenylbut-2-en-1-ol ((*R*,*E*)-6a) [7,8,17,19]

To a flame-dried 30 mL two-necked flask under argon were added (*S*)-2-diphenyl-(pyrrolidin-2-yl)methanol (0.051 g, 0.20 mmol), phenylboronic acid (0.024 g, 0.20 mmol), and toluene (4 mL) and the mixture was refluxed for 4 h. After cooling to room temperature, (*E*)-4,4,4-trifluoro-1,3-diphenylbut-2-en-1-one (0.55 g, 2.0 mmol) was added where 2.2 mL of BH_3 (1 M in THF, 2.2 mmol) was dropped slowly and the whole mixture was stirred for 15 min at room temperature. After quenched the

reaction with MeOH (2.0 mL), the mixture was extracted with Et_2O three times, and the combined Et_2O layer was successively washed with 1 *M* HCl, $NaHCO_3$ aq, and brine. After dried over anhydrous Na_2SO_4, evaporation of the volatiles and purification of the crude materials by silica gel column chromatography using a mixture of Hex:AcOEt = 6:1 as an eluent to obtain 0.52 g (1.9 mmol) of title compound **(*R*,*E*)-6a** in 93% yield as a colorless oil. The isolated product **(*R*,*E*)-6a** was further analyzed by HPLC possessing a CHIRALPAK OD column using a mixture of Hex:*i*-PrOH = 95:5 as an eluent with the flow rate of 0.500 mL/min to afford two peaks at 14.8 (major, (*R*)-isomer) and 17.4 (minor) min whose integration ratio allowed us to calculate the enantiomeric ratio as 86% *ee*. $[\alpha]_D^{25}$ –190.3° (*c* 1.00, $CHCl_3$), and the other physical properties were identical to the ones of racemic form **(*E*)-6a**.

(*R*,*E*)-4,4,4-Trifluoro-3-(4-methoxyphenyl)-1-phenylbut-2-en-1-ol ((*R*,*E*)-6b) [7,20,21]

(*E*)-4,4,4-Trifluoro-3-(4-methoxyphenyl)-1-phenylbut-2-en-1-one (0.61 g, 2.0 mmol) was employed instead of (*E*)-4,4,4-trifluoro-1,3-diphenylbut-2-en-1-ol, and chromatographic purification using a mixture of Hex:AcOEt = 6:1 as an eluent afforded 0.58 g of the desired compound **(*R*,*E*)-6b** (1.9 mmol, 94% yield) as a colorless oil. HPLA analysis by the CHIRALPAK OD column with a mixture of Hex:iPrOH = 95:5 as an eluent with the flow rate of 0.500 mL/min allowed us to observe two peaks at 17.9 (major, (*R*)-isomer) and 23.9 (minor) min whose integration ratio allowed us to calculate the enantiomeric ratio as 84% *ee*. $[\alpha]_D^{25}$ –197.3° (*c* 1.00, $CHCl_3$), and the other physical properties were identical to the ones of racemic form **(*E*)-6b**.

(*R*,*E*)-4,4,4-Trifluoro-3-(4-fluorophenyl)-1-phenylbut-2-en-1-ol ((*E*)-6c) [20,21]

(*E*)-4,4,4,-Trifluoro-3-(4-fluorophenyl)-1-phenylbut-2-en-1-one (0.29 g, 1.0 mmol) was employed instead of (*E*)-4,4,4-trifluoro-1,3-diphenylbut-2-en-1-ol, and chromatographic purification using a mixture of Hex:AcOEt = 6:1 as an eluent afforded 0.29 g of the desired compound **(*R*,*E*)-6c** (0.99 mmol, 97% yield) as a colorless oil. HPLA analysis by the CHIRALPAK OD column with a mixture of Hex:iPrOH = 95:5 as an eluent with the flow rate of 0.500 mL/min allowed us to observe two peaks at 14.9 (major, (*R*)-isomer) and 17.3 (minor) min whose integration ratio allowed us to calculate the enantiomeric ratio as 80% *ee*. $[\alpha]_D^{25}$ –176.9° (*c* 1.00, $CHCl_3$), and the other physical properties were identical to the ones of racemic form **(*E*)-6c**.

General procedure for the isomerization of racemic allylic alcohols (*E*)-6 to the corresponding saturated ketones 7: 4,4,4-Trifluoro-1,3-diphenylbutan-1-one (7a) [19,25,26,27]

To a flame-dried 30 mL round-bottomed flask were added under argon 0.14 g of (*E*)-4,4,4-trifluoro-1,3-diphenylbut-2-en-1-ol **(*E*)-6a** (0.50 mmol), 0.039 g of DBU (0.25 mml), and 5.0 mL of toluene and the whole mixture was refluxed for 3 h. The mixture was extracted with AcOEt three times and the combined organic phase was dried over anhydrous Na_2SO_4. Filtration and concentration afforded crude materials which was chromatographed on silica gel using a mixture of Hex:AcOEt = 6:1 as an eluent to give 0.13 g of the pure title compound **7a** (0.46 mmol, 91% yield) as a white solid. Rf = 0.43 (Hex:AcOEt = 10:1). ^{1}H NMR: δ 3.60 (1H, dd, *J* = 17.7, 4.2 Hz), 3.71 (1H, dd, *J* = 17.9, 8.9 Hz), 4.25 (1H, m), 7.28-7.41 (5H, m), 7.43-7.49 (2H, m), 7.55-7.61 (1H, m), 7.91-7.94 (2H, m). ^{13}C NMR: δ 38.2 (d, *J* = 1.2 Hz), 44.7 (q, *J* = 27.5 Hz), 126.9 (q, *J* = 278.9 Hz), 128.0, 128.2, 128.6, 129.0, 133.5, 134.5 (q, *J* = 1.9 Hz), 136.1, 195.2. ^{19}F NMR: δ –70.95 (d, *J* = 9.0 Hz).

4,4,4-Trifluoro-3-(4-methoxyphenyl)-1-phenylbutan-1-one (7b) [19,26]

(*E*)-4,4,4-Trifluoro-3-(4-methoxyphenyl)-1-phenylbut-2-en-1-ol **(*E*)-6b** (0.15 g, 0.50 mmol) was reacted for 3 h instead of 4,4,4-trifluoro-1,3-diphenylbutan-1-one **(*E*)-6a** and chromatographic purification using a mixture of Hex:AcOEt = 6:1 as an eluent afforded 0.14 g of the desired product (0.45 mmol, 89% yield) as a white solid. Rf = 0.57 (Hex:AcOEt = 6:1). ^{1}H NMR: δ 3.56 (1H, dd, *J* = 17.7, 4.2 Hz), 3.67 (1H, dd, *J* = 18.0, 9.0 Hz), 3.78 (3H, s), 4.12-4.26 (1H, m), 6.84-6.89 (2H, m), 7.25-7.32 (2H, m), 7.43-7.49 (2H, m), 7.55-7.61 (1H, m), 7.91-7.94 (2H, m). ^{13}C NMR: δ 38.2, 43.9 (q, *J* = 27.3 Hz), 55.0, 114.0, 126.4, 127.0 (q, *J* = 278.5 Hz), 128.0, 128.6, 130.0, 133.4, 136.2, 159.3, 195.3. ^{19}F NMR: δ –71.35 (d, *J* = 9.0 Hz).

4,4,4-Trifluoro-3-(4-fluorophenyl)-1-phenylbutan-1-one (7c) [26,28]

(*E*)-4,4,4-Trifluoro-3-(4-fluorophenyl)-1-phenylbut-2-en-1-ol **(*E*)-6c** (0.15 g, 5.0 mmol) was reacted for 2 h instead of 4,4,4-trifluoro-1,3-diphenylbutan-1-one **(*E*)-6a** and chromatographic purification using a mixture of Hex:AcOEt = 6:1 as an eluent afforded 0.15 g of the desired product (0.50 mmol, quantitative yield) as a white solid. Rf = 0.43 (Hex:AcOEt = 6:1). ^{1}H NMR: δ 3.54-3.72 (2H, m), 4.16-4.30 (1H, m), 7.03 (2H, t, *J* = 8.7 Hz), 7.35-7.61 (5H, m), 7.92 (2H, d, *J* = 7.2 Hz). ^{13}C NMR: δ 38.3 (q, *J* = 1.3 Hz), 44.1 (q, *J* = 27.3 Hz), 115.6 (d, *J* = 21.7 Hz), 126.8 (qd, *J* = 279.1, 1.3 Hz), 128.0, 128.7, 130.3 (dq, *J* = 3.8, 1.9 Hz), 130.7 (d, *J* = 8.1 Hz), 133.6, 136.2, 162.6 (d, *J*

= 246.9 Hz), 195.1. ^{19}F NMR: δ –71.17 (dd, *J* = 9.0, 4.5 Hz), –114.97 to –115.08 (m).

1-Phenyl-3-(trifluoromethyl)pentan-1-one (7d) [29,30]

(*E*)-1-Phenyl-3-(trifluoromethyl)pent-2-en-1-ol **(*E*)-6d** (0.12 g, 0.51 mmol) was reacted for 24 h instead of 4,4,4-trifluoro-1,3-diphenylbutan-1-one **(*E*)-6a** and chromatographic purification using a mixture of Hex:AcOEt = 10:1 as an eluent afforded 0.092 g of the desired product (0.40 mmol, 78% yield) as a colorless oil. Rf = 0.56 (Hex:AcOEt = 10:1). ^{1}H NMR: δ 1.00 (3H, t, *J* = 7.5 Hz), 1.46-1.60 (1H, m), 1.70-1.84 (1H, m), 2.93-3.31 (2H, m), 3.22-3.31 (1H, m), 7.47-7.52 (2H, m), 7.58-7.64 (1H, m), 7.96-7.99 (2H, m). ^{13}C NMR: δ 11.3, 21.7 (q, *J* = 2.5 Hz), 36.7 (q, *J* = 2.3 Hz), 39.3 (q, *J* = 25.6 Hz), 128.0, 128.3 (q, *J* = 279.1 Hz), 128.7, 133.5, 136.4, 196.5. ^{19}F NMR: δ –71.94 (d, *J* = 9.0 Hz). IR (neat): ν 3742, 2940, 1691, 1598, 1450, 1257, 1171, 957, 753, 715, 691. HRMS (FAB+, *m/z*): $[M+H]^+$ calcd for $C_{12}H_{14}F_3O$, 231.0997; Found, 231.1038.

1,5-Diphenyl-3-(trifluoromethyl)pentan-1-one (7e)

(*E*)-1,5-Diphenyl-3-(trifluoromethyl)pent-2-en-1-ol **(*E*)-6e** (0.15 g, 5.0 mmol) was reacted for 24 h instead of 4,4,4-trifluoro-1,3-diphenylbutan-1-one **(*E*)-6a** and chromatographic purification using a mixture of Hex:AcOEt = 10:1 as an eluent afforded 0.14 g of the desired product (0.46 mmol, 93% yield) as a colorless oil. Rf = 0.47 (Hex:AcOEt = 10:1). ^{1}H NMR: δ 1.72-1.84 (1H, m), 1.97-2.10 (1H, m), 2.65-2.81 (2H, m), 3.05-3.38 (3H, m), 7.16-7.21 (3H, m), 7.25-7.30 (2H, m), 7.46-7.52 (2H, m), 7.58-7.63 (1H, m), 7.95-7.99 (2H, m). ^{13}C NMR: δ 30.7 (q, *J* = 2.1 Hz), 33.2, 37.2 (q, *J* = 2.3 Hz), 38.0 (q, *J* = 26.3 Hz), 126.1, 128.0, 128.2 (q, *J* = 278.9 Hz), 128.2, 128.4, 128.7, 133.5, 136.2, 141.0, 196.2. ^{19}F NMR: δ –71.90 (d, *J* = 9.0 Hz). IR (neat): ν 3063, 3029, 29 37, 1690, 1598, 1581, 1497, 1450, 1113, 1002, 754, 691. HRMS (ESI+, m/z): $[M+Na]^+$ calcd for $C_{18}H_{17}F_3NaO$, 329.1129; Found, 329.1124.

1-(4-Methoxyphenyl)-5-phenyl-3-(trifluoromethyl)pentan-1-one (7f)

(*E*)-1-(4-Methoxyphenyl)-5-phenyl-3-(trifluoromethyl)pent-2-en-1-ol **(*E*)-6f** (0.17 g, 5.0 mmol) was reacted for 48 h instead of 4,4,4-trifluoro-1,3-diphenylbutan-1-one **(*E*)-6a** and chromatographic purification using a mixture of Hex:AcOEt = 6:1 as an eluent afforded 0.13 g of the desired product (0.38 mmol, 76% yield) as a yellow oil. Rf = 0.26 (Hex:AcOEt = 6:1). ^{1}H NMR: δ 1.71-1.83 (1H, m), 1.96-2.08 (1H, m), 2.64-2.81 (2H, m), 3.04 (1H, dd, *J* = 17.0, 7.7 Hz), 3.11-3.20 (1H, m), 3.27 (1H, dd, *J* = 16.7, 3.5 Hz), 3.89 (3H, s), 6.91-6.97 (2H, m), 7.15-7.30 (5H, m), 7.92-7.97 (2H, m).

^{13}C NMR: δ 30.8 (q, *J* = 1.8 Hz), 33.2, 36.8 (q, *J* = 1.9 Hz), 38.1 (q, *J* = 26.0 Hz), 55.5, 113.8, 126.1, 128.3, 128.3 (q, *J* = 279.1 Hz), 128.4, 129.4, 130.3, 141.1, 163.8, 194.7. ^{19}F NMR: δ –71.86 (d, *J* = 9.3 Hz). IR (neat): ν 3749, 3733, 1683, 1602, 1576, 1510, 1263, 983, 938, 833, 762. Calcd for $C_{19}H_{19}F_3O_2$: C, 67.85; H, 5.69. Found: C, 68.11; H, 6.03.

1-(4-Bromophenyl)-5-phenyl-3-(trifluoromethyl)pentan-1-one (7g)

(*E*)-1-(4-Bromophenyl)-3-trifluoromethyl-5-phenylpent-2-en-1-ol **(*E*)-6g** (0.19 g, 0.50 mmol) was reacted for 3 h instead of 4,4,4-trifluoro-1,3-diphenylbutan-1-one **(*E*)-6a** and chromatographic purification using a mixture of Hex:AcOEt = 6:1 as an eluent afforded 0.18 g of the desired product (0.46 mmol, 91% yield) as a colorless oil. Rf = 0.62 (Hex:AcOEt = 6:1). ^{1}H NMR: δ 1.76-1.81 (1H, m), 2.00-2.01 (1H, m), 2.69-2.76 (2H, m), 3.02 (1H, dd, *J* = 17.4, 7.2 Hz), 3.06-3.18 (1H, m), 3.27 (1H, dd, *J* = 17.1, 3.6 Hz), 7.15-7.30 (5H, m), 7.60-7.64 (2H, m), 7.78-7.83 (2H, m). ^{13}C NMR: δ 30.5 (q, *J* = 2.1 Hz), 33.1, 37.2 (q, *J* = 2.3 Hz), 38.0 (q, *J* = 26.3 Hz), 126.1, 128.1 (q, *J* = 279.2 Hz), 128.2, 128.4, 128.7, 129.5, 132.0, 134.9, 140.8, 195.2. ^{19}F NMR: δ –71.86 (d, *J* = 9.3 Hz). IR (neat): ν 3029, 2934, 1692, 1586, 1397, 1264, 1151, 1112, 1010, 813, 753. Calcd for $C_{18}H_{16}BrF_3O$: C, 56.12; H, 4.19. Found: C, 56.45; H, 4.61.

(*R*)-4,4,4-Trifluoro-1,3-diphenylbutan-1-one ((*R*)-7a) [6,7,16,17,20,31]

(*R*,*E*)-4,4,4-Trifluoro-1,3-diphenylbut-2-en-1-ol **(*R*,*E*)-6a** (0.25 g, 0.91 mmol, 83% *ee*) was reacted for 3 h instead of its racemate **(*E*)-6a** and the same reaction procedure afforded 0.22 g of the title compound **(*R*)-7a** (0.80 mmol, 89% yield) as a white solid. The isolated product **(*R*)-7a** was further analyzed by HPLC possessing a CHIRALPAK AD column using a mixture of Hex:iPrOH = 300:1 as an eluent with the flow rate of 1.00 mL/min to afford two peaks at 12.8 (minor) and 15.6 (major, (*R*)-isomer) min whose integration ratio allowed us to calculate the enantiomeric ratio as 85% *ee*. $[\alpha]_D^{20}$ +22.0° (c 1.02, CCl_4), and the other physical properties were identical to the ones of the corresponding racemic compound.

(*R*)-4,4,4-Trifluoro-3-(4-methoxyphenyl)-1-phenylbutan-1-one (7b) [7,20,31]

(*R*,*E*)-4,4,4-Trifluoro-3-(4-methoxyphenyl)-1-phenylbut-2-en-1-ol **(*R*,*E*)-6b** (0.27 g, 0.87 mmol, 80% *ee*) was used instead of (*E*)-4,4,4-Trifluoro-1,3-diphenylbut-2-en-1-ol **(*E*)-6a** and the same reaction procedure afforded 0.23 g of the title compound **(*R*)-7b** (0.74 mmol, 85% yield) as a white solid. The isolated product **(*R*)-7b** was further analyzed by HPLC possessing a CHIRALPAK AD column using a mixture of

Hex:*i*-PrOH = 100:1 as an eluent with the flow rate of 1.00 mL/min to afford two peaks at 14.1 (minor) and 20.6 (major, (*R*)-isomer) min whose integration ratio allowed us to calculate the enantiomeric ratio as 77% *ee*. $[\alpha]_D^{20}$ +24.2° (c 1.04, CCl_4), and the other physical properties were identical to the ones of the corresponding racemic compound.

4,4,4-Trifluoro-3-(4-fluorophenyl)-1-phenylbut-2-en-1-ol (7c) [31]

(*R*,*E*)-4,4,4-Trifluoro-3-(4-fluorophenyl)-1-phenylbut-2-en-1-ol **(*R*,*E*)-6c** (0.25 g, 0.86 mmol, 80% *ee*) was used instead of (*E*)-4,4,4-trifluoro-1,3-diphenylbut-2-en-1-ol **(*E*)-6a** and the same reaction procedure afforded 0.22 g of the title compound **(*R*)-7c** (0.76 mmol, 88% yield) as a white solid. The isolated product **(*R*)-7c** was further analyzed by HPLC possessing a CHIRALPAK AD column using a mixture of Hex: *i*-PrOH = 100:1 as an eluent with the flow rate of 1.00 mL/min to afford two peaks at 14.6 (minor) and 20.6 (major, (*R*)-isomer) min whose integration ratio allowed us to calculate the enantiomeric ratio as 78% *ee*. $[\alpha]_D^{20}$ +25.8° (c 1.01, CCl_4), and the other physical properties were identical to the ones of the corresponding racemic compound.

3. ^{1}H and ^{13}C NMR charts

Dimethyl 2-oxo-2-phenylethylphosphonate (9a)

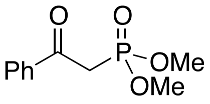

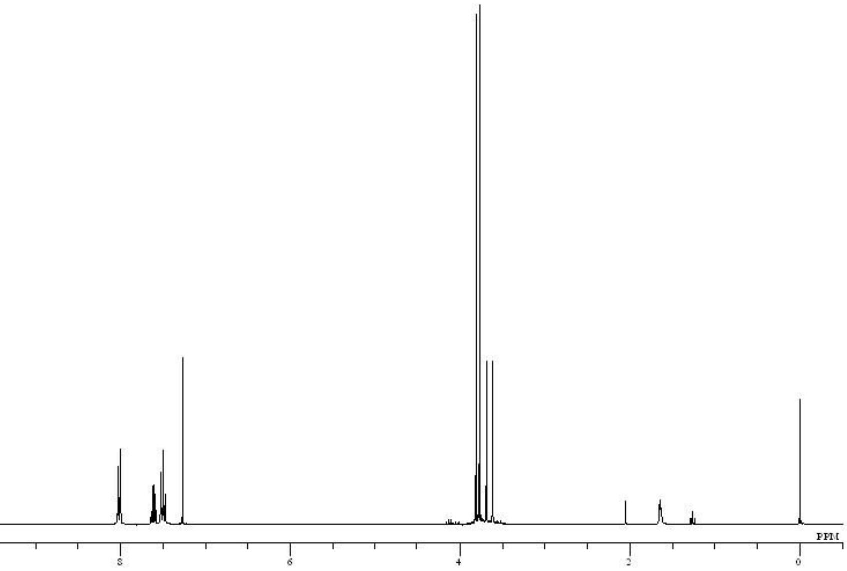

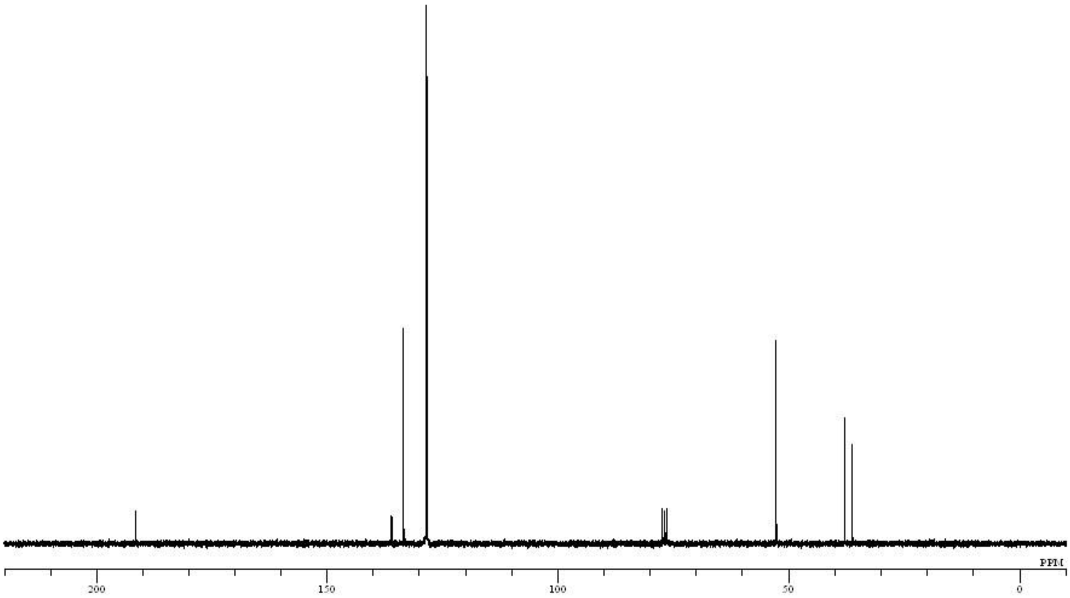

Dimethyl 2-oxo-4-phenylbutylphosphonate (9b)

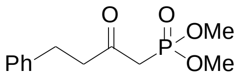

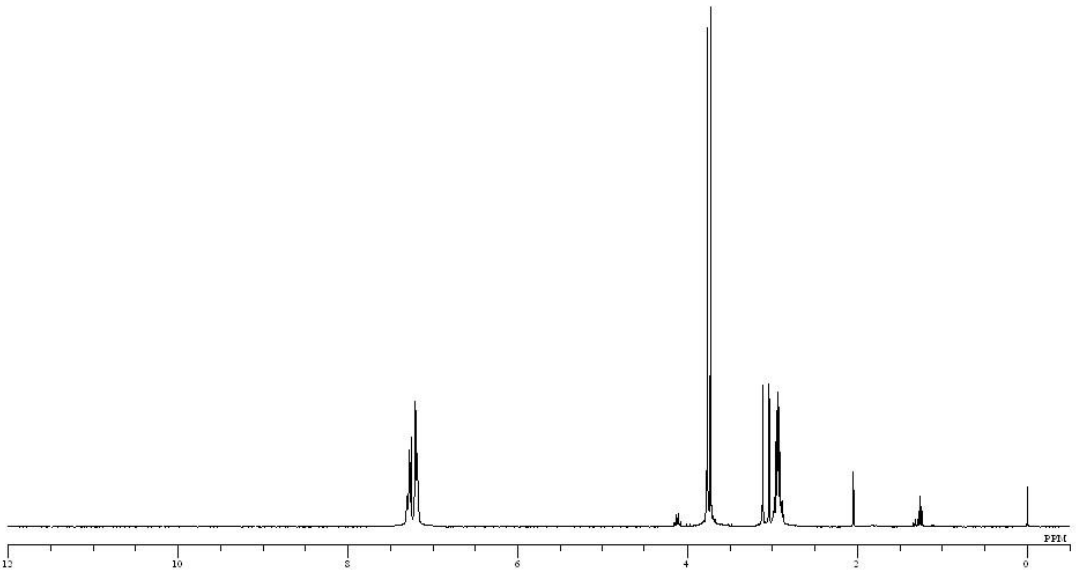

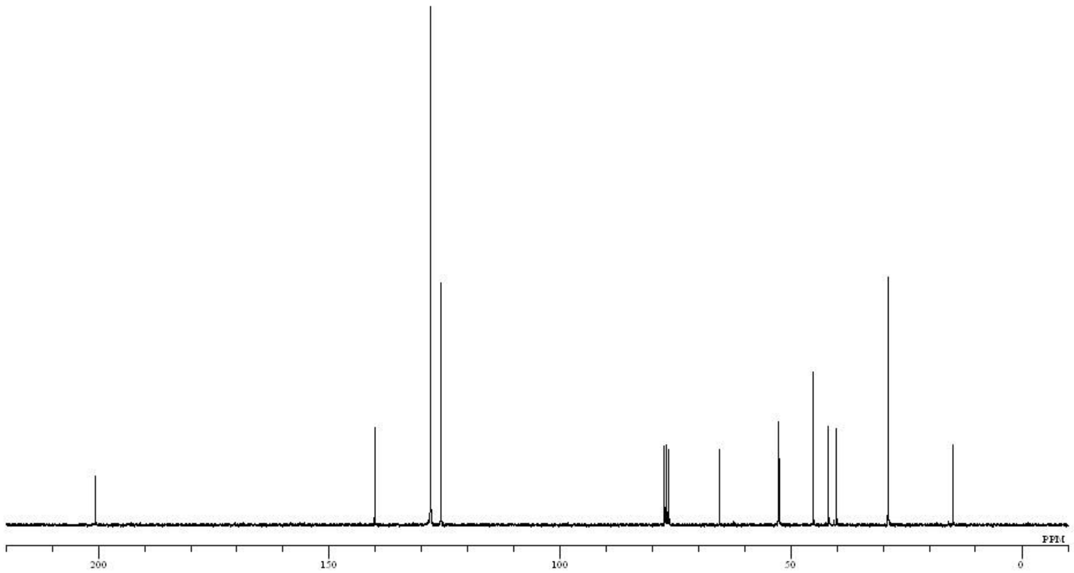

Dimethyl 2-oxobutylphosphonate (9c)

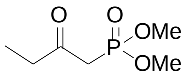

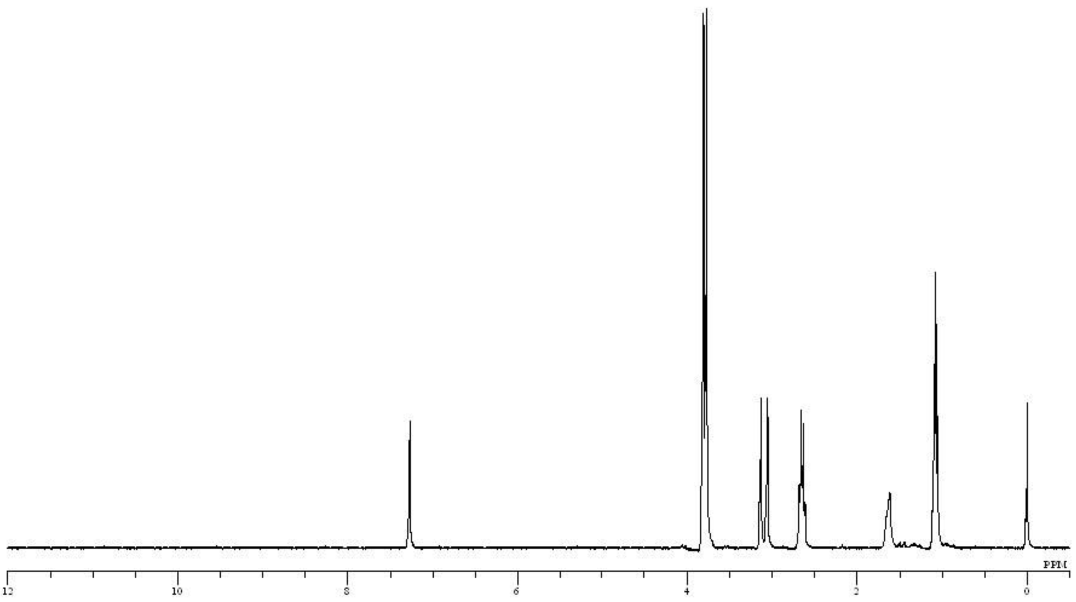

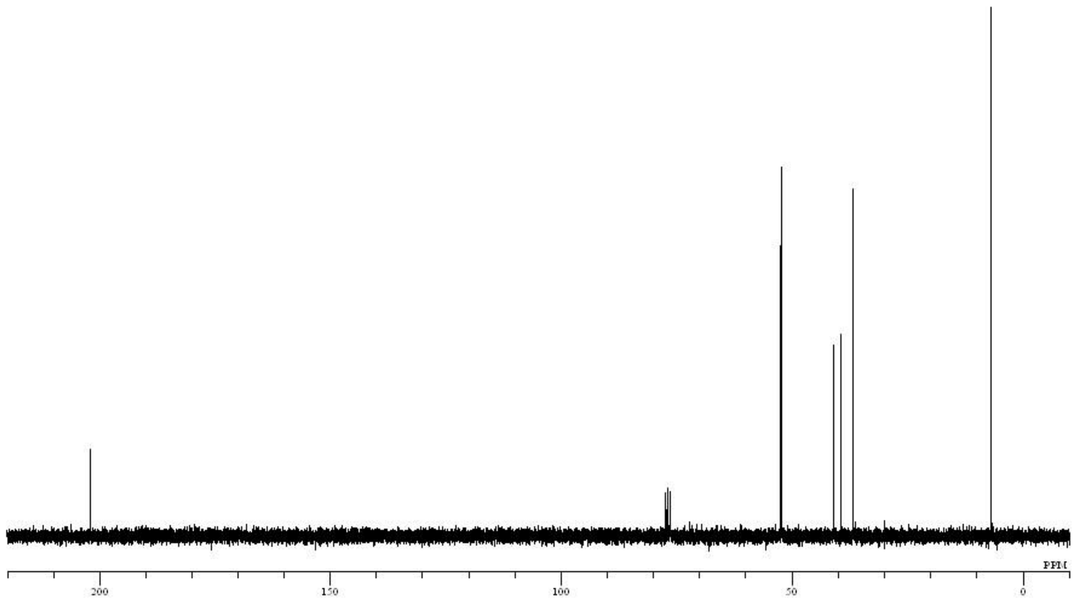

Dimethyl 2-(4-methoxyphenyl)-2-oxoethylphosphonate (9d)

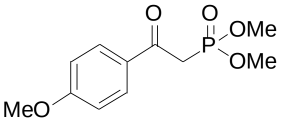

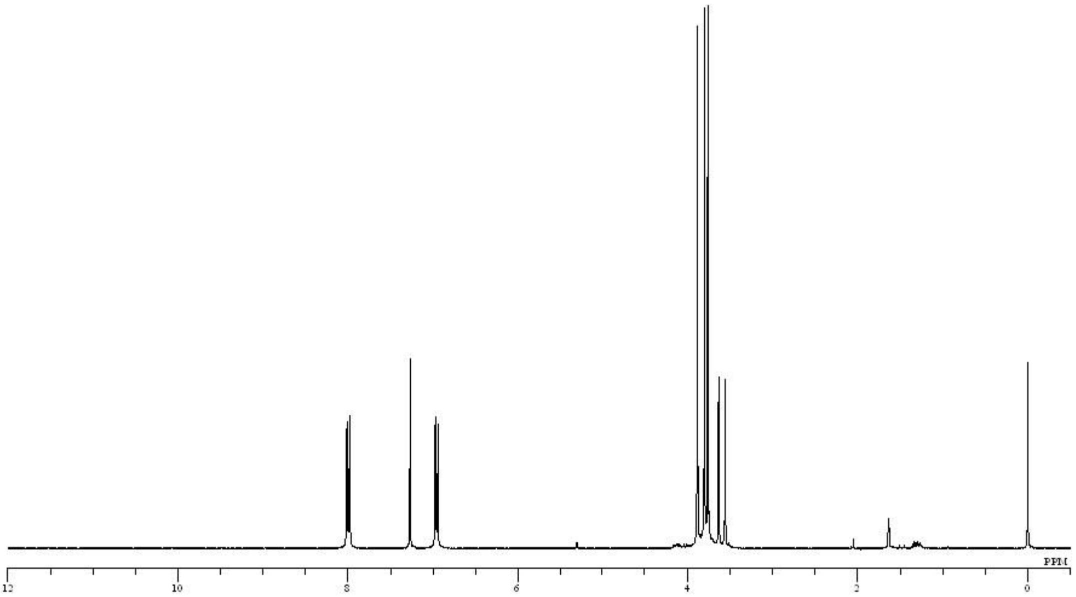

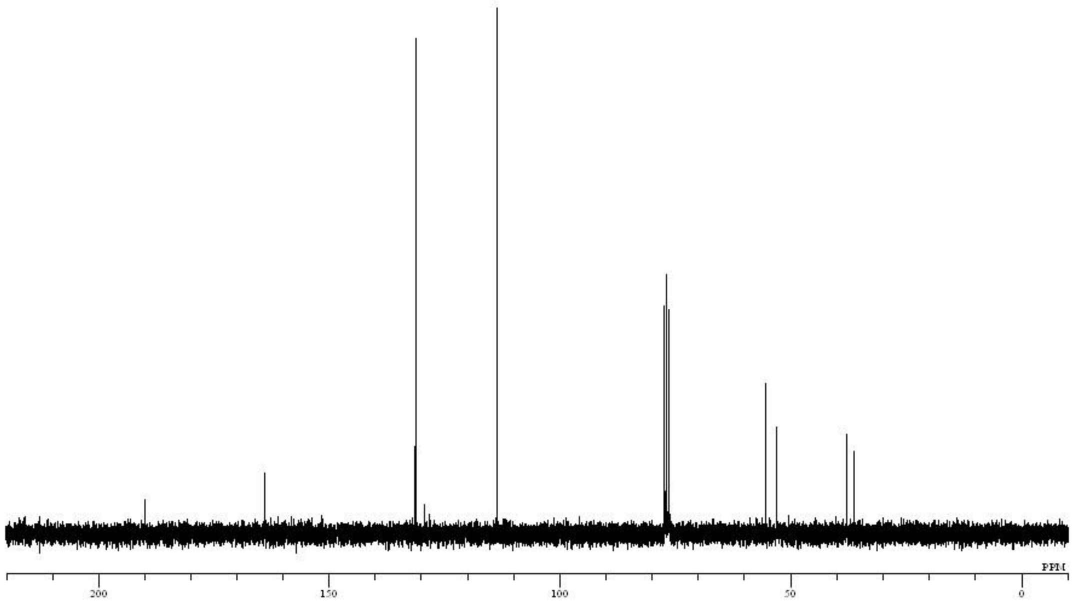

Dimethyl 2-(4-nitrophenyl)-2-oxoethylphosphonate (9e)

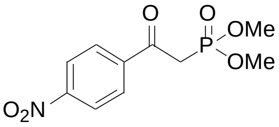

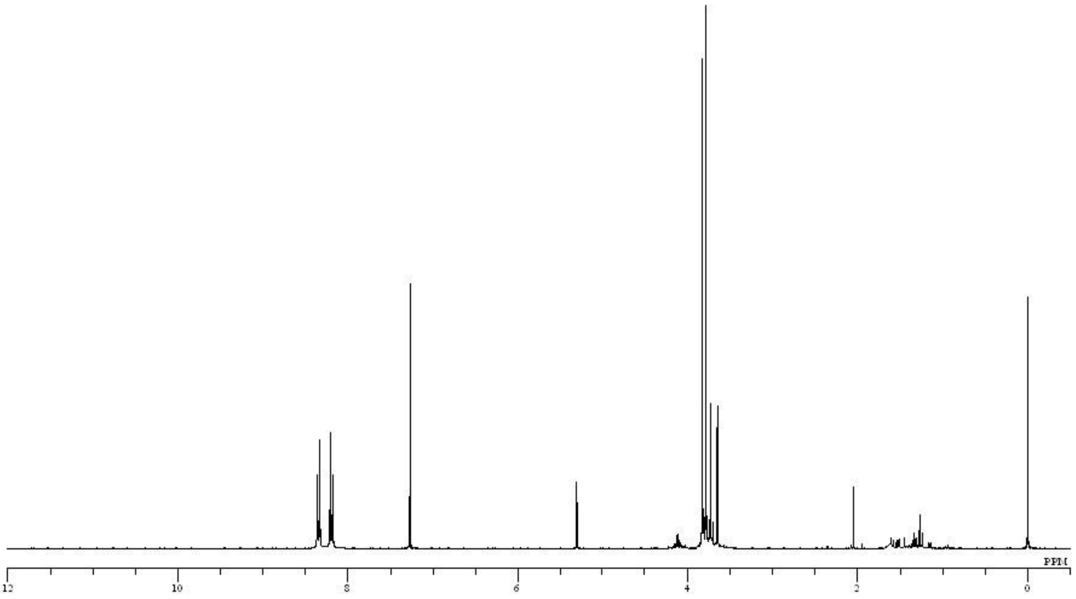

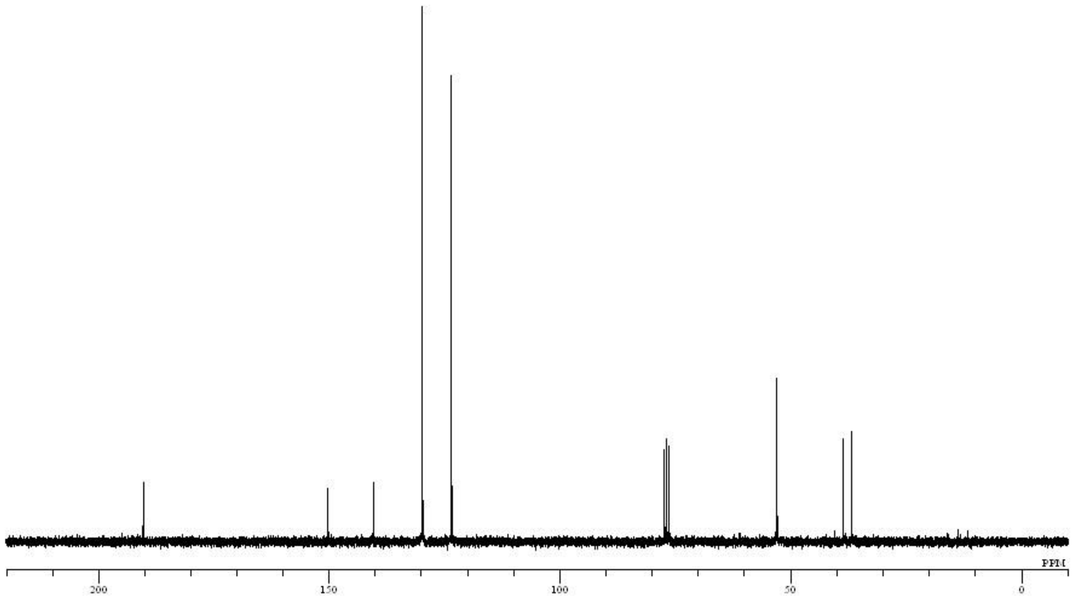

Dimethyl 2-(4-bromophenyl)-2-oxoethylphosphonate (9f)

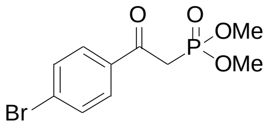

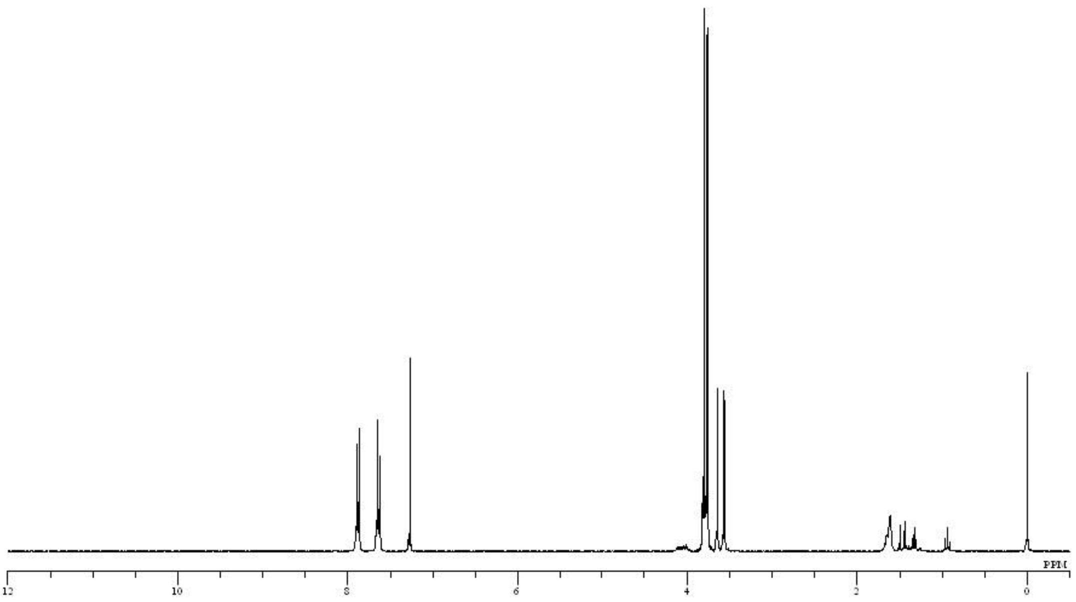

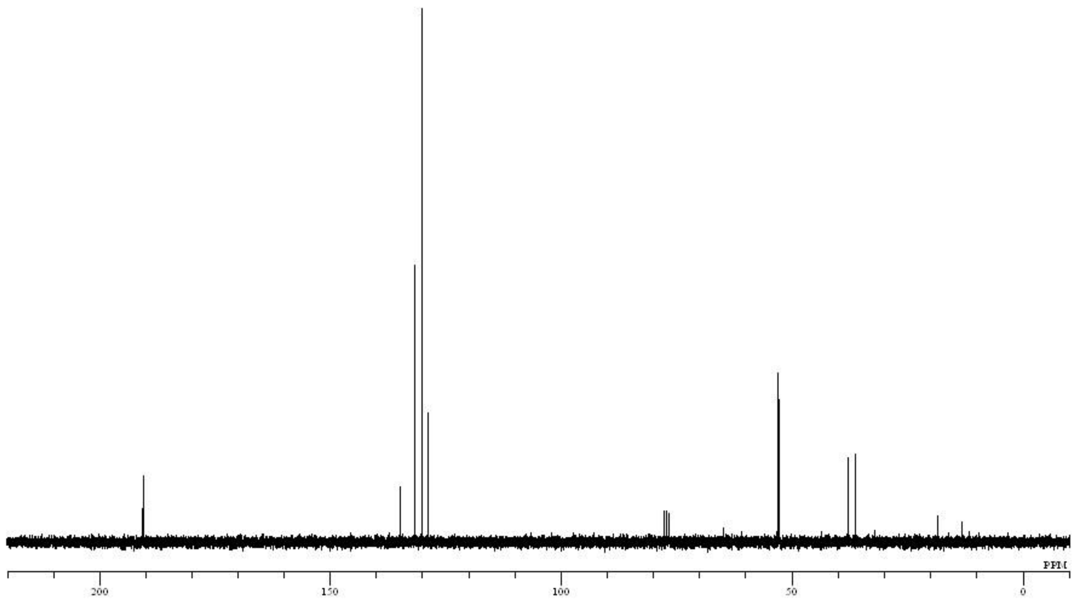

(*E*)-4,4,4-Trifluoro-1,3-diphenylbut-2-en-1-one ((*E*)-10a)

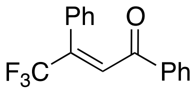

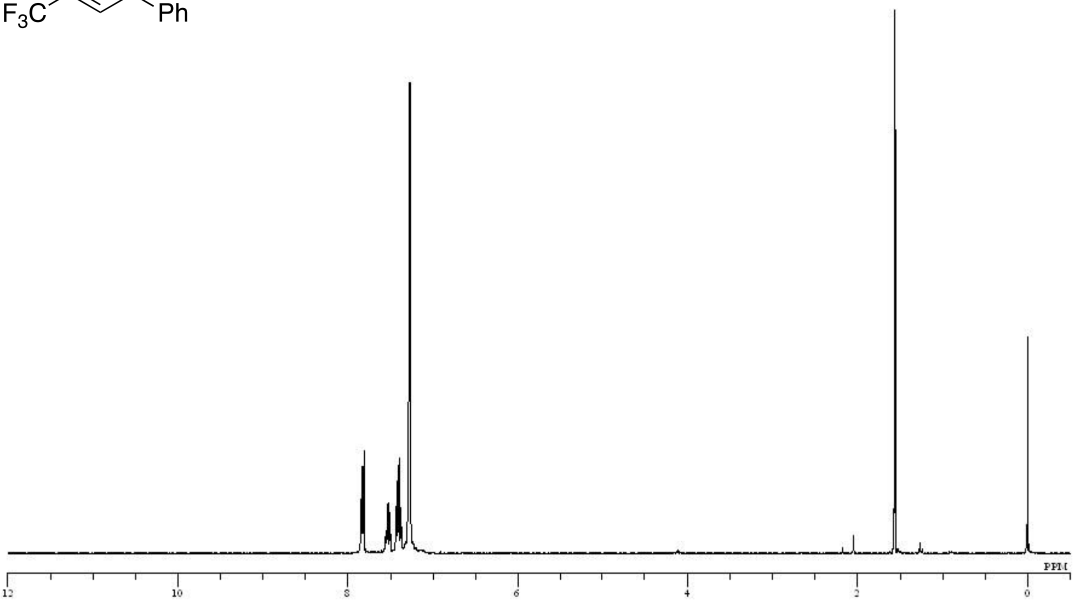

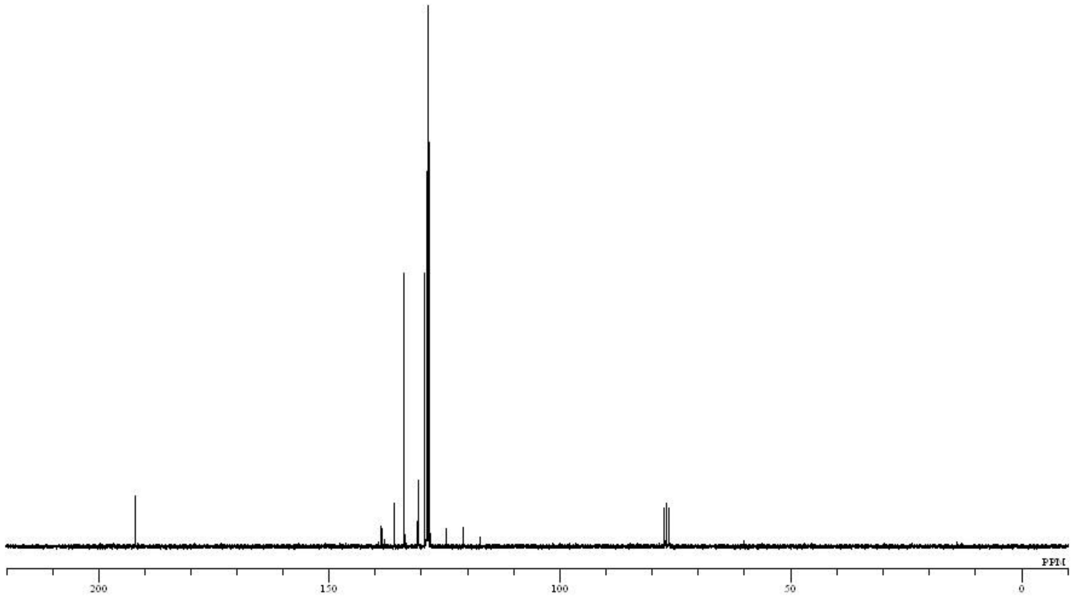

(*E*)-4,4,4-Trifluoro-3-(4-methoxyphenyl)-1-phenylbut-2-en-1-one ((*E*)-10b)

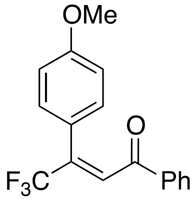

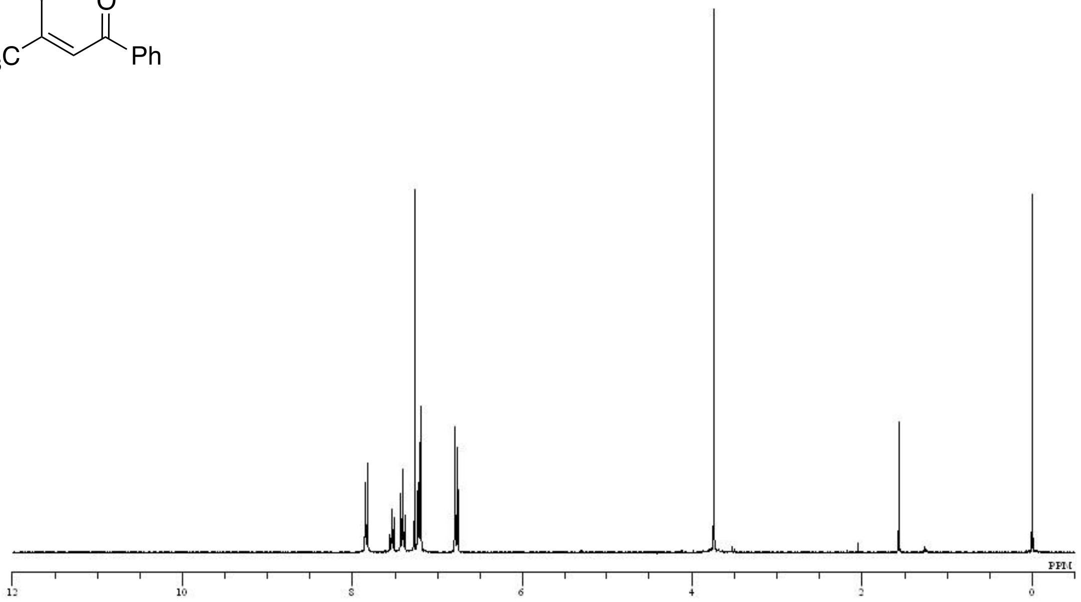

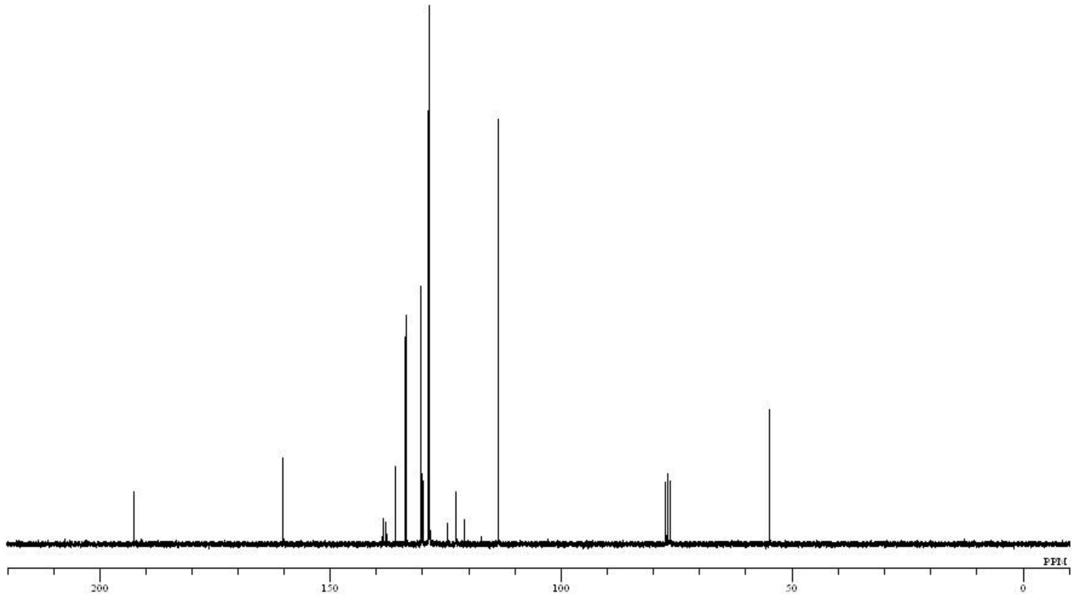

(*Z*)-4,4,4,-Trifluoro-3-(4-methoxyphenyl)-1-phenylbut-2-en-1-one ((*Z*)-10b)

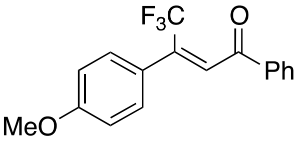

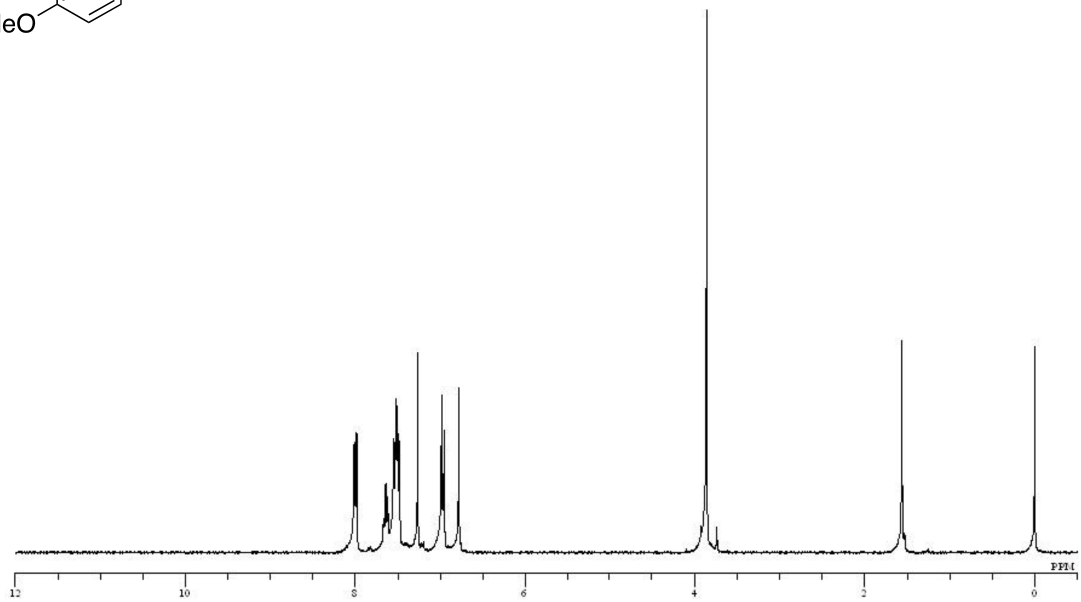

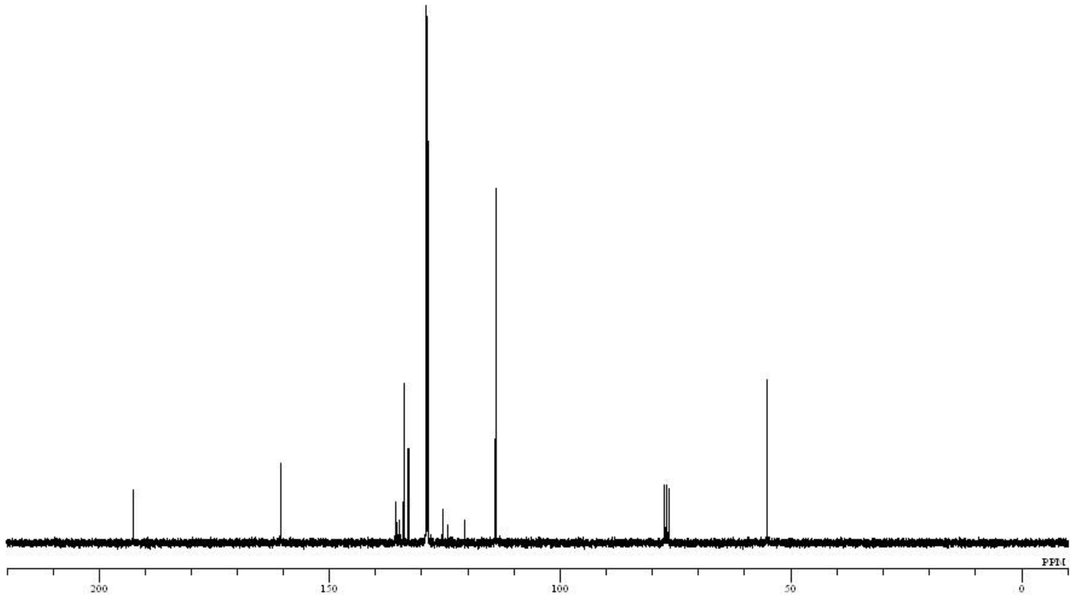

(*E*)-4,4,4-Trifluoro-3-(4-fluorophenyl)-1-phenylbut-2-en-1-one ((*E*)-10c)

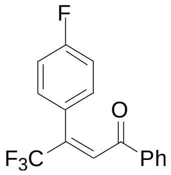

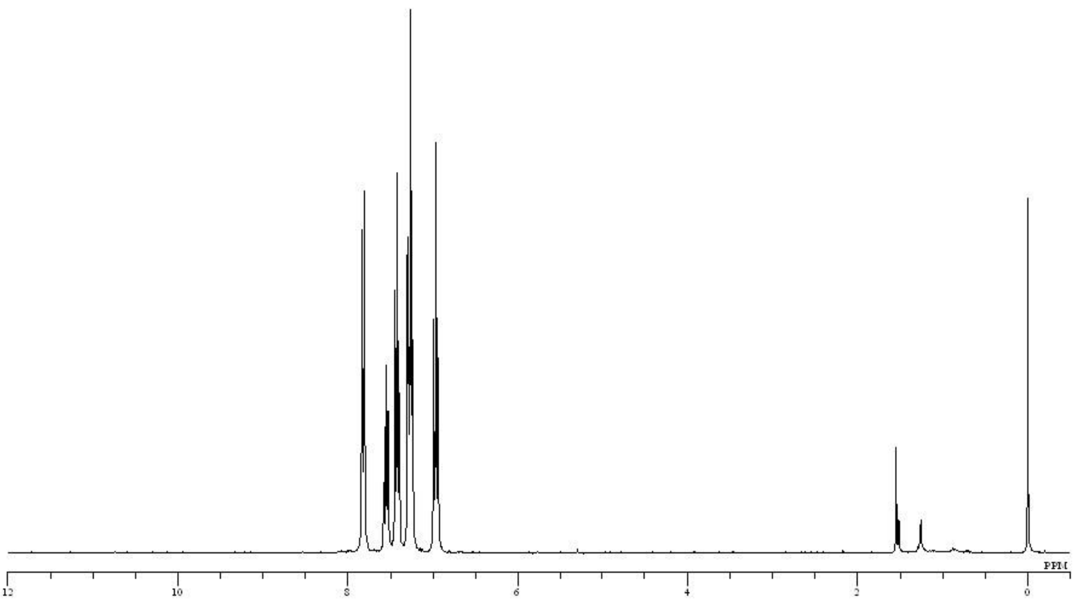

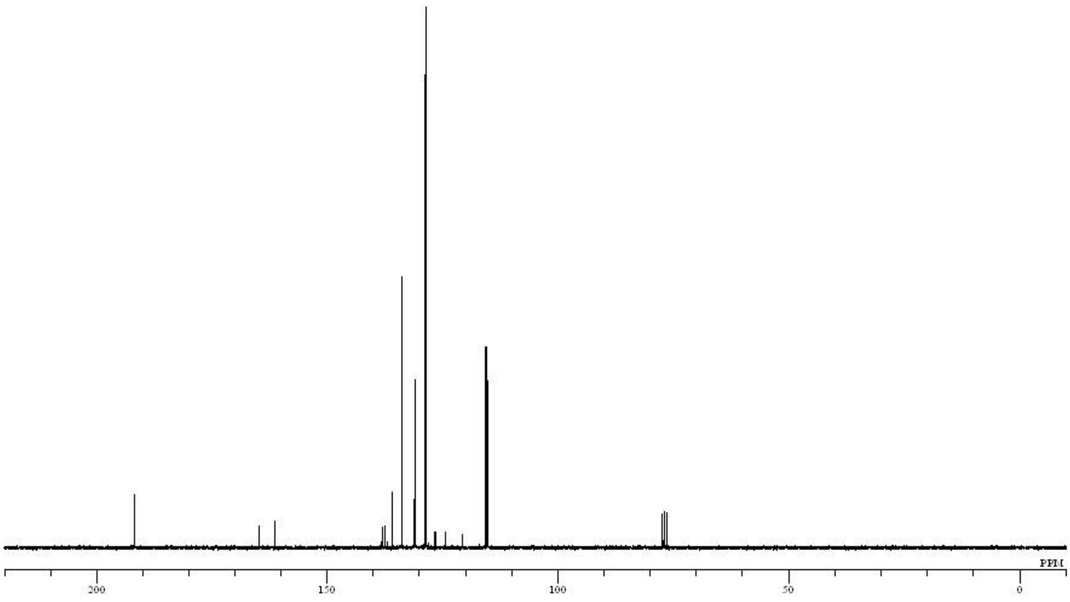

(*E*)-1-Phenyl-3-(trifluoromethyl)pent-2-en-1-one ((*E*)-10d)

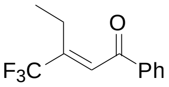

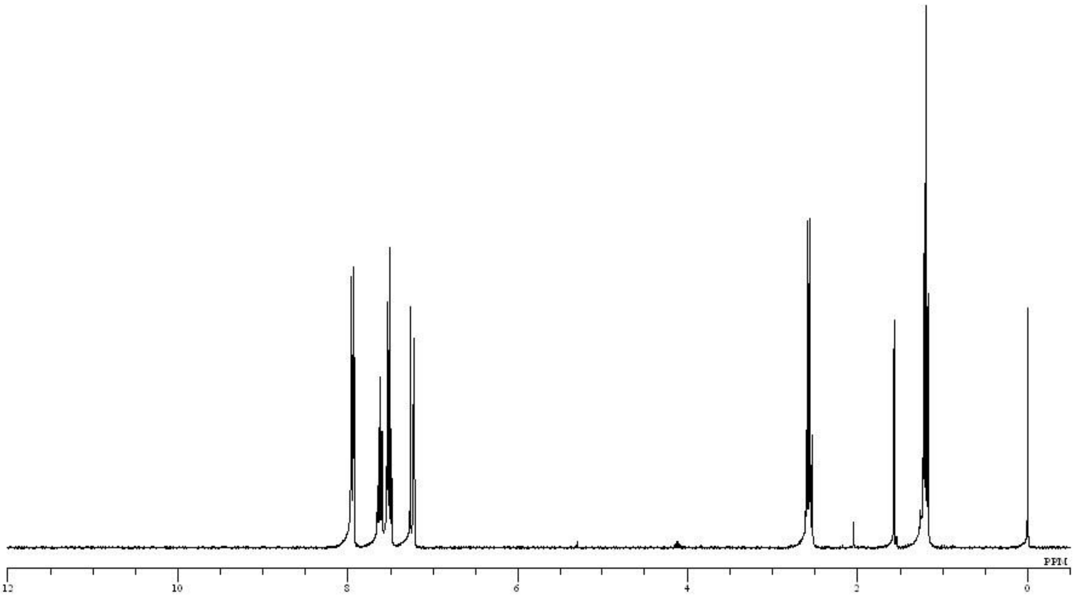

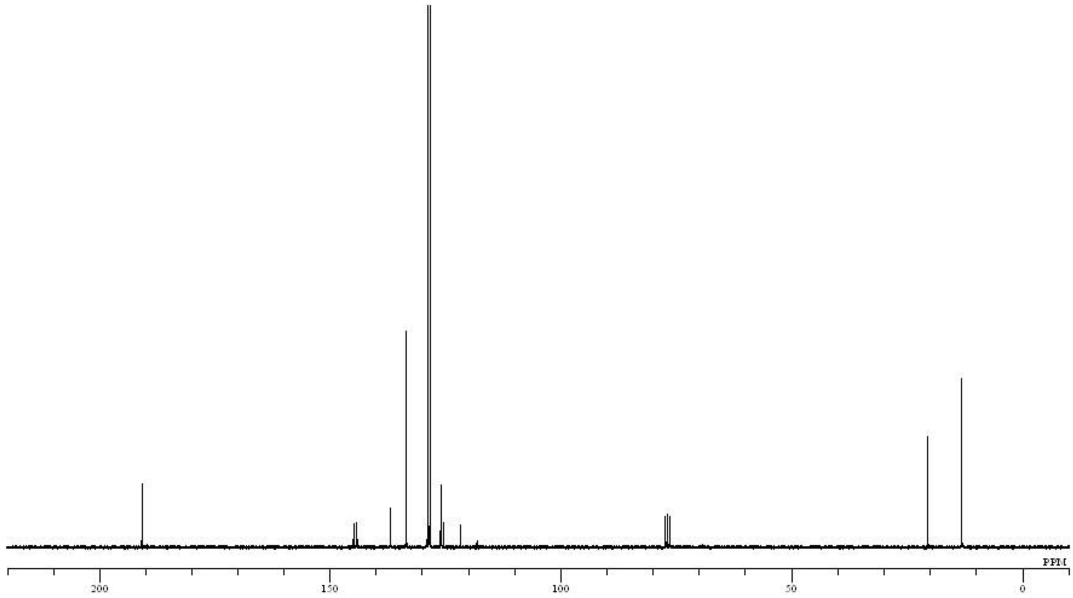

(*E*)-1,5-Diphenyl-3-(trifluoromethyl)pent-2-en-1-one ((*E*)-10e)

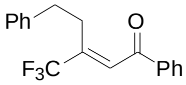

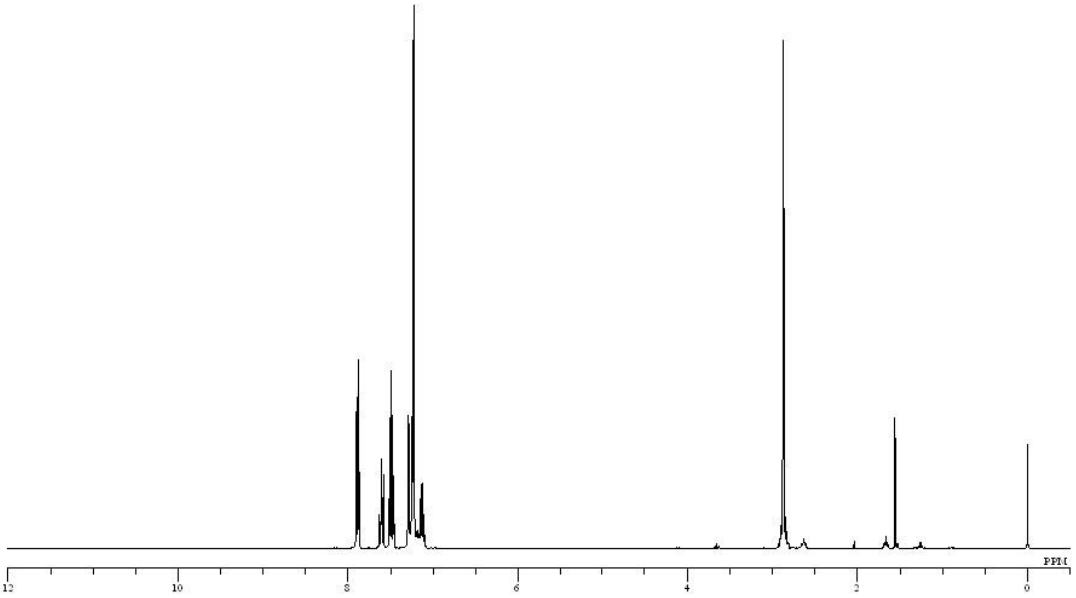

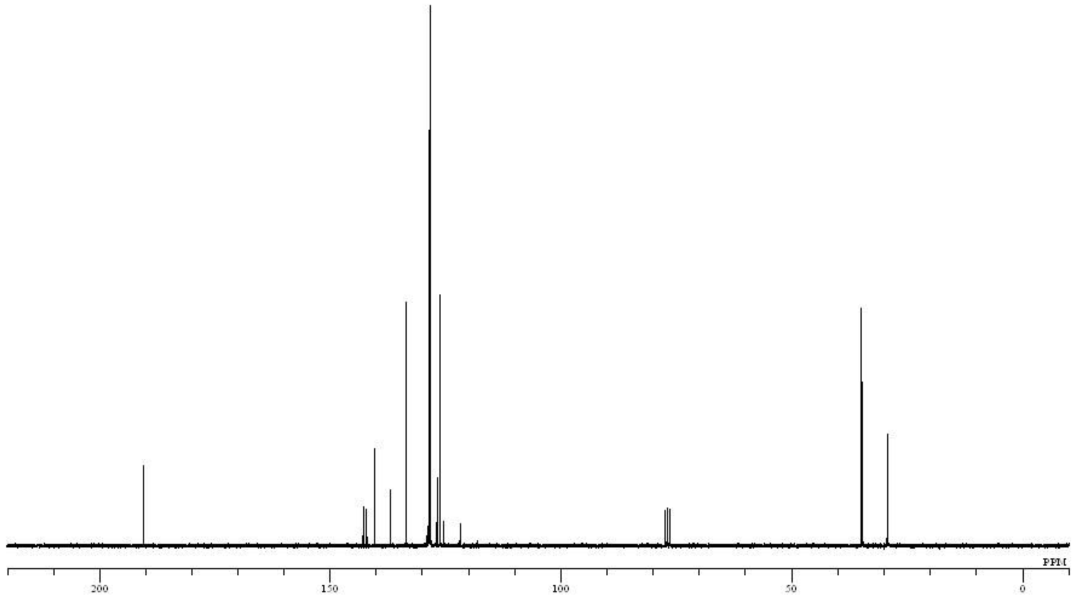

(*E*)-1-(4-Methoxyphenyl)-5-phenyl-3-(trifluoromethyl)pent-2-en-1-one ((*E*)-10f)

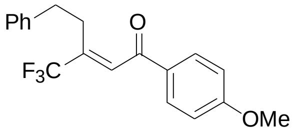

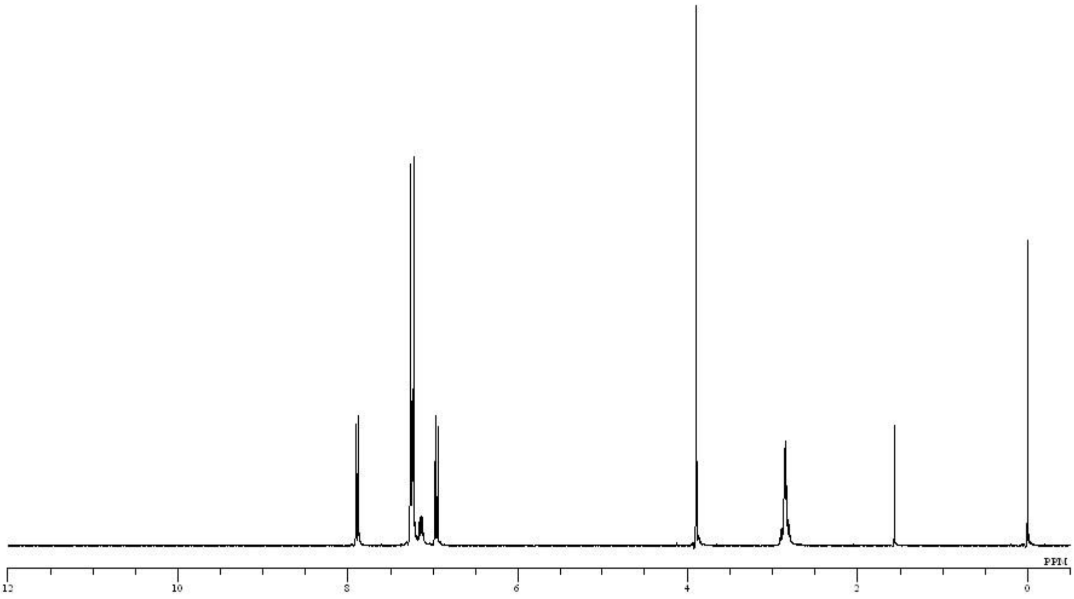

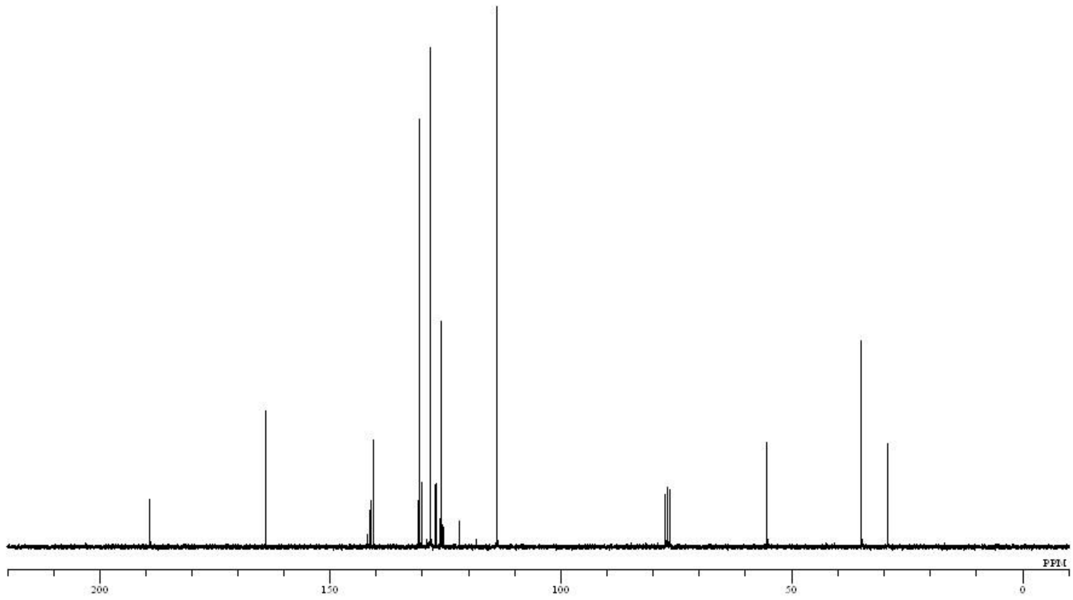

(*E*)-1-(4-Bromophenyl)-5-phenyl-3-(trifluoromethyl)pent-2-en-1-one ((*E*)-10g)

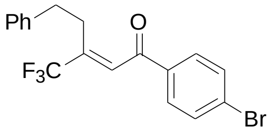

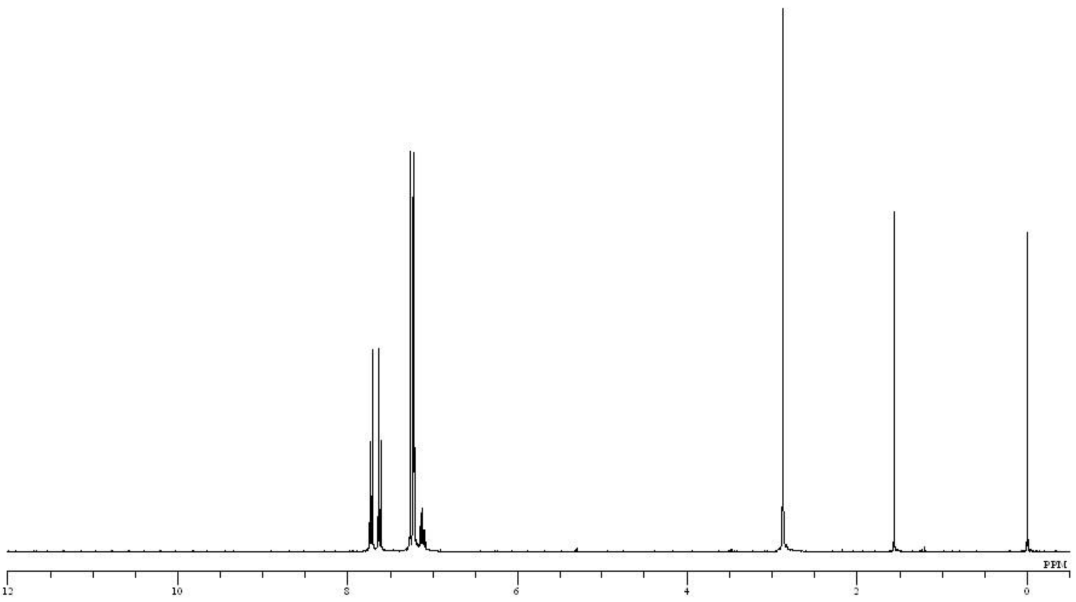

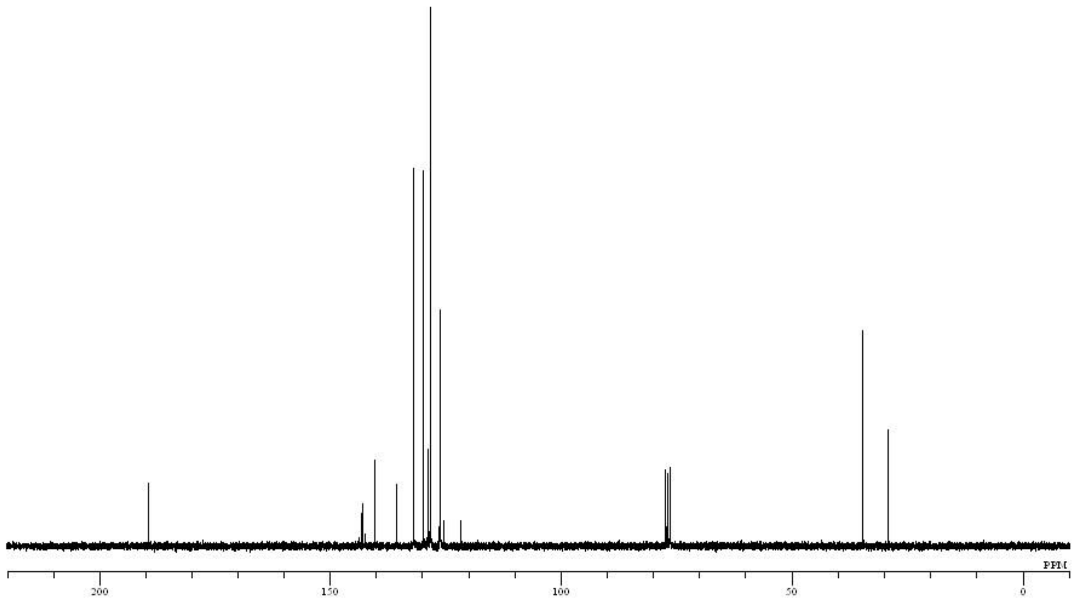

(*E*)-6,6,6-Trifluoro-1,5-diphenylhex-4-en-3-one ((*E*)-10h)

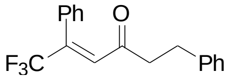

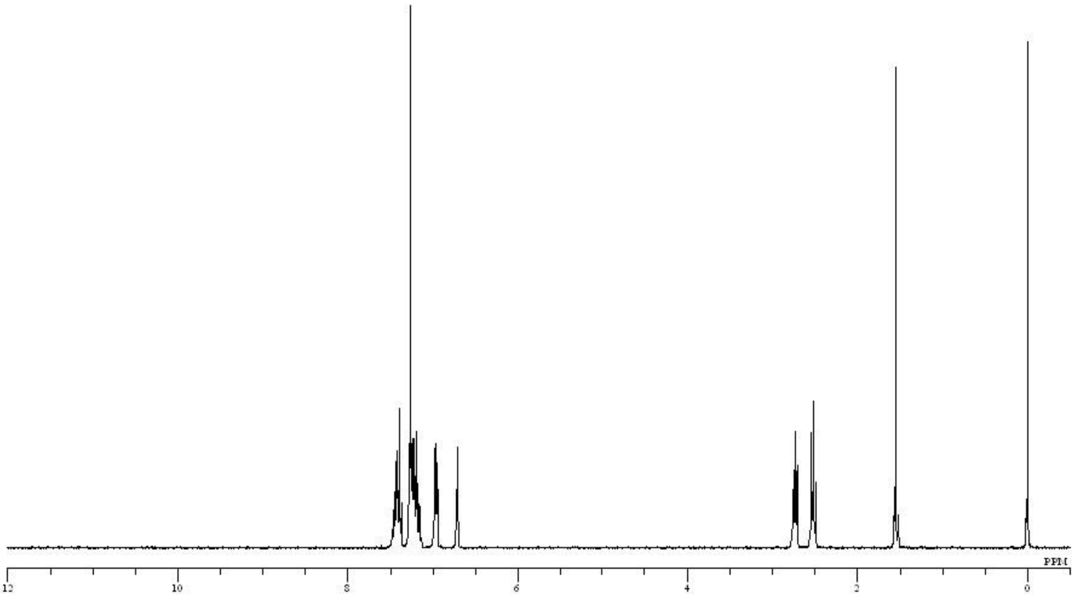

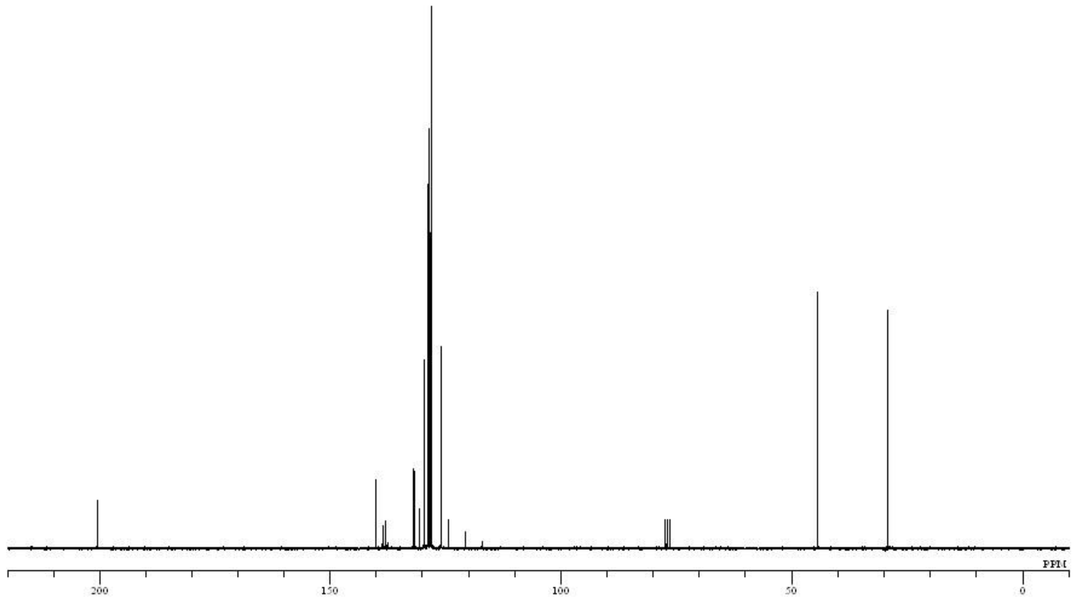

(*Z*)-6,6,6-Trifluoro-1,5-diphenylhex-4-en-3-one ((*Z*)-10h)

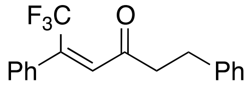

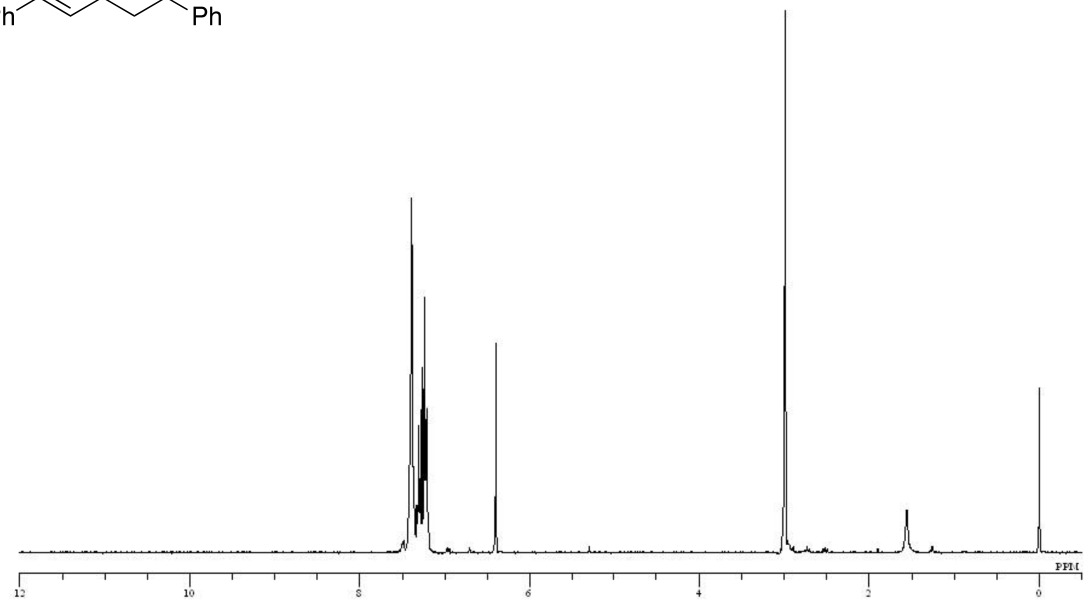

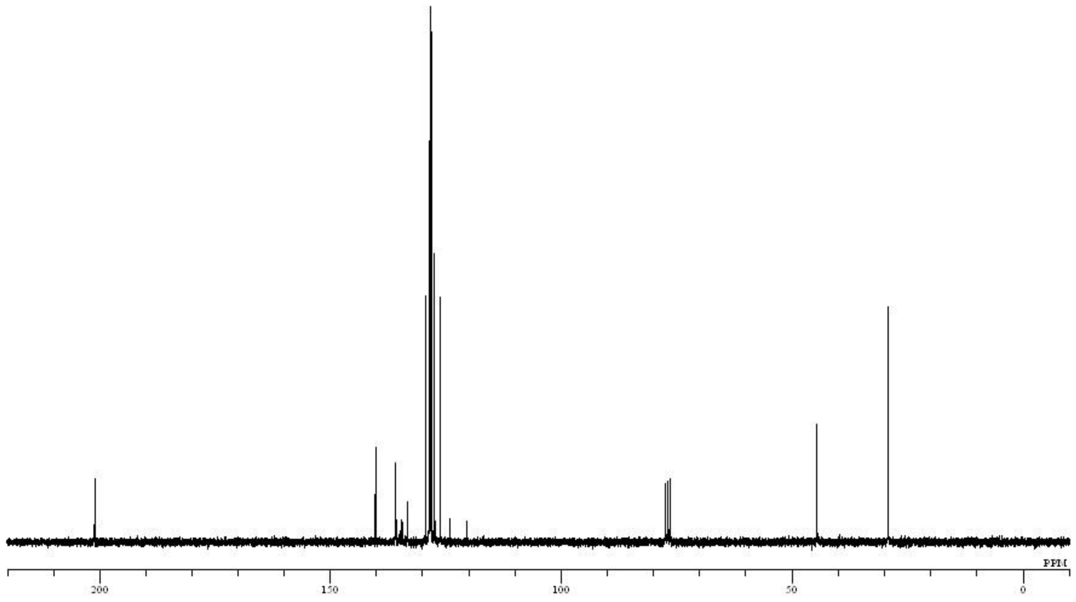

(*E*)-6,6,6-Trifluoro-5-phenylhex-4-en-3-one ((*E*)-10i)

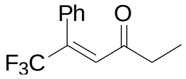

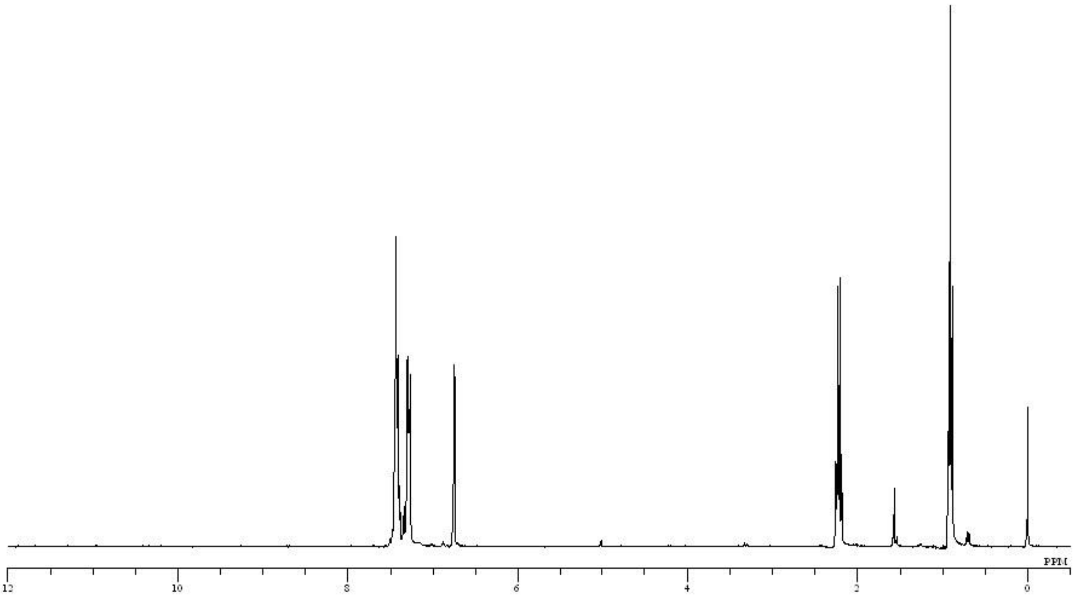

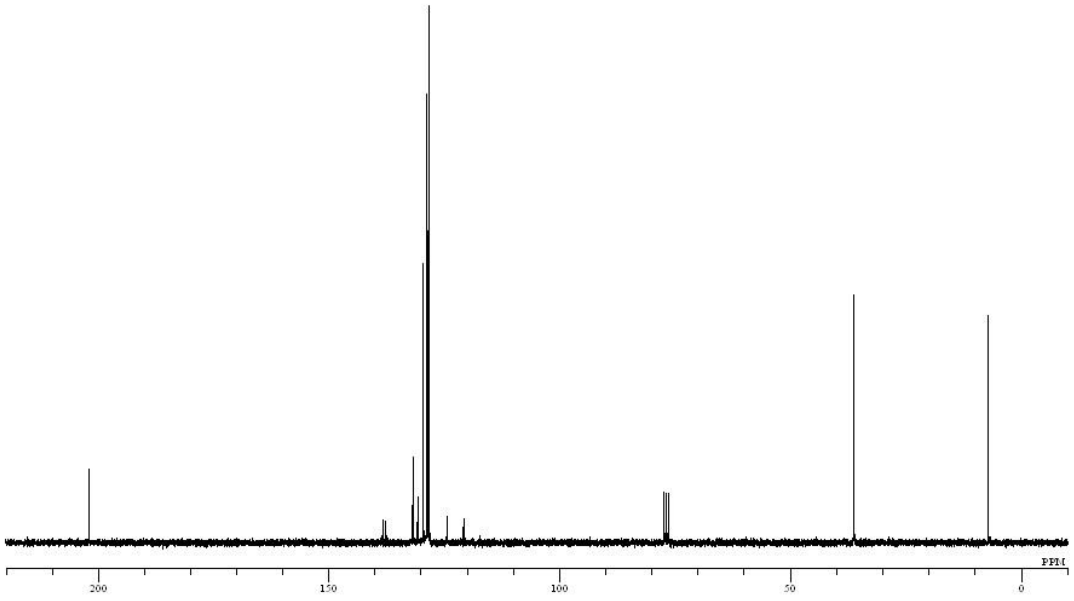

(*E*)-4,4,4-Tri- fluoro-1,3-diphenylbut-2-en-1-ol ((*E*)-6a)

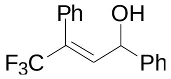

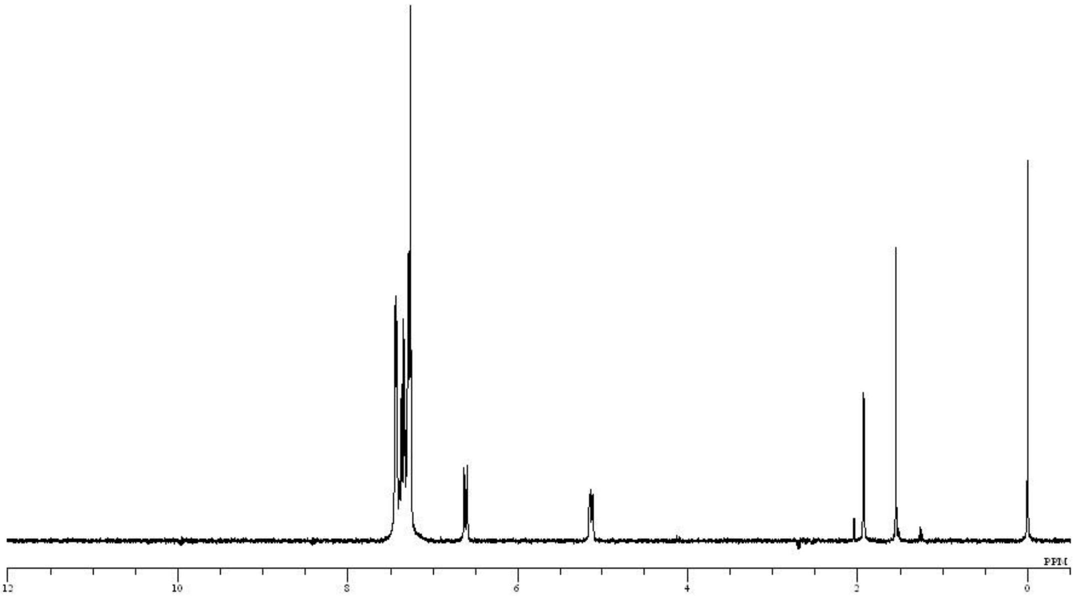

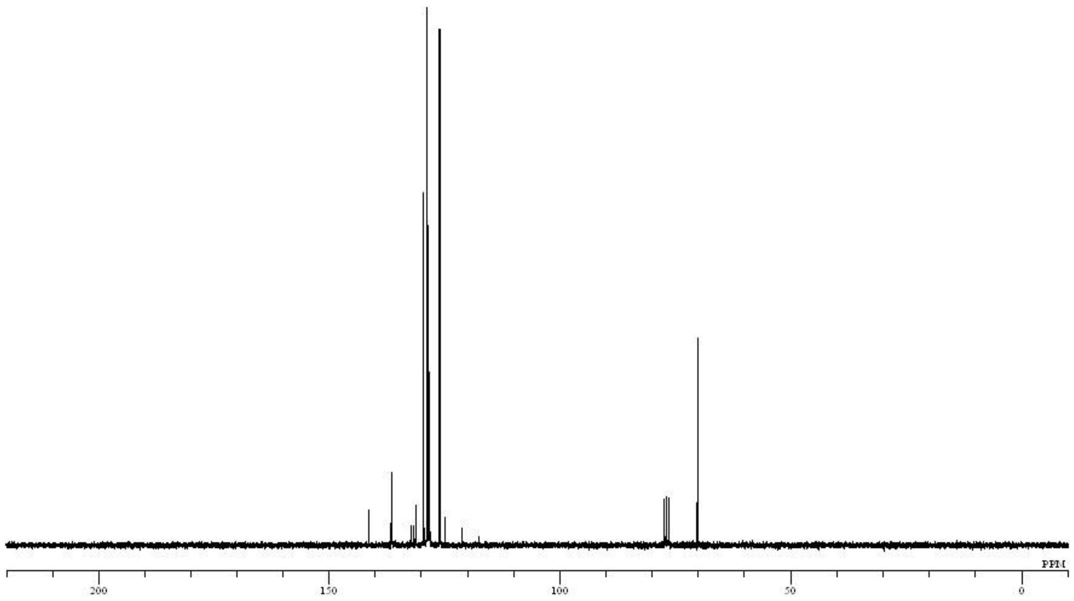

(*E*)-4,4,4-Trifluoro-3-(4-methoxyphenyl)-1-phenylbut-2-en-1-ol ((*E*)-6b)

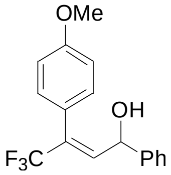

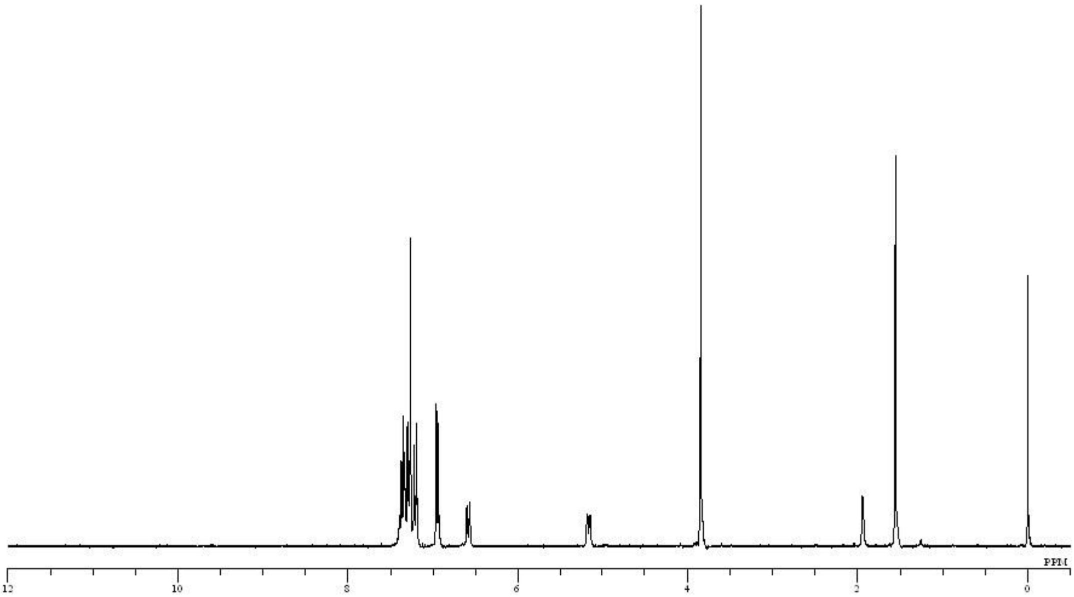

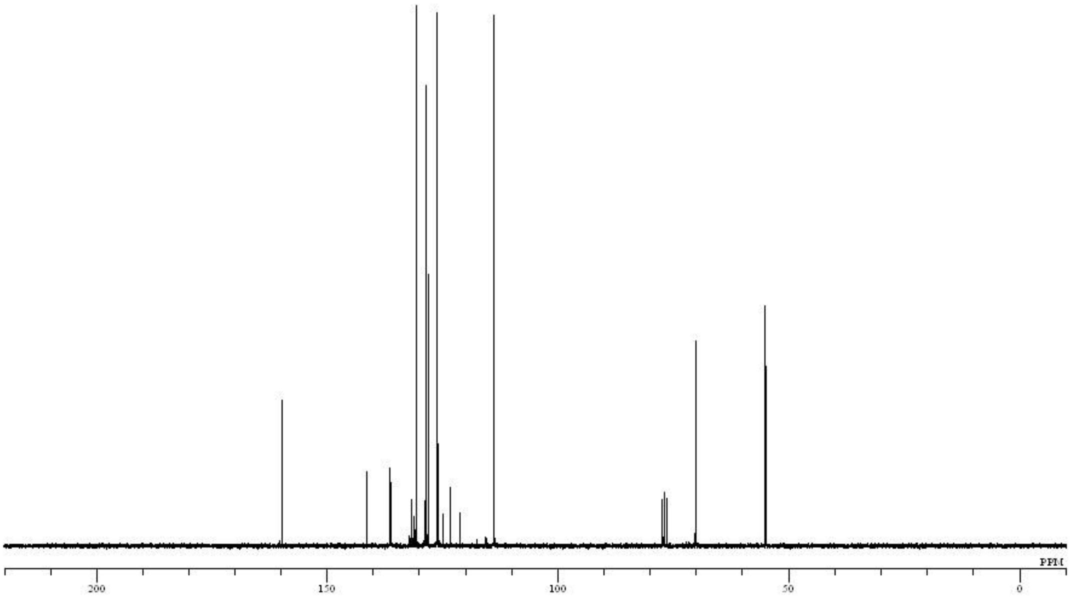

(*E*)-4,4,4-Trifluoro-3-(4-fluorophenyl)-1-phenylbut-2-en-1-ol ((*E*)-6c)

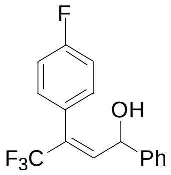

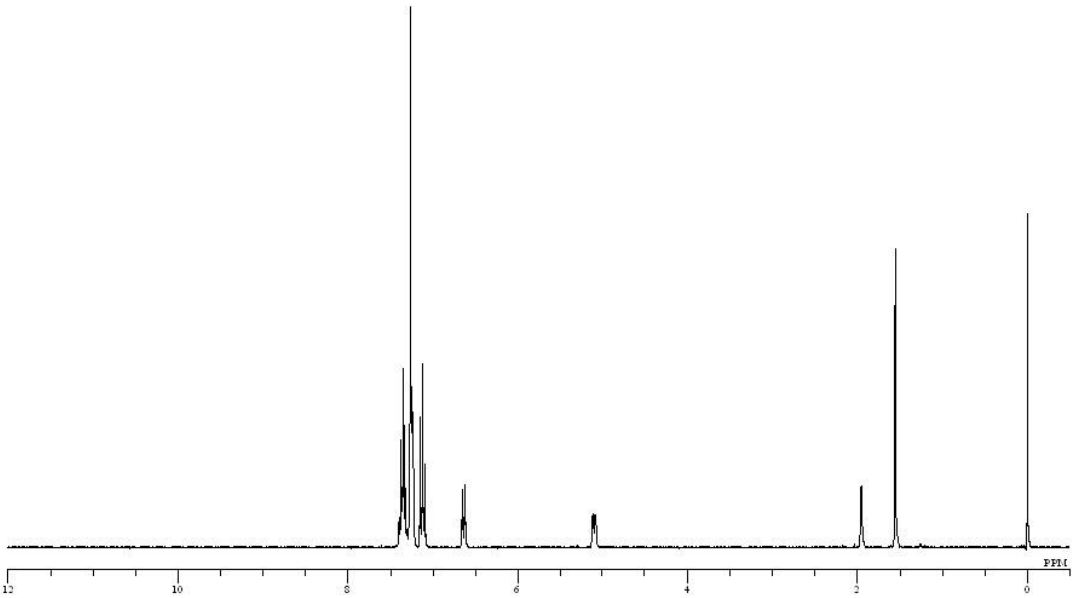

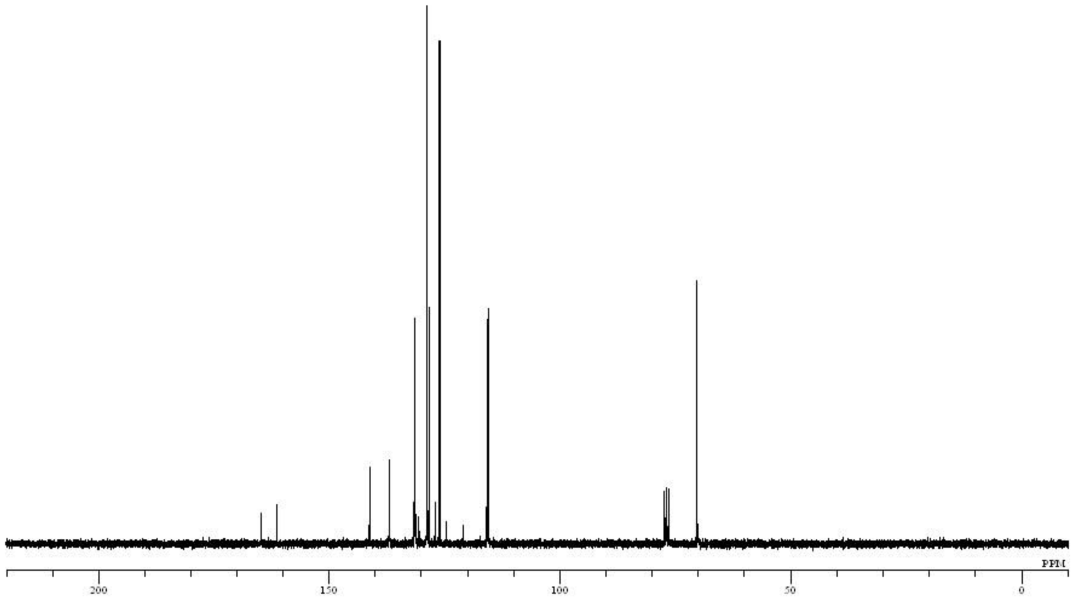

(*E*)-1-Phenyl-3-(trifluoromethyl)pent-2-en-1-ol ((*E*)-6d)

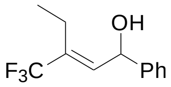

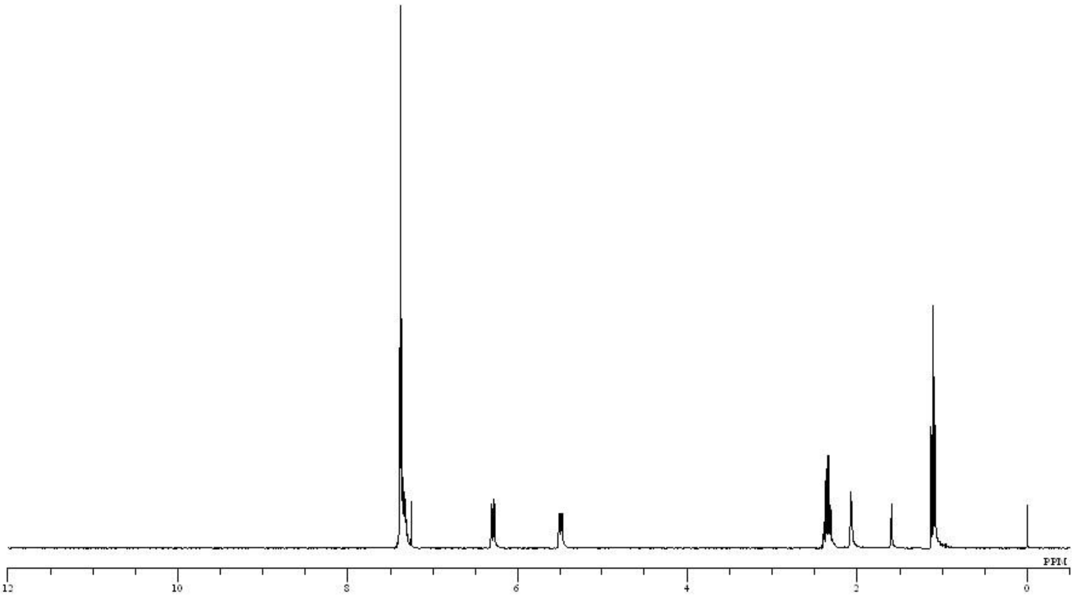

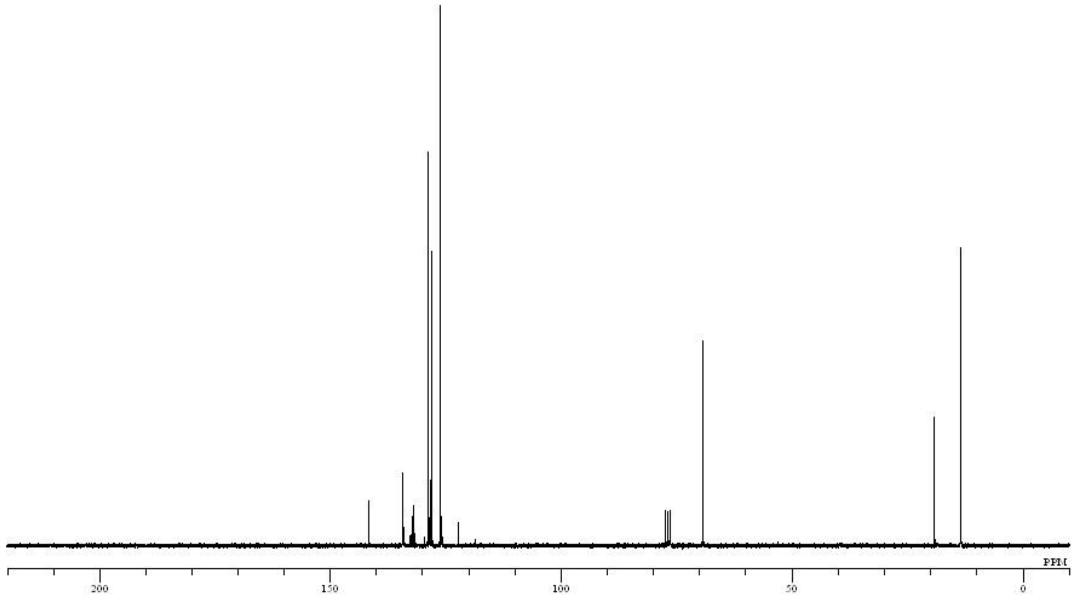

(*E*)-1,5-Diphenyl-3-(trifluoromethyl)pent-2-en-1-ol ((*E*)-6e)

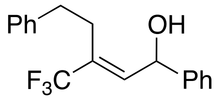

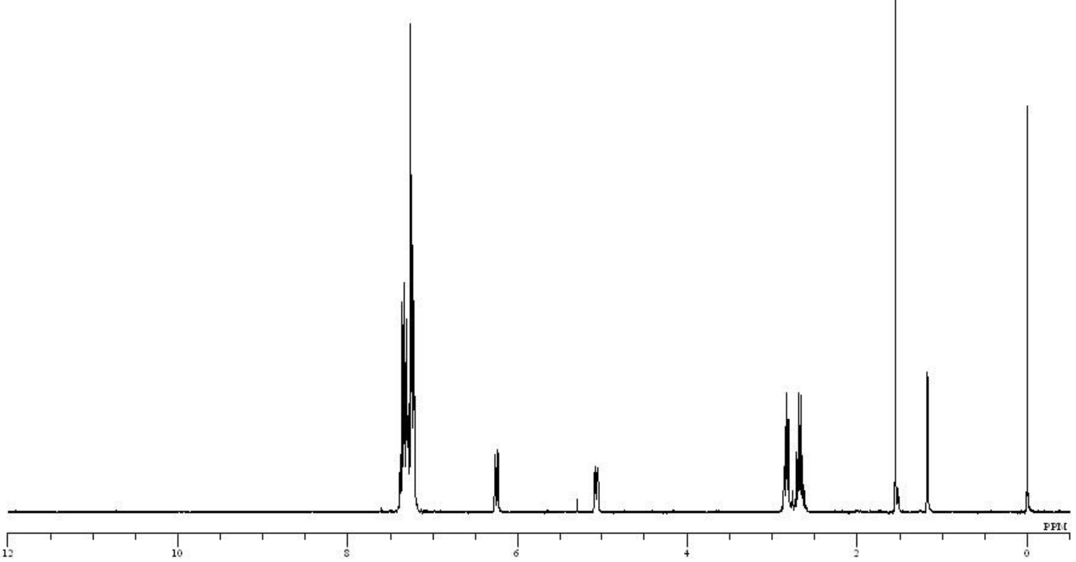

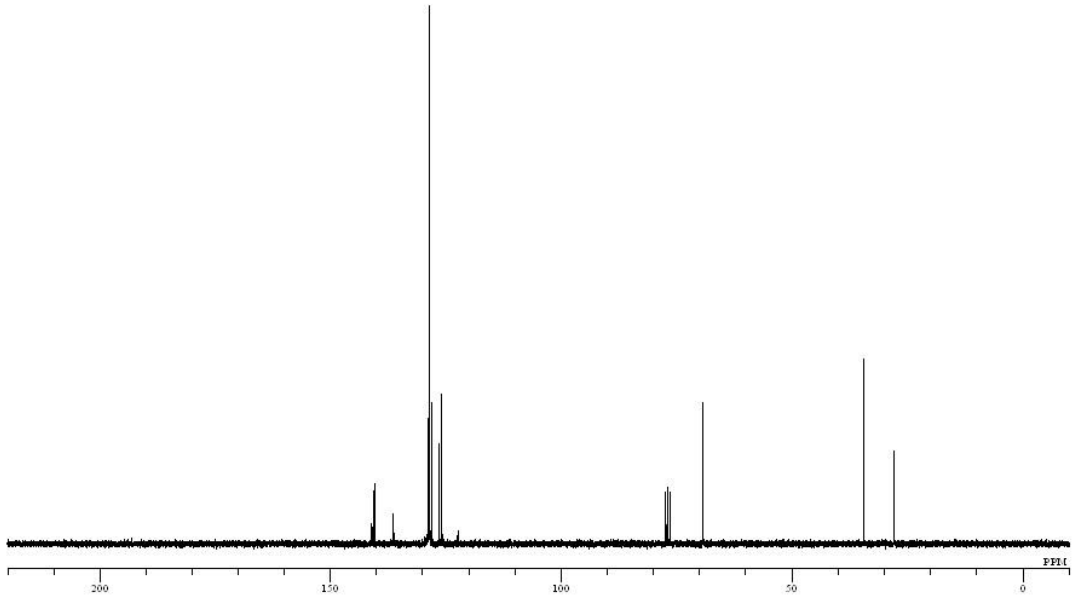

(*E*)-1-(4-Methoxyphenyl)-5-phenyl-3-(trifluoromethyl)pent-2-en-1-ol ((*E*)-6f)

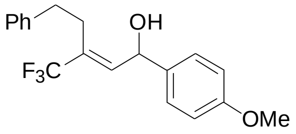

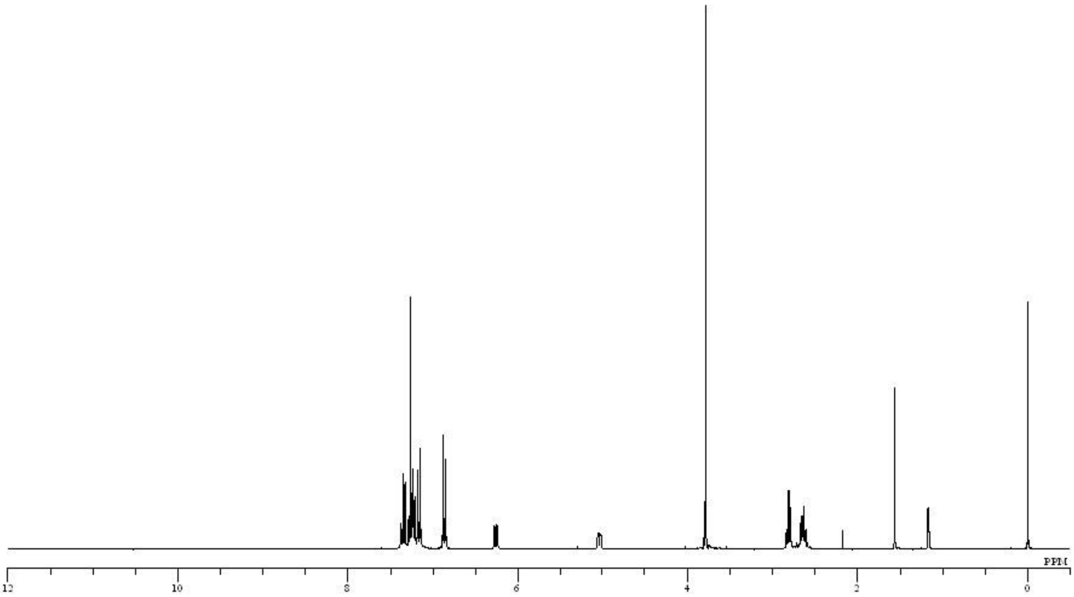

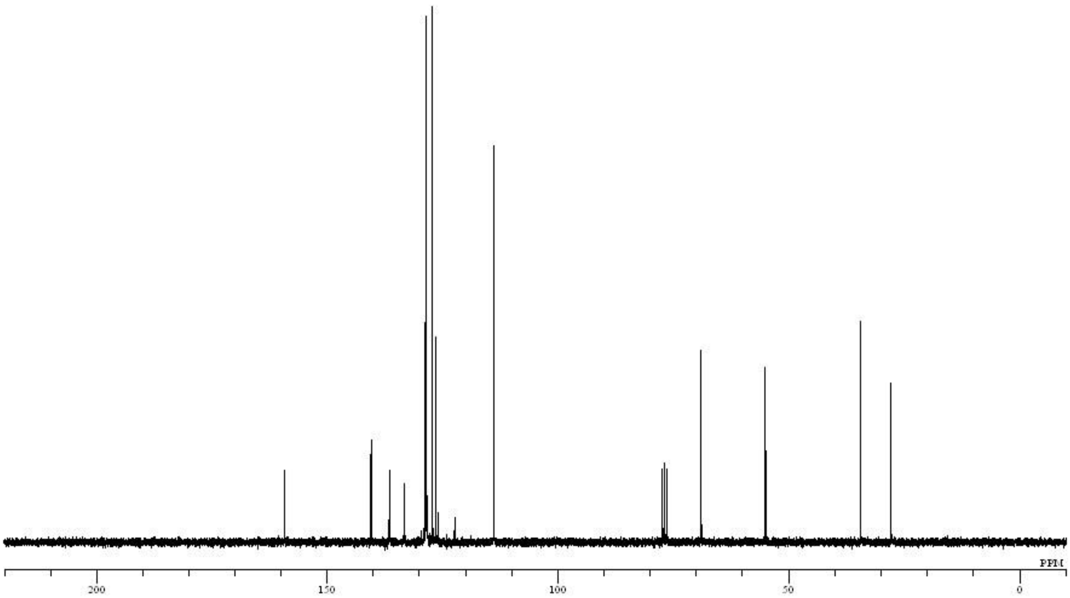

(*E*)-1-(4-Bromophenyl)-5-phenyl-3-(trifluoromethyl)pent-2-en-1-ol ((*E*)-6g)

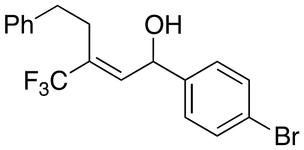

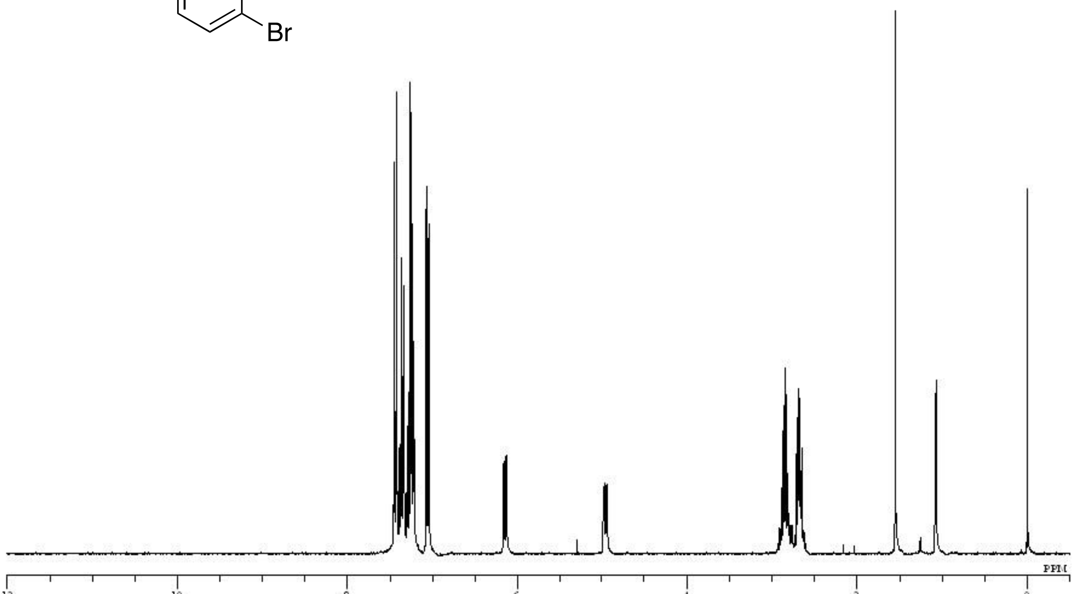

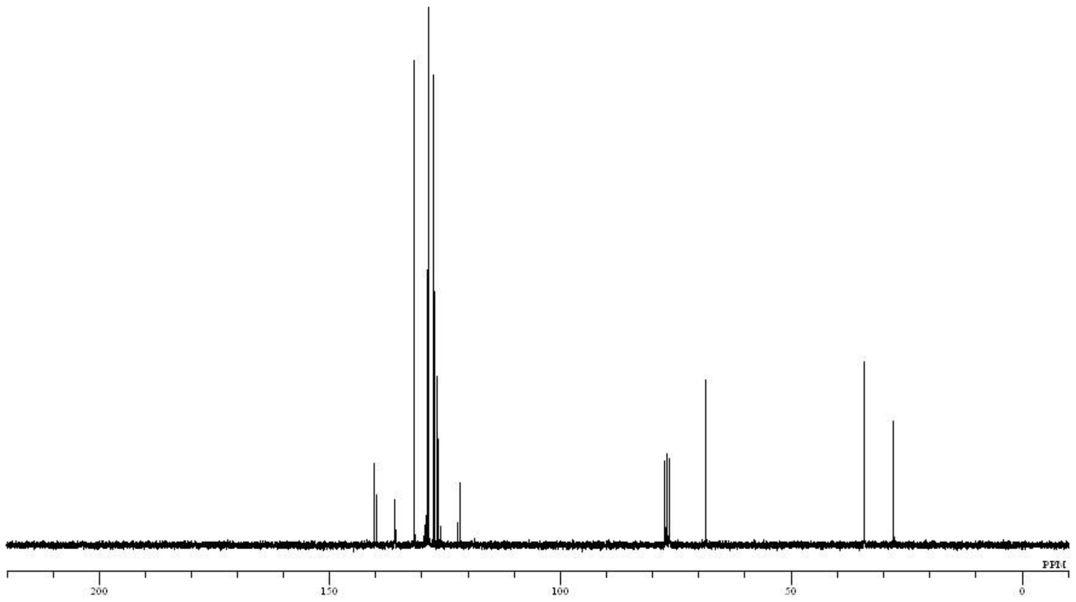

(*E*)-6,6,6-Trifluoro-1,5-diphenylhex-4-en-3-ol ((*E*)-6h)

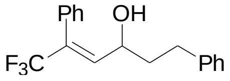

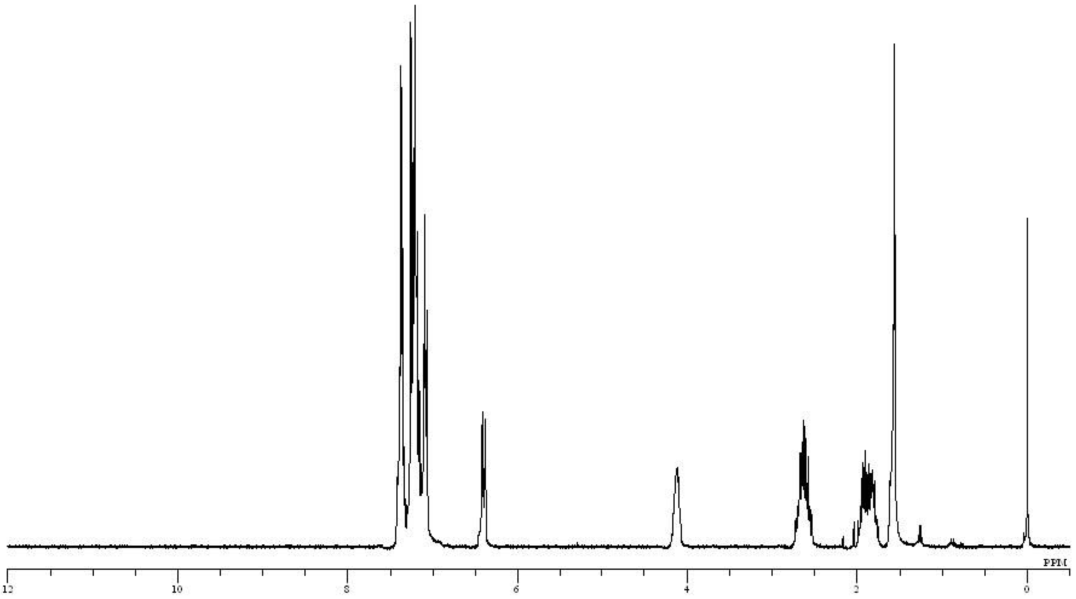

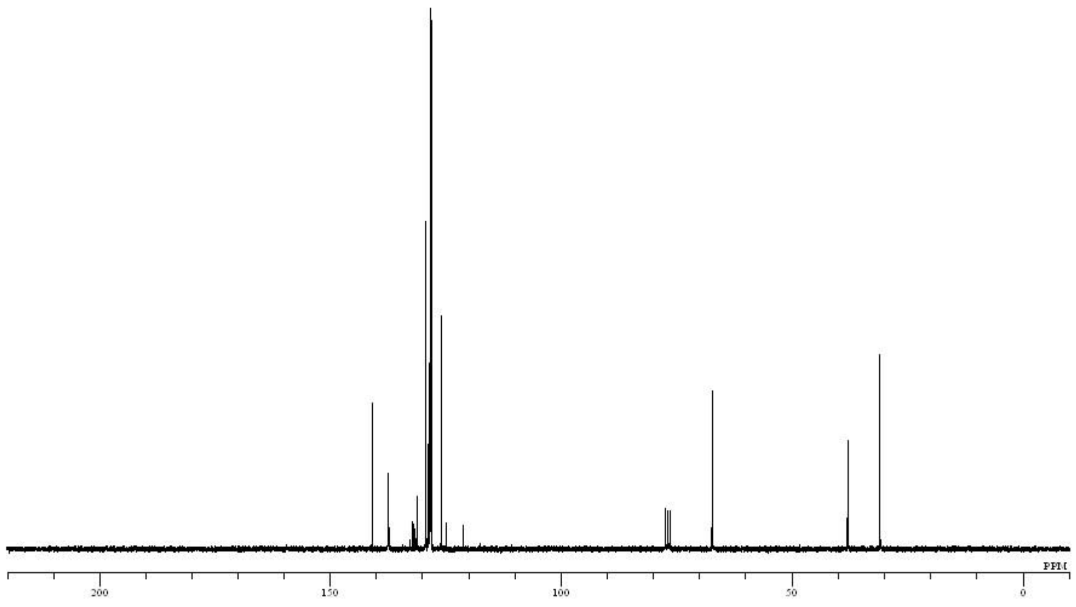

(*E*)-6,6,6-Trifluoro-5-phenylhex-4-en-3-ol ((*E*)-6i)

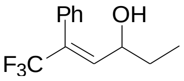

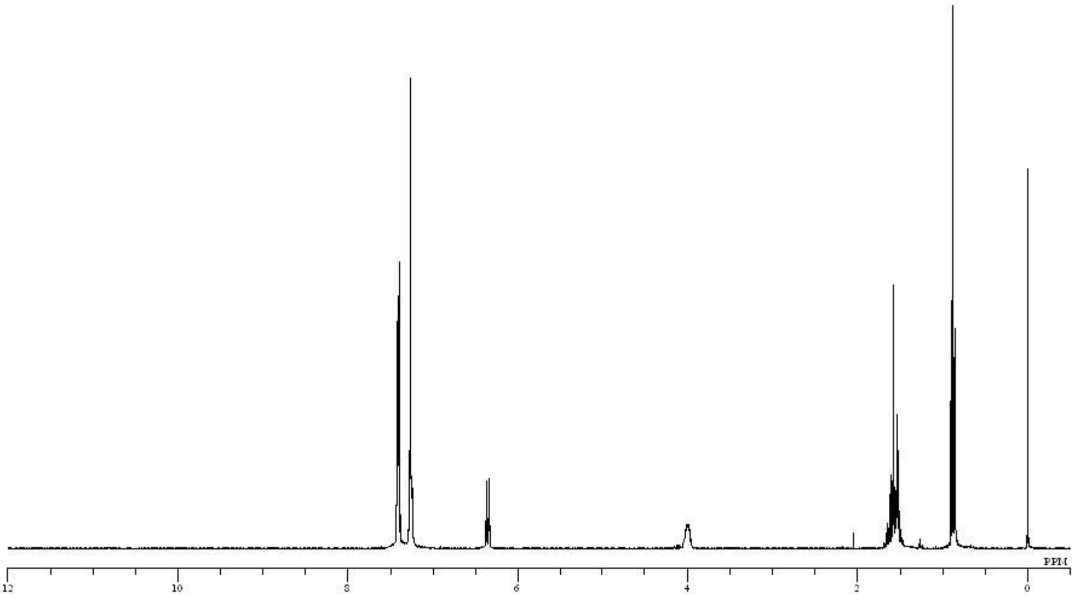

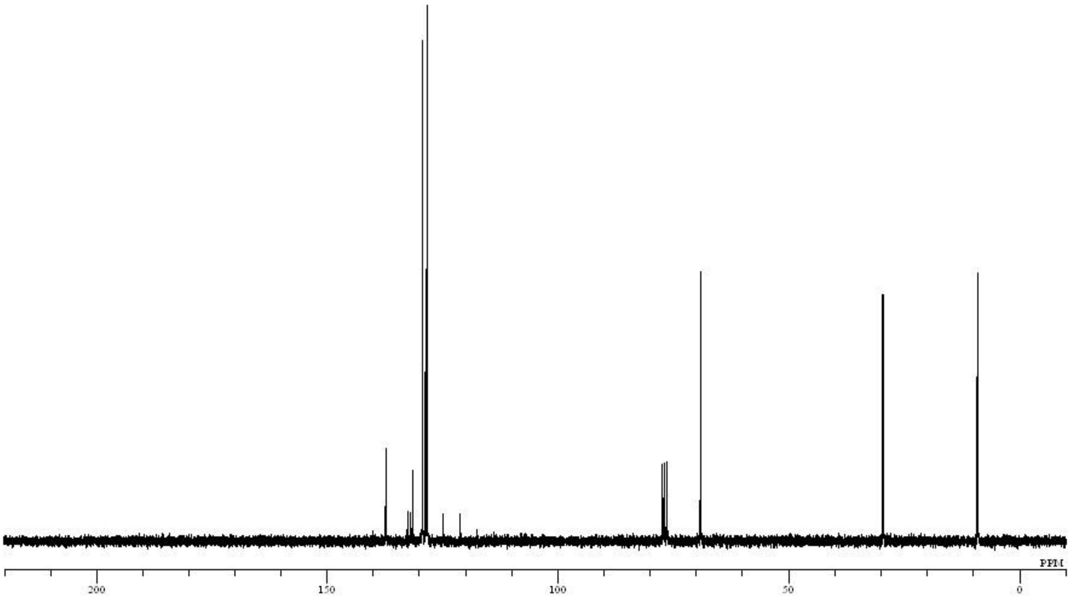

4,4,4-Trifluoro-1,3-diphenylbutan-1-one (7a)

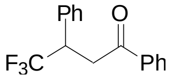

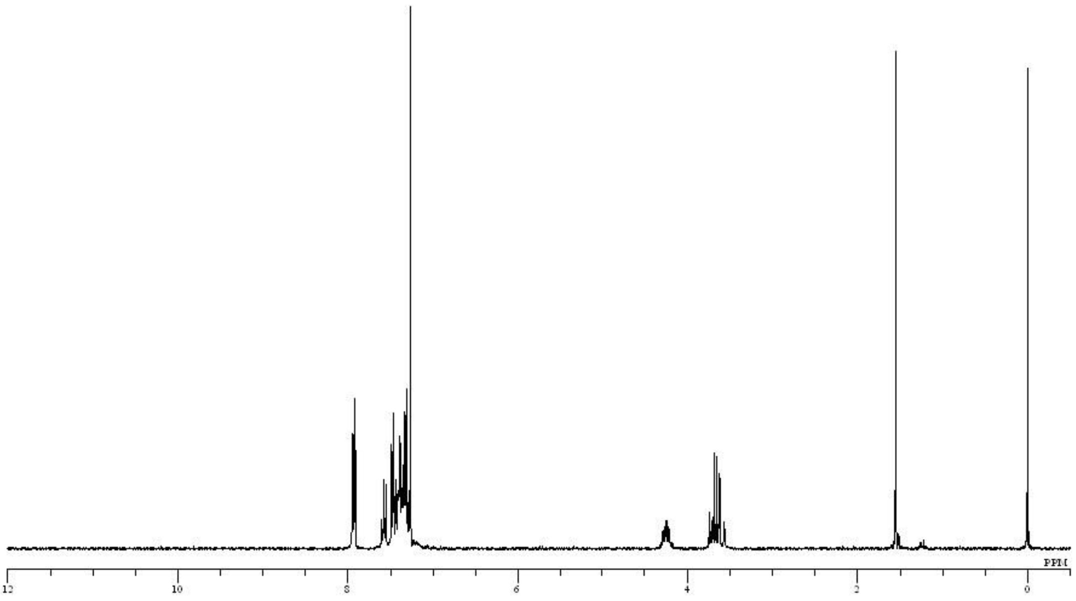

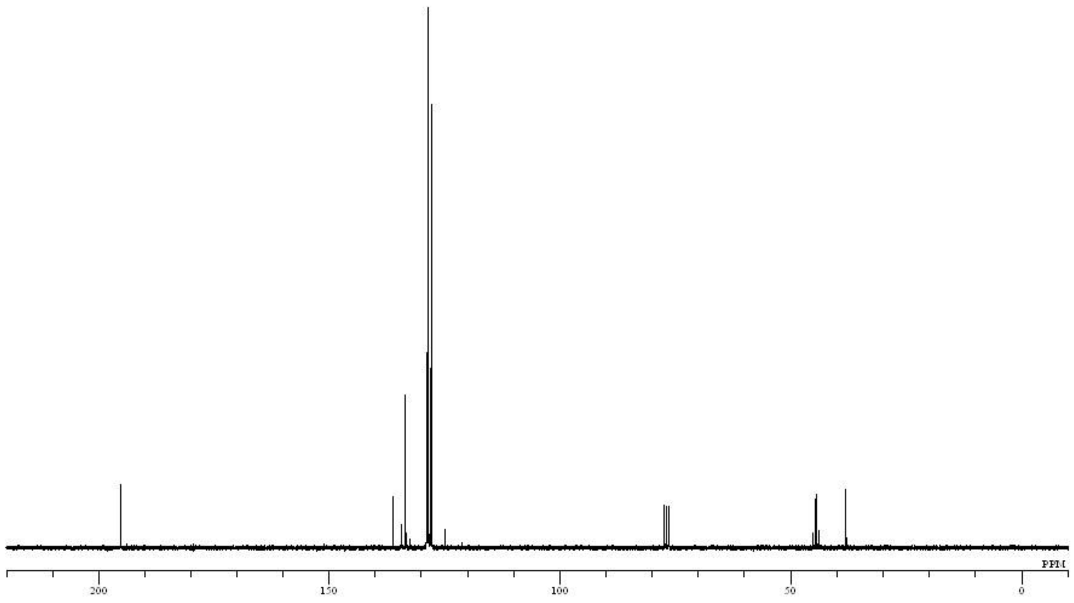

4,4,4-Trifluoro-3-(4-methoxyphenyl)-1-phenylbutan-1-one (7b)

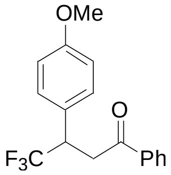

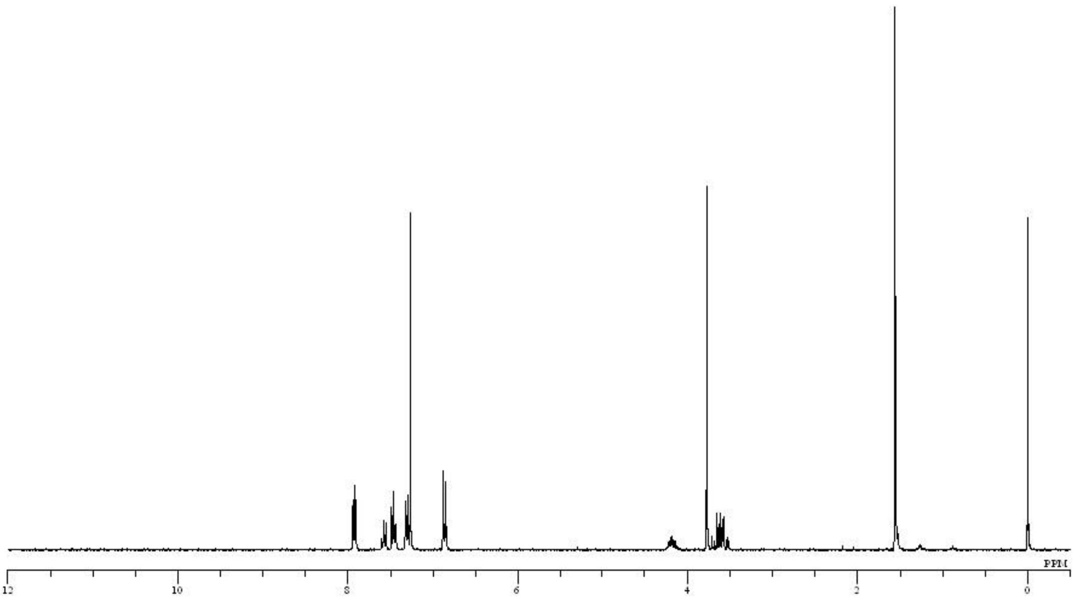

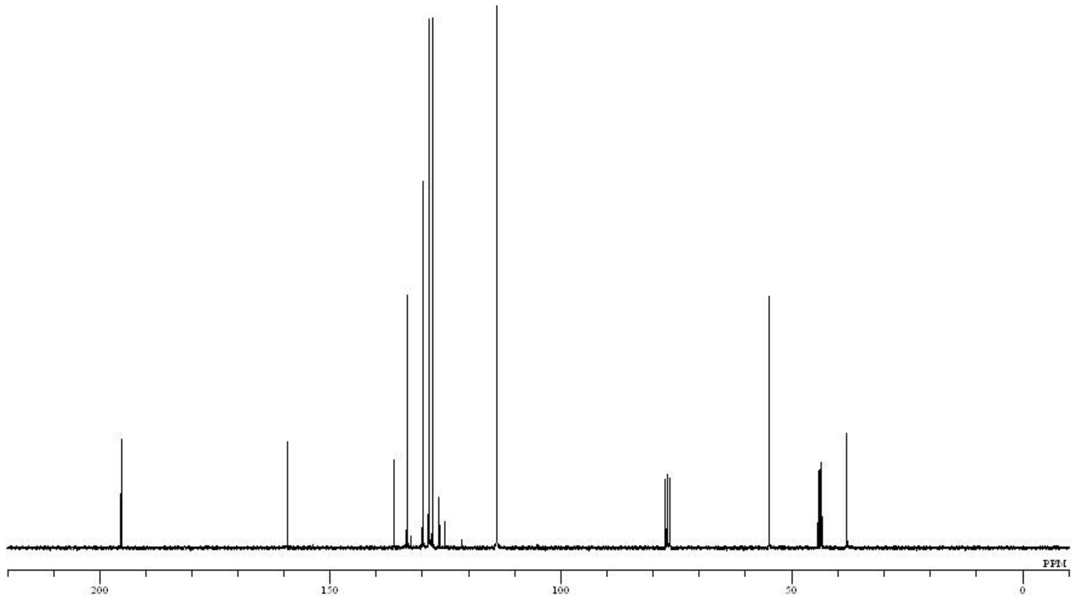

4,4,4-Trifluoro-3-(4-fluorophenyl)-1-phenylbutan-1-one (7c)

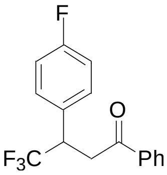

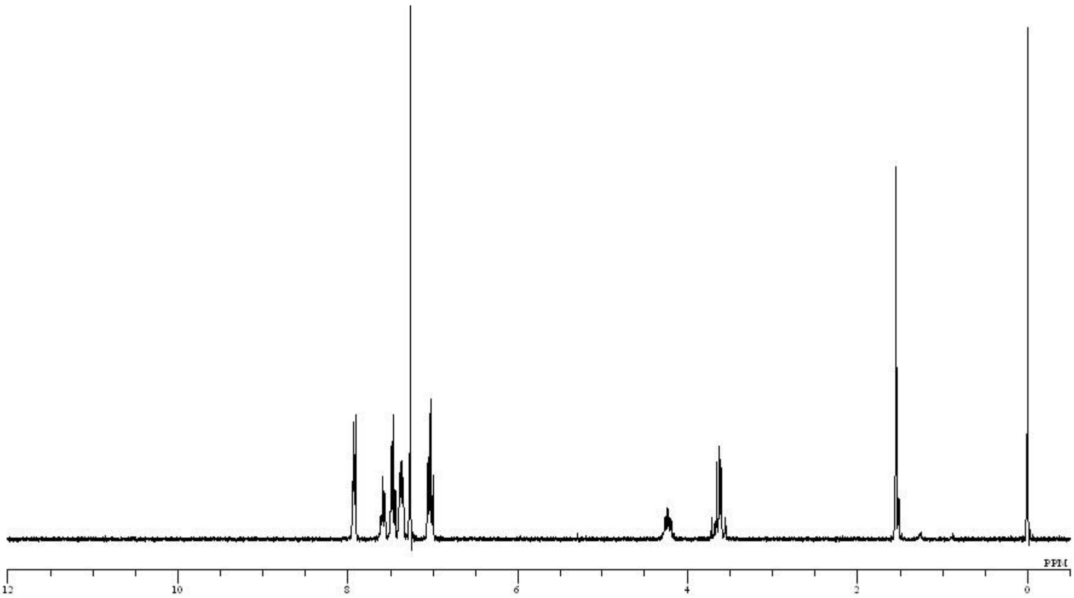

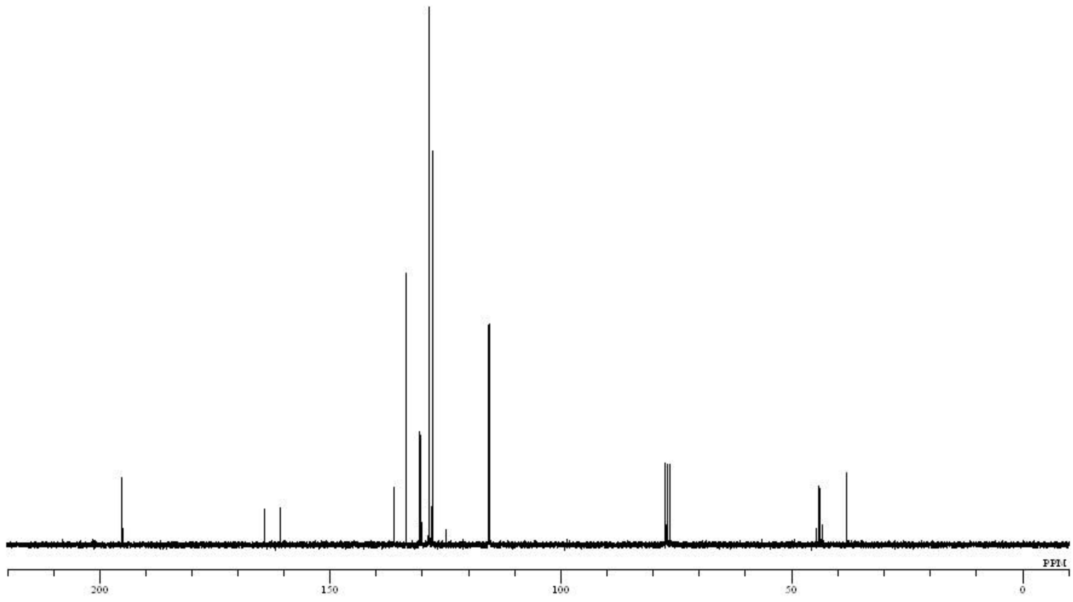

1-Phenyl-3-(trifluoromethyl)pentan-1-one (7d)

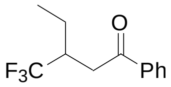

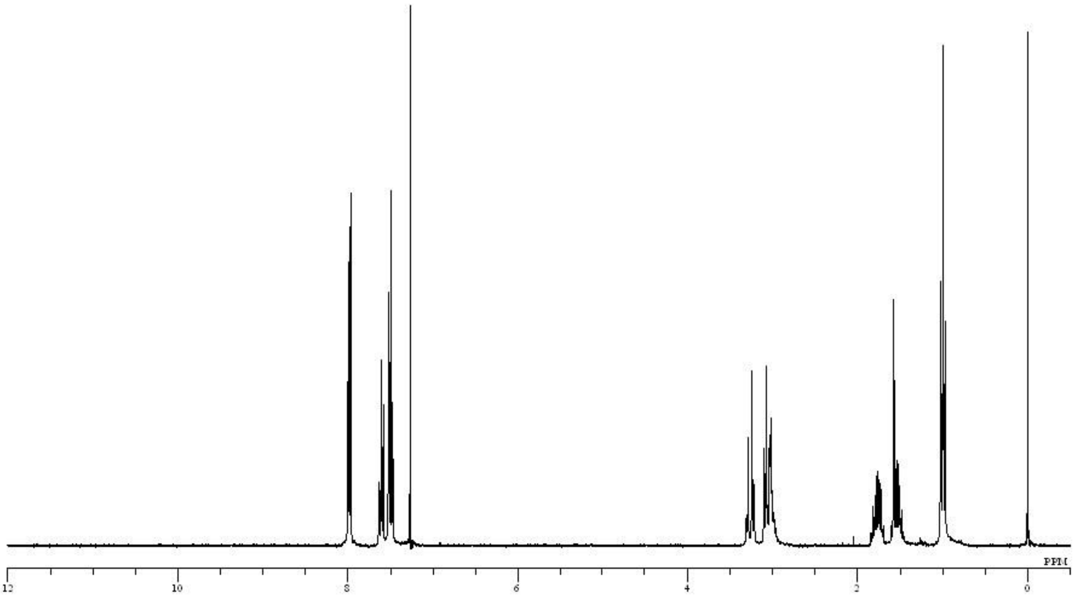

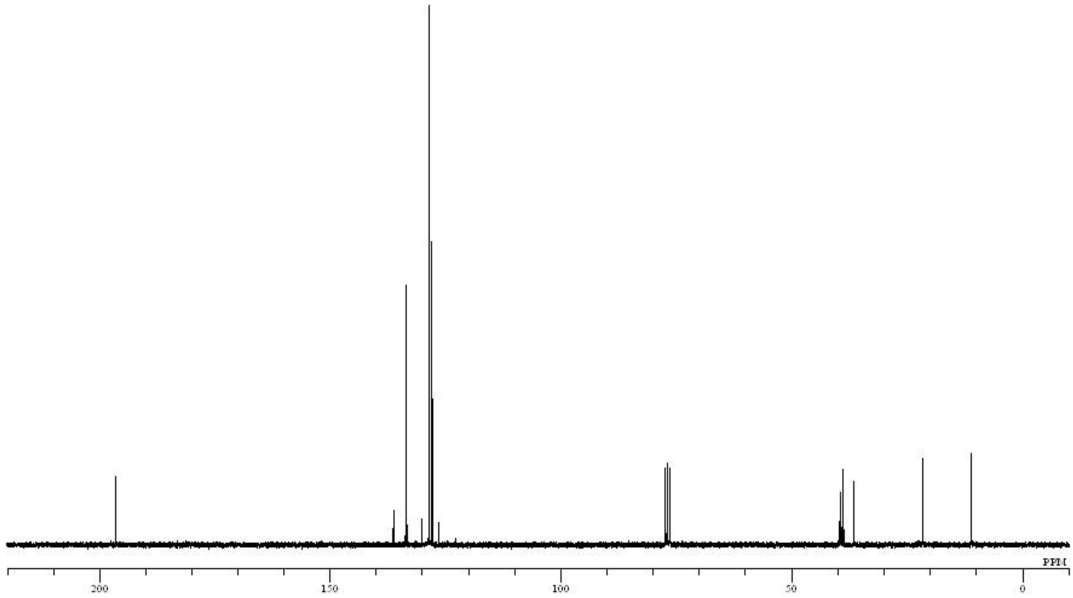

1,5-Diphenyl-3-(trifluoromethyl)pentan-1-one (7e)

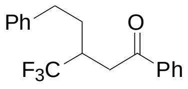

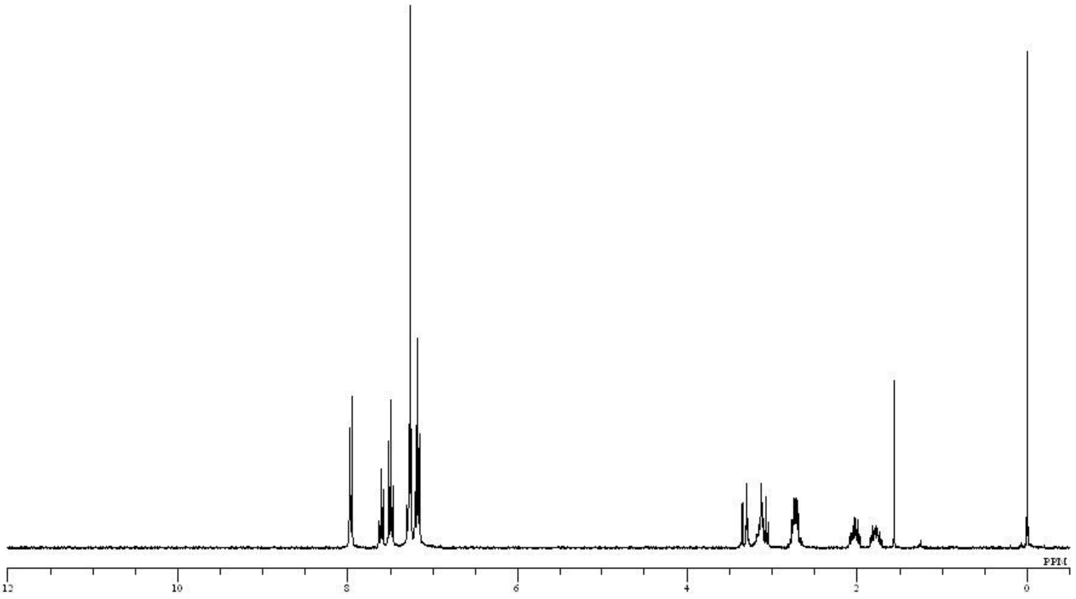

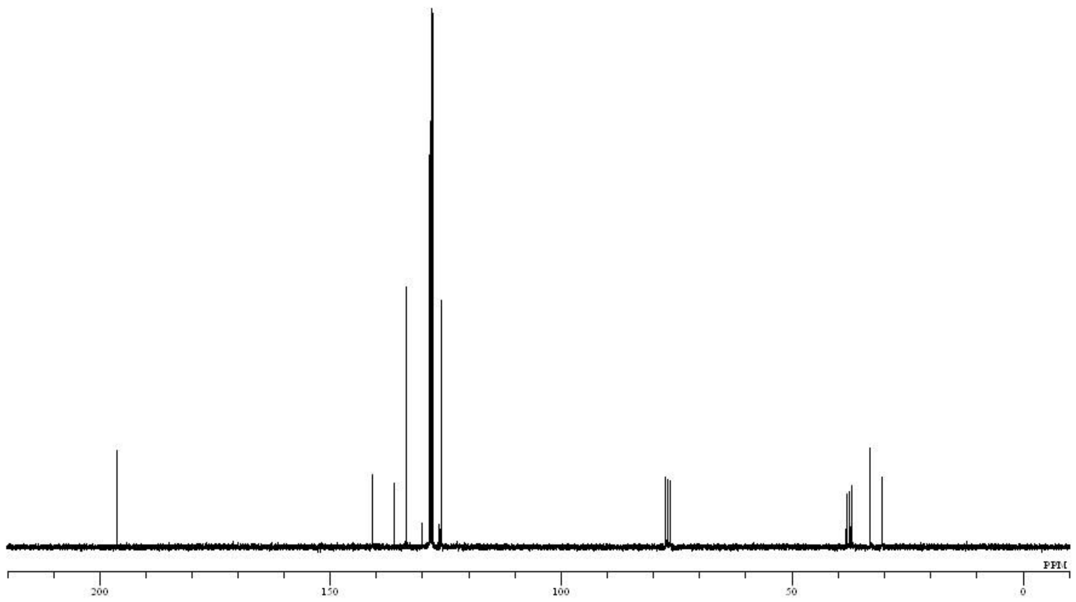

1-(4-Methoxyphenyl)-5-phenyl-3-(trifluoromethyl)pentan-1-one (7f)

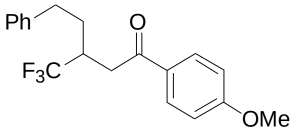

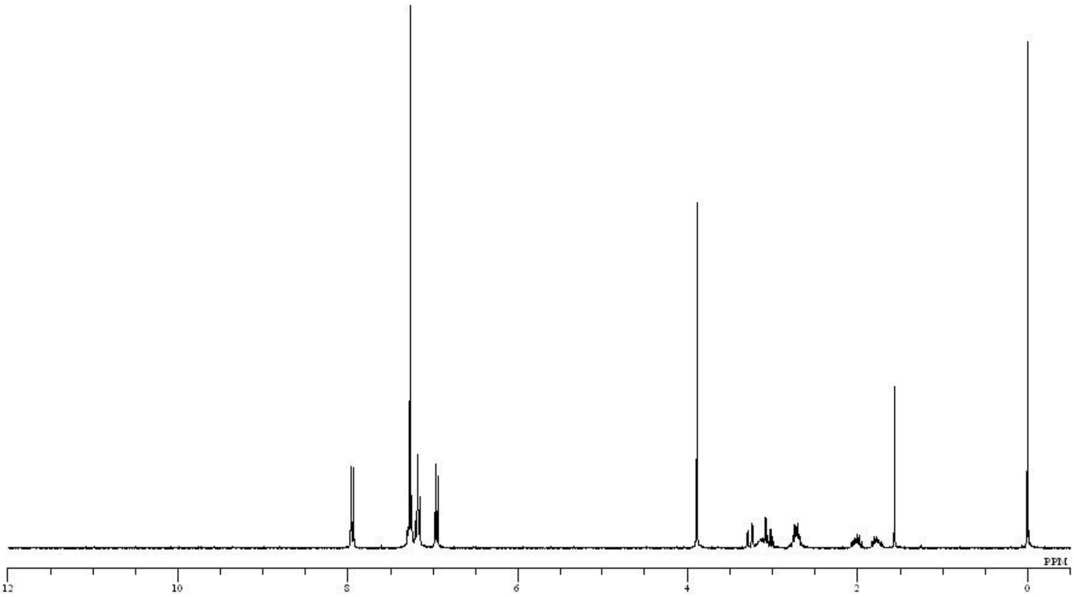

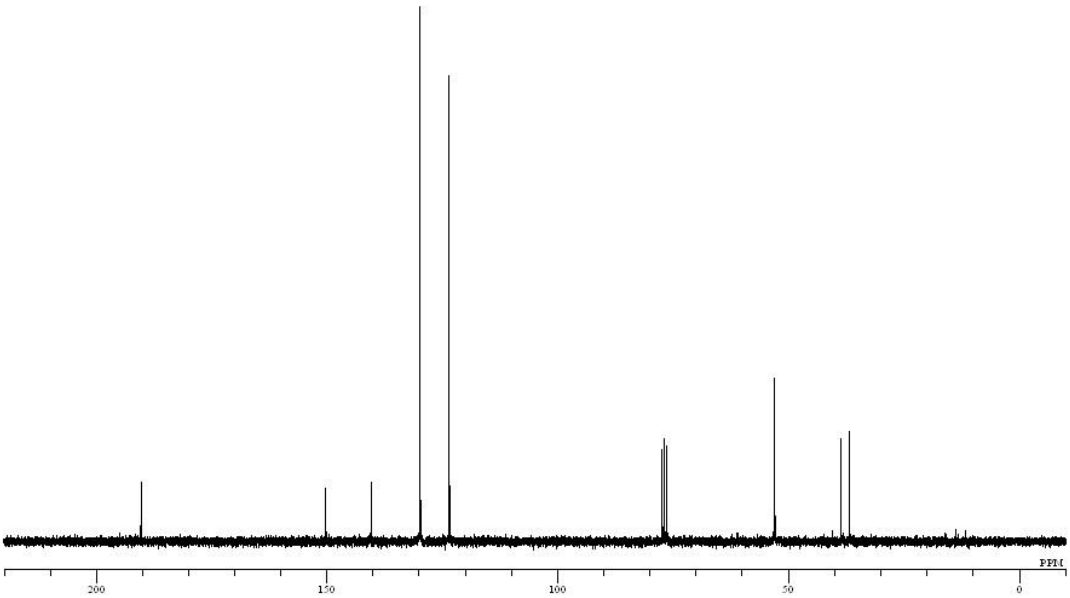

1-(4-Bromophenyl)-5-phenyl-3-(trifluoromethyl)pentan-1-one (7g)

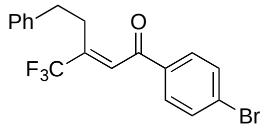

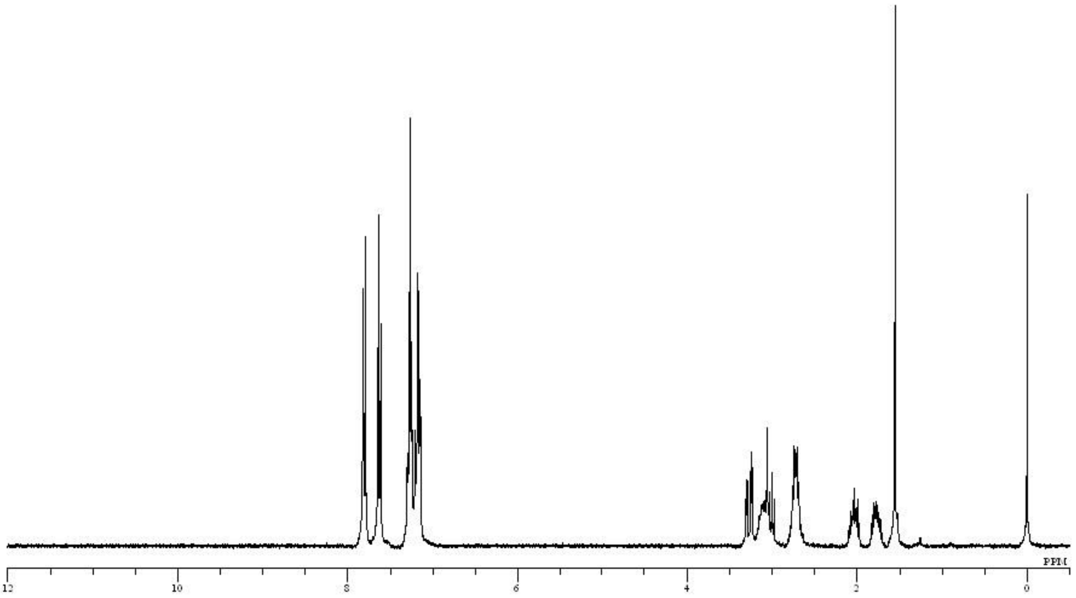

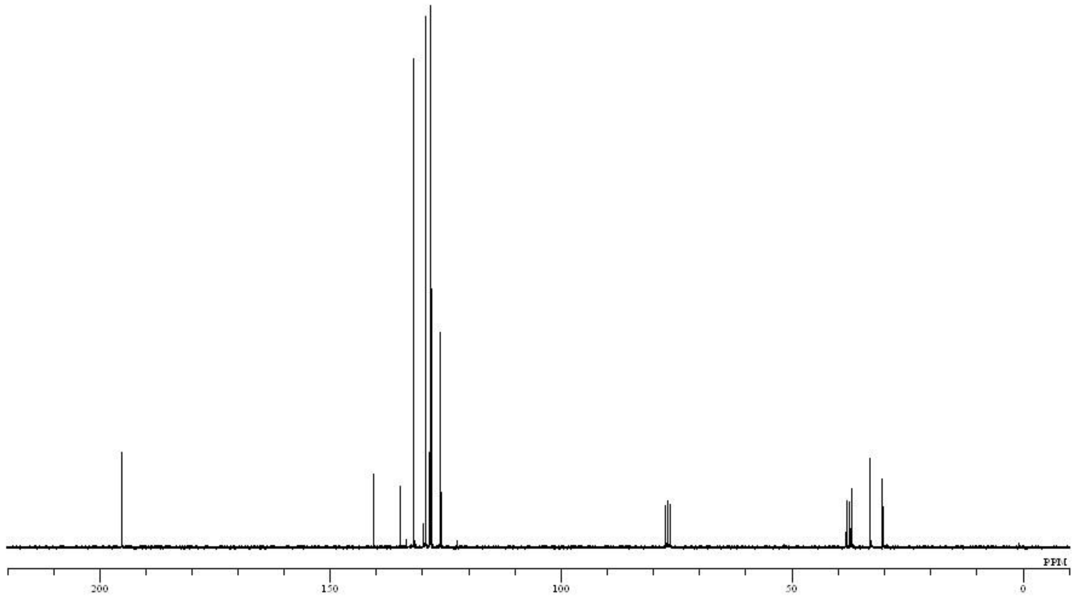

4. HPLC Charts

(*E*)-4,4,4-Trifluoro-1,3-diphenylbut-2-en-1-ol ((*E*)-6a)

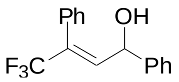

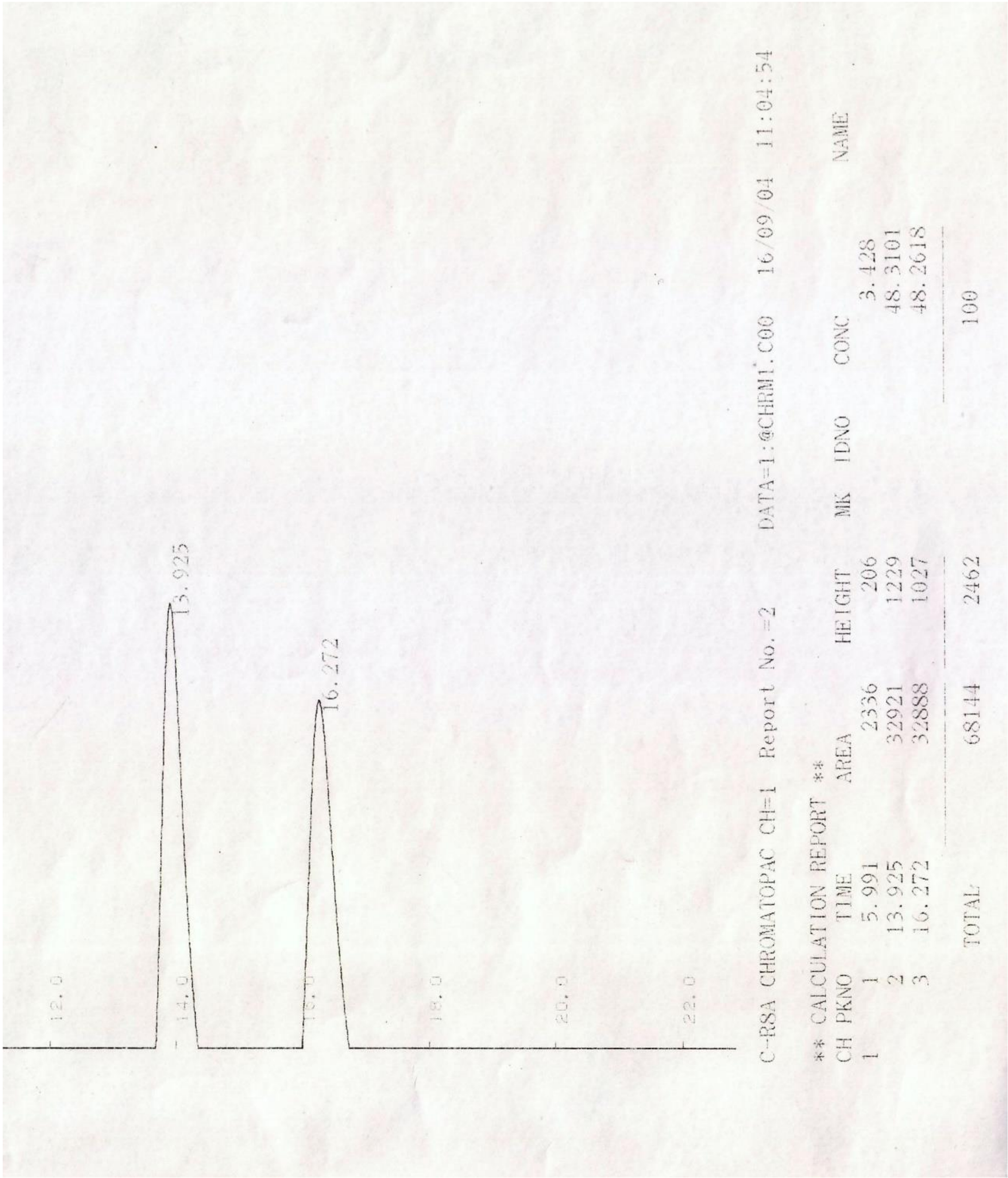

(*R*,*E*)-4,4,4-Trifluoro-1,3-diphenylbut-2-en-1-ol ((*R*,*E*)-6a)

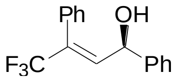

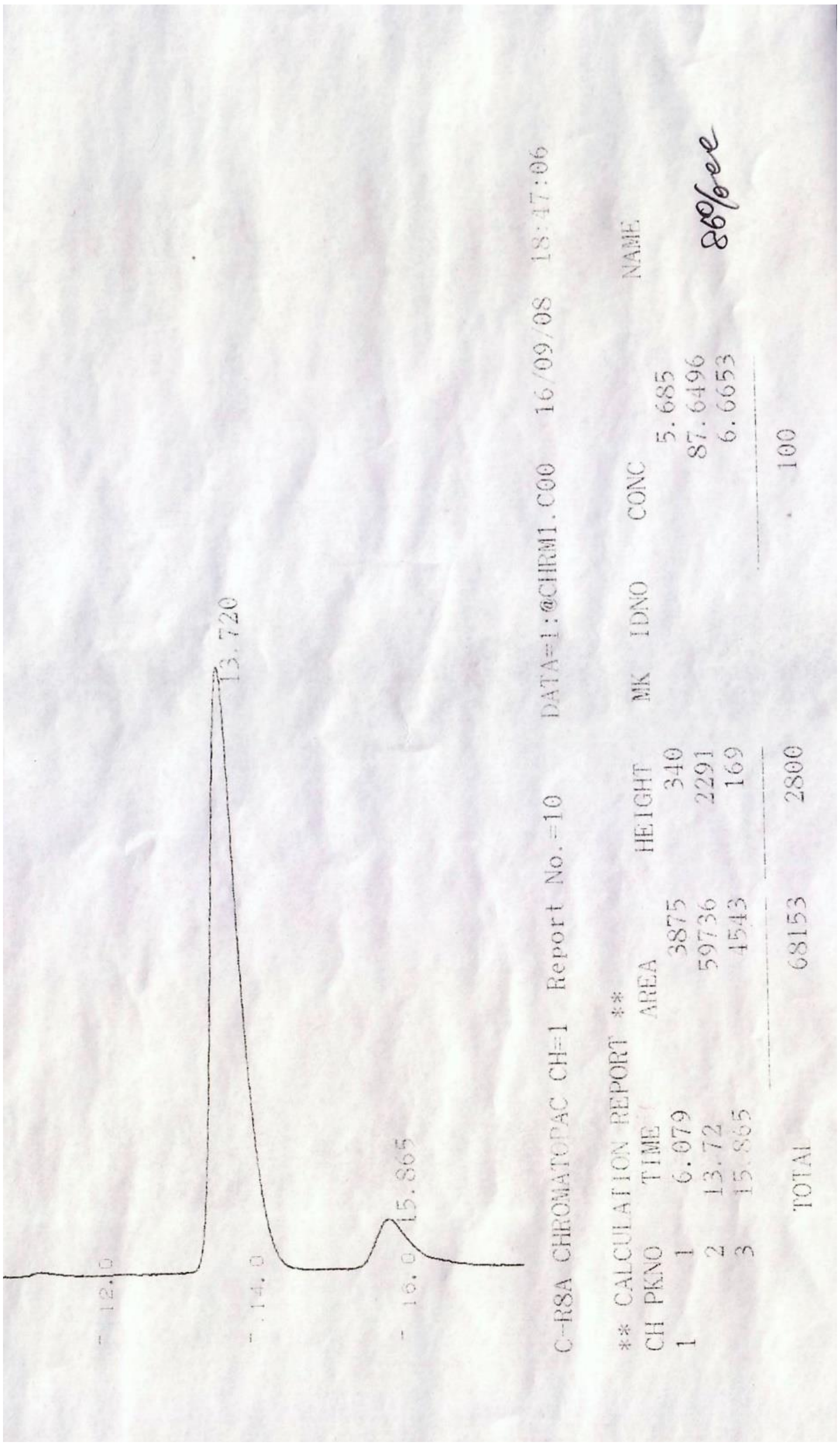

(*E*)-4,4,4-Trifluoro-3-(4-methoxyphenyl)-1-phenylbut-2-en-1-ol ((*E*)-6b)

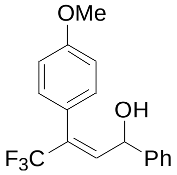

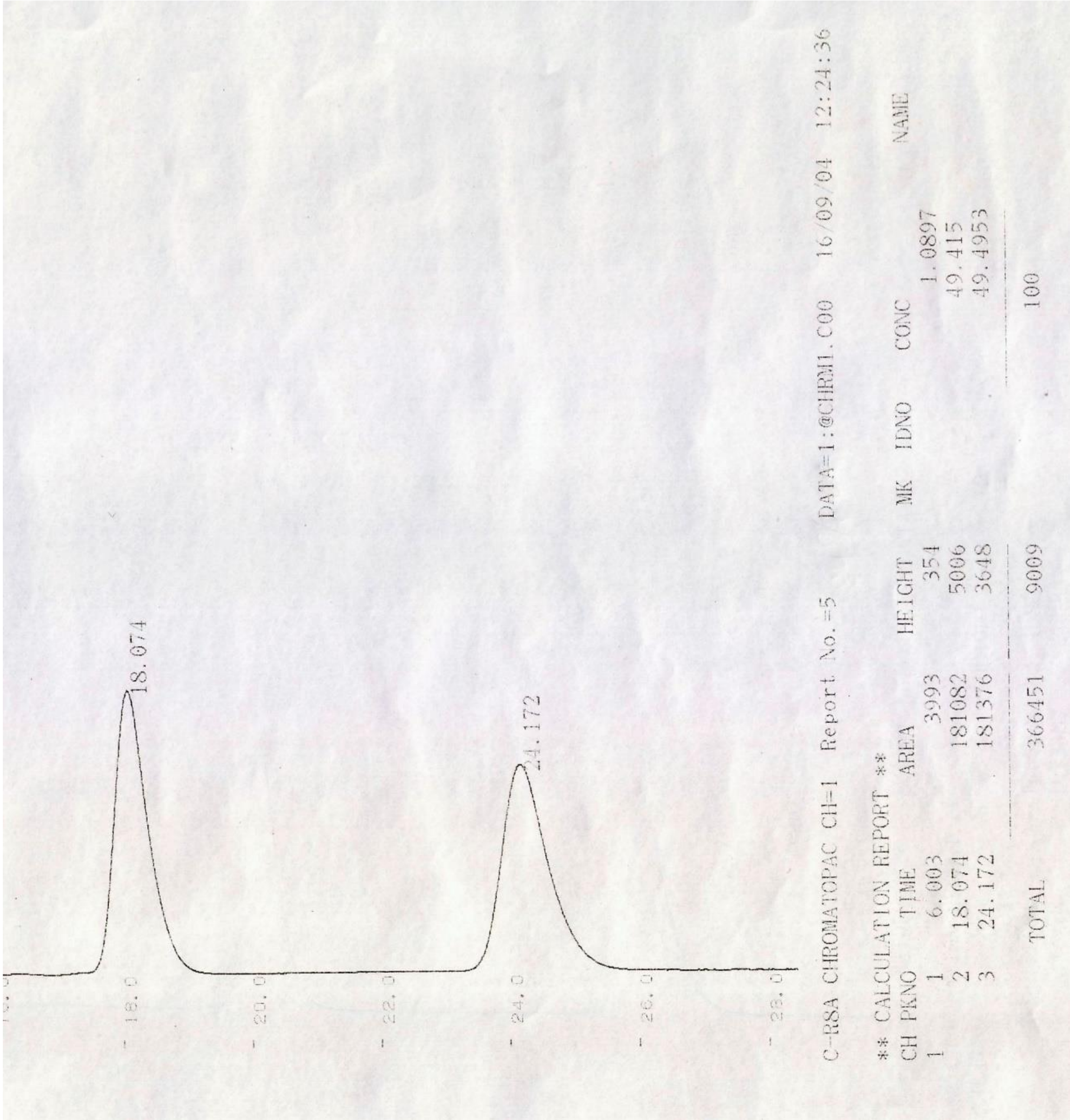

(*R*,*E*)-4,4,4-Trifluoro-3-(4-methoxyphenyl)-1-phenylbut-2-en-1-ol ((*R*,*E*)-6b)

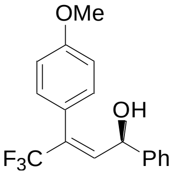

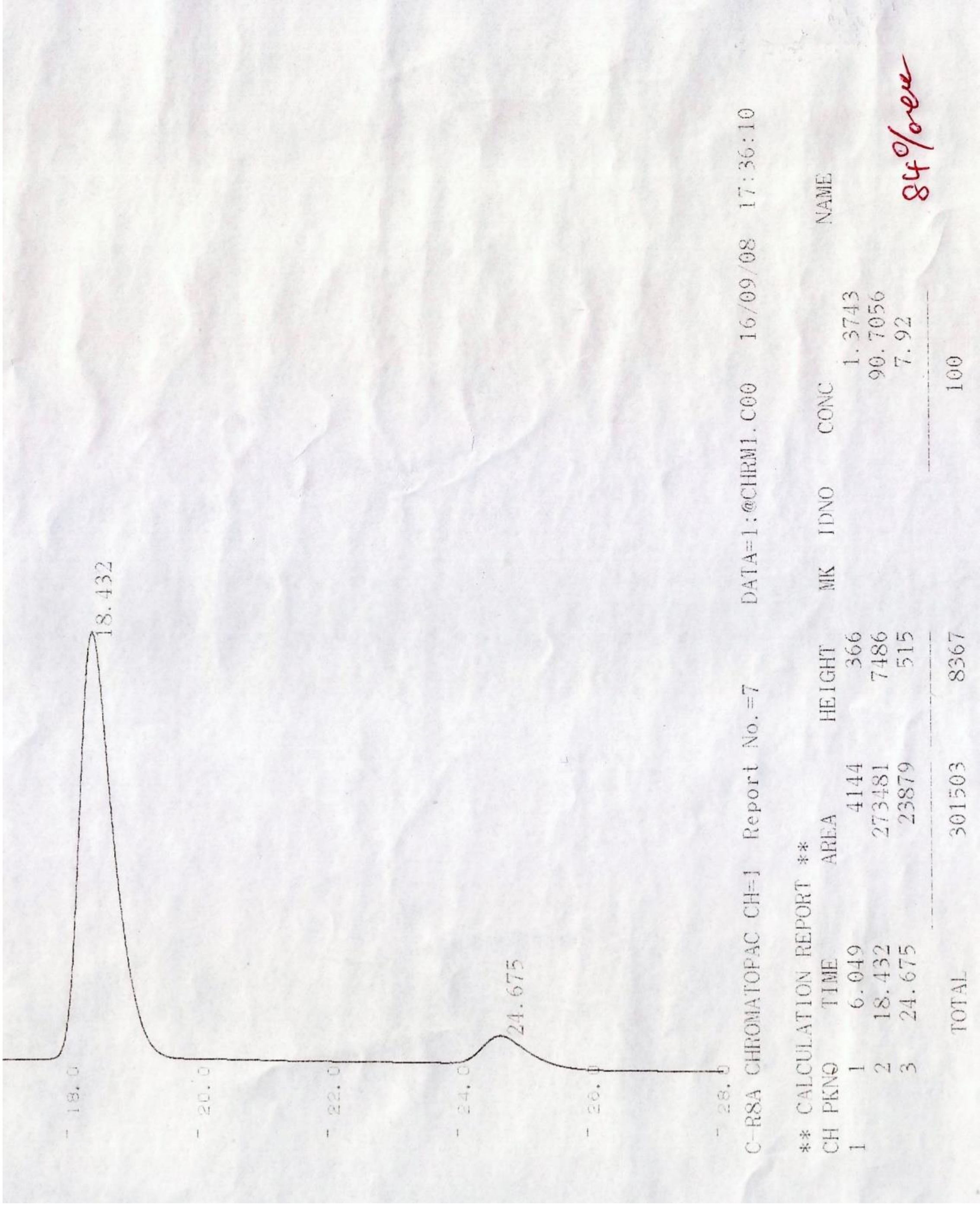

(*E*)-4,4,4-Trifluoro-3-(4-fluorophenyl)-1-phenylbut-2-en-1-ol ((*E*)-6c)

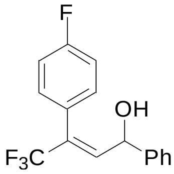

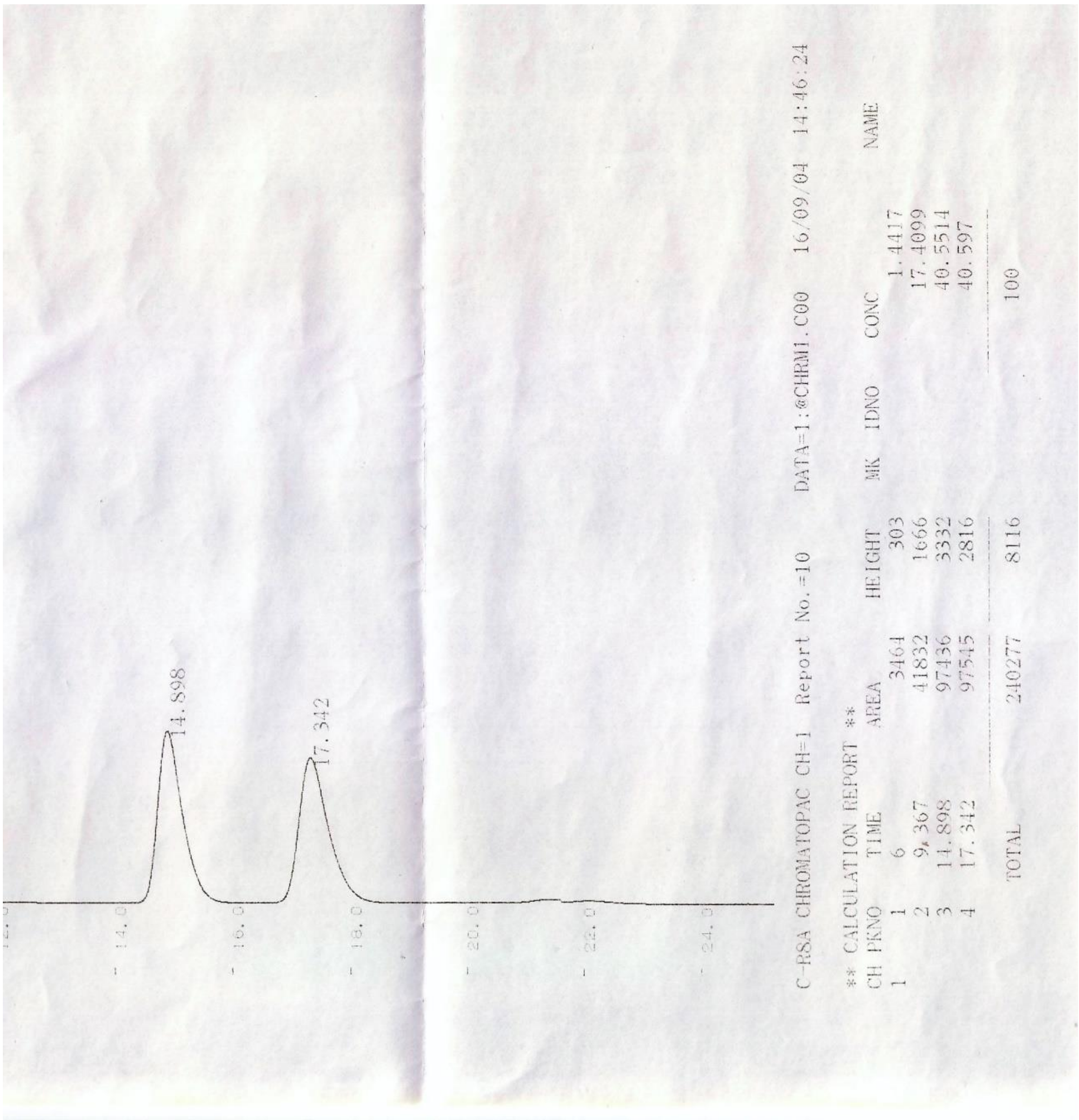

C-R8A CHROMATOPAC CH=1 Report No.=10 DATA=1:@CHRM1.C00 16/09/04 14:46:24

** CALCULATION REPORT **

CH	PKNO	TIME	AREA	HEIGHT	MK	IDNO	CONC	NAME
1	1	6	3464	303			1.4417	
	2	9.367	41832	1666			17.4099	
	3	14.898	97436	3332			40.5514	
	4	17.342	97545	2816			40.597	
		TOTAL	240277	8116			100	

(*R*,*E*)-4,4,4-Trifluoro-3-(4-fluorophenyl)-1-phenylbut-2-en-1-ol ((*R*,*E*)-6c)

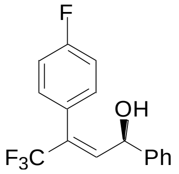

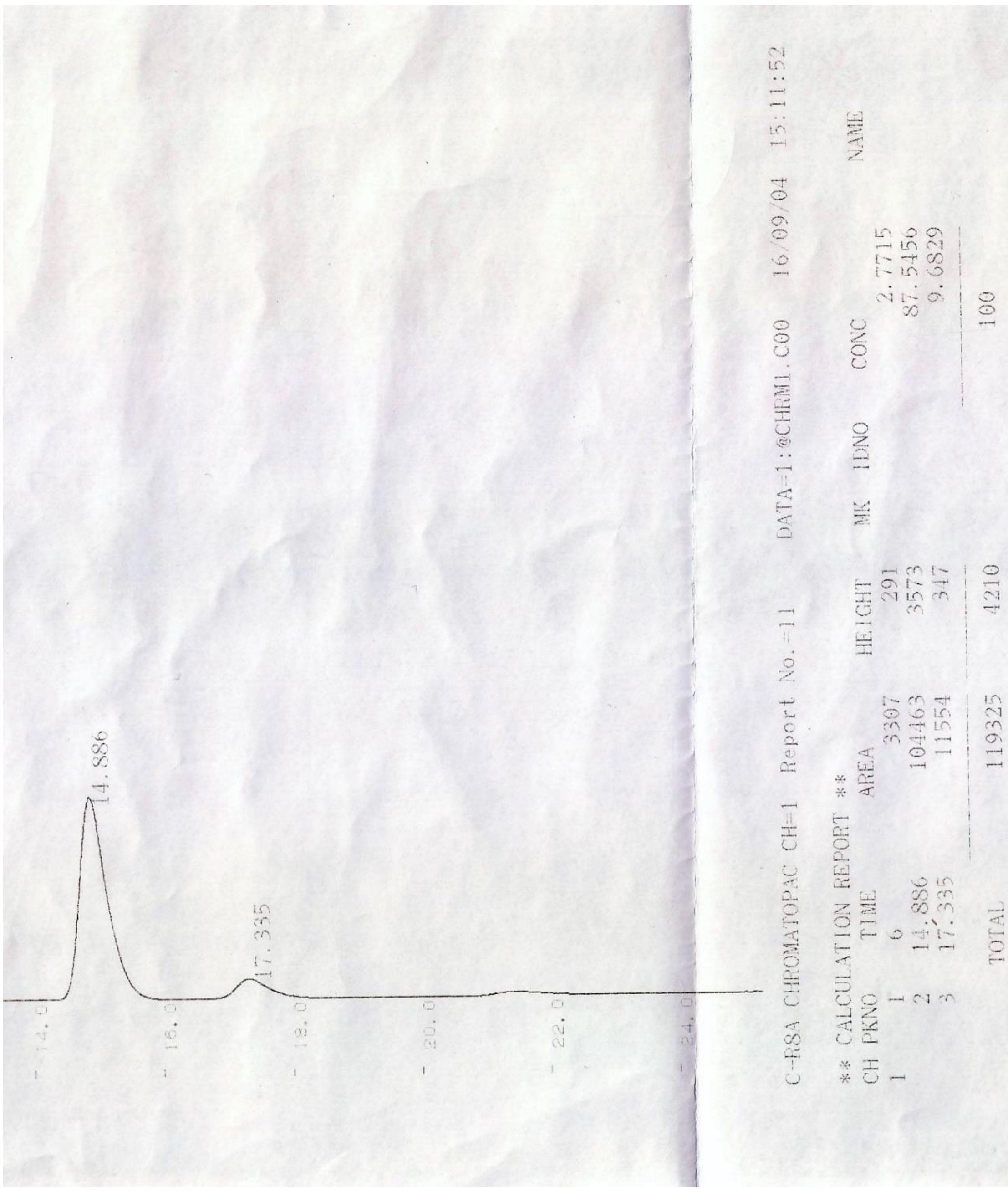

C-R8A CHROMATOPAC CH=1 Report No.=11 DATA=1:@CHRM1.C00 16/09/04 15:11:52

** CALCULATION REPORT **

CH	PKNO	TIME	AREA	HEIGHT	MK	IDNO	CONC	NAME
1	1	6	3307	291			2.7715	
	2	14.886	104463	3573			87.5456	
	3	17.335	11554	347			9.6829	
		TOTAL	119325	4210			100	

4,4,4-Trifluoro-1,3-diphenylbutan-1-one (7a)

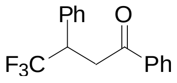

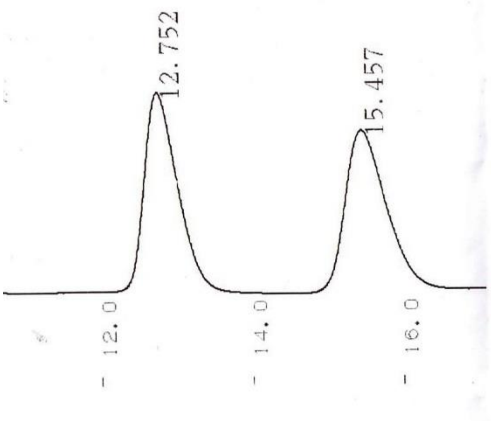

C-R8A CHROMATOPAC CH=1 Report No.=4 DATA=1:@CHRM1.C00 16/09/12 15:23:12

** CALCULATION REPORT **

CH	PKNO	TIME	AREA	HEIGHT	MK	IDNO	CONC	NAME
1	1	3.115	92694	3129			25.3451	
	2	5.807	88220	2369			24.1216	
	3	12.752	92500	3013			25.2918	
	4	15.457	92316	2438			25.2415	
		TOTAL	365730	10949			100	

(*R*)-4,4,4-Trifluoro-1,3-diphenylbutan-1-one ((*R*)-7a)

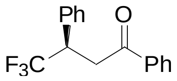

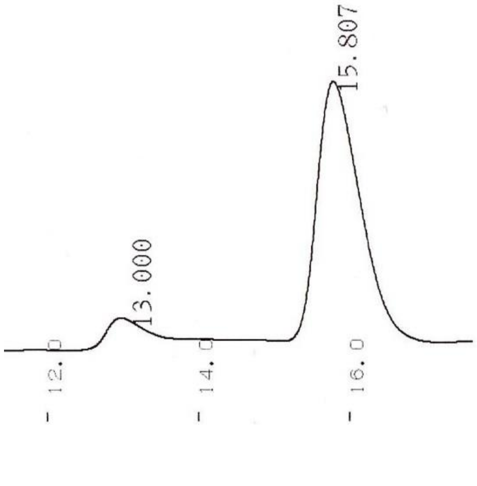

C–R8A CHROMATOPAC CH=1 Report No.=7 DATA=1:@CHRM1.C00 16/09/12 16:18:50

** CALCULATION REPORT **

CH	PKNO	TIME	AREA	HEIGHT	MK	IDNO	CONC	NAME
1	1	3.107	1431	181			1.6694	
	2	13	6164	201			7.1887	
	3	15.807	78147	1970			91.1418	
	TOTAL		85742	2352			100	

85%ee

4,4,4-Trifluoro-3-(4-methoxyphenyl)-1-phenylbutan-1-one (7b)

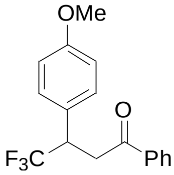

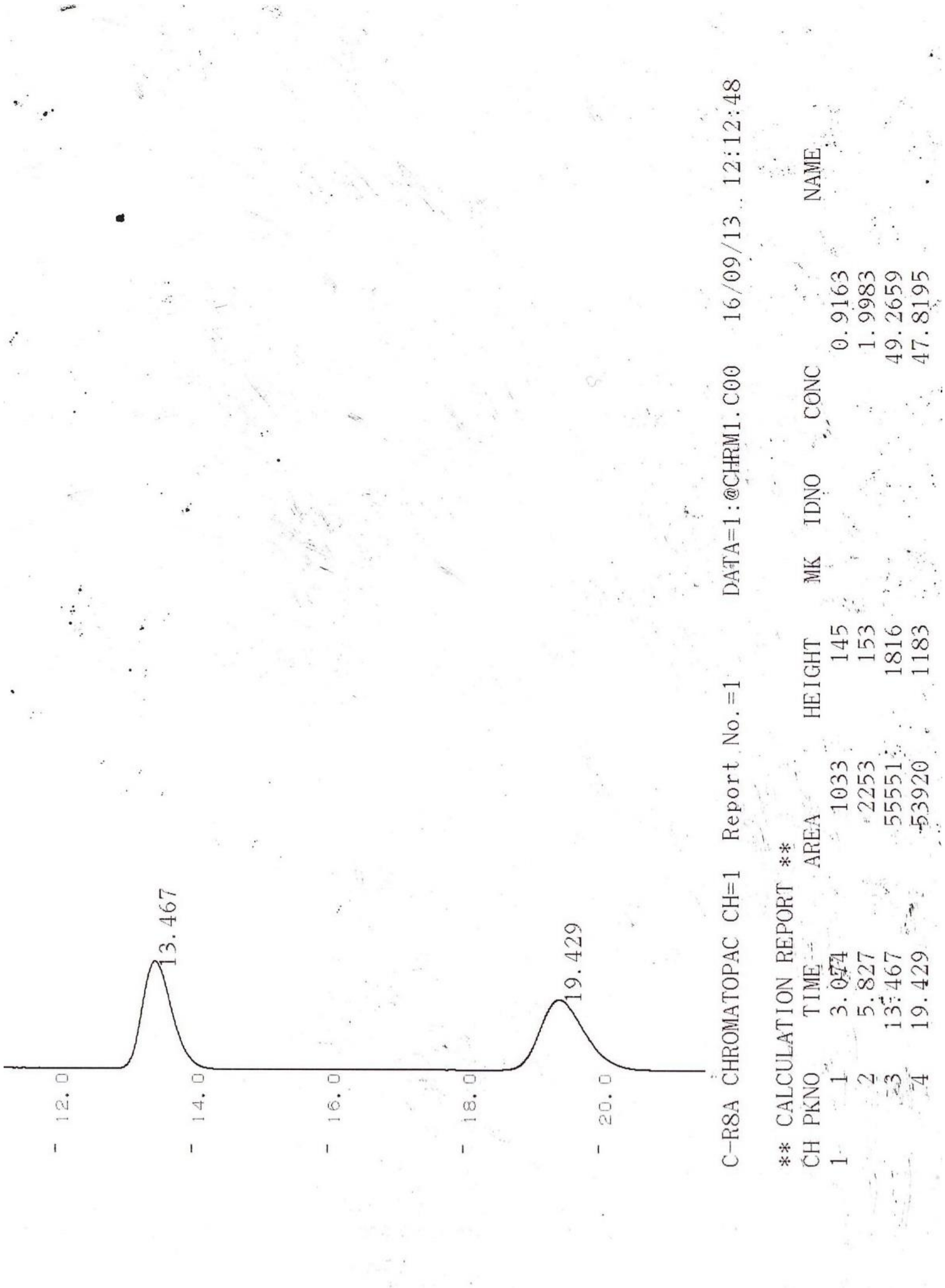

(*R*)-4,4,4-Trifluoro-3-(4-methoxyphenyl)-1-phenylbutan-1-one ((*R*)-7b)

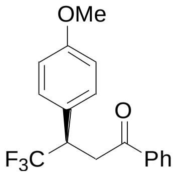

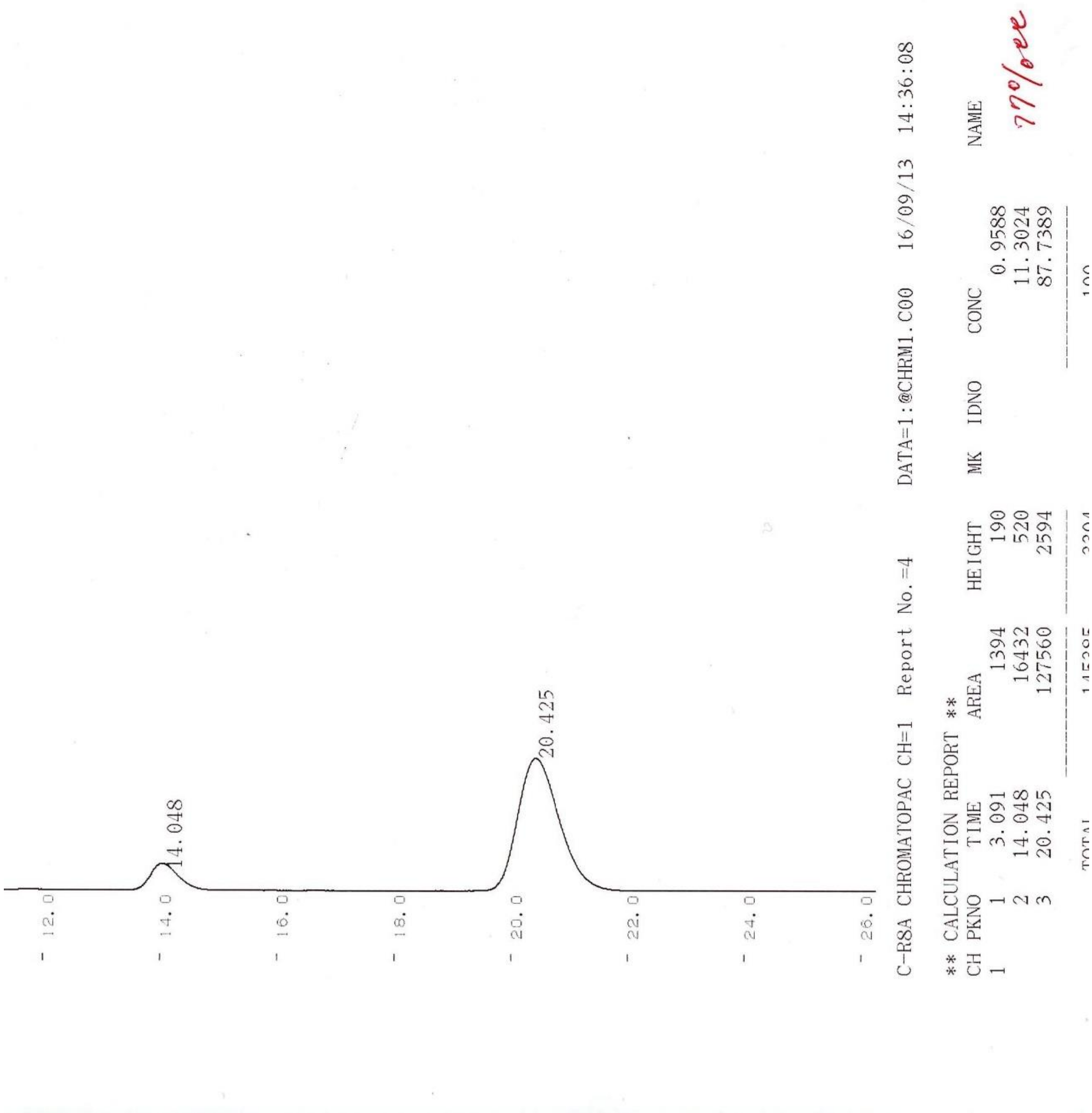

4,4,4-Trifluoro-3-(4-fluorophenyl)-1-phenylbutan-1-one (7c)

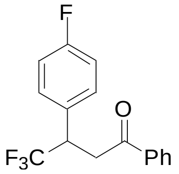

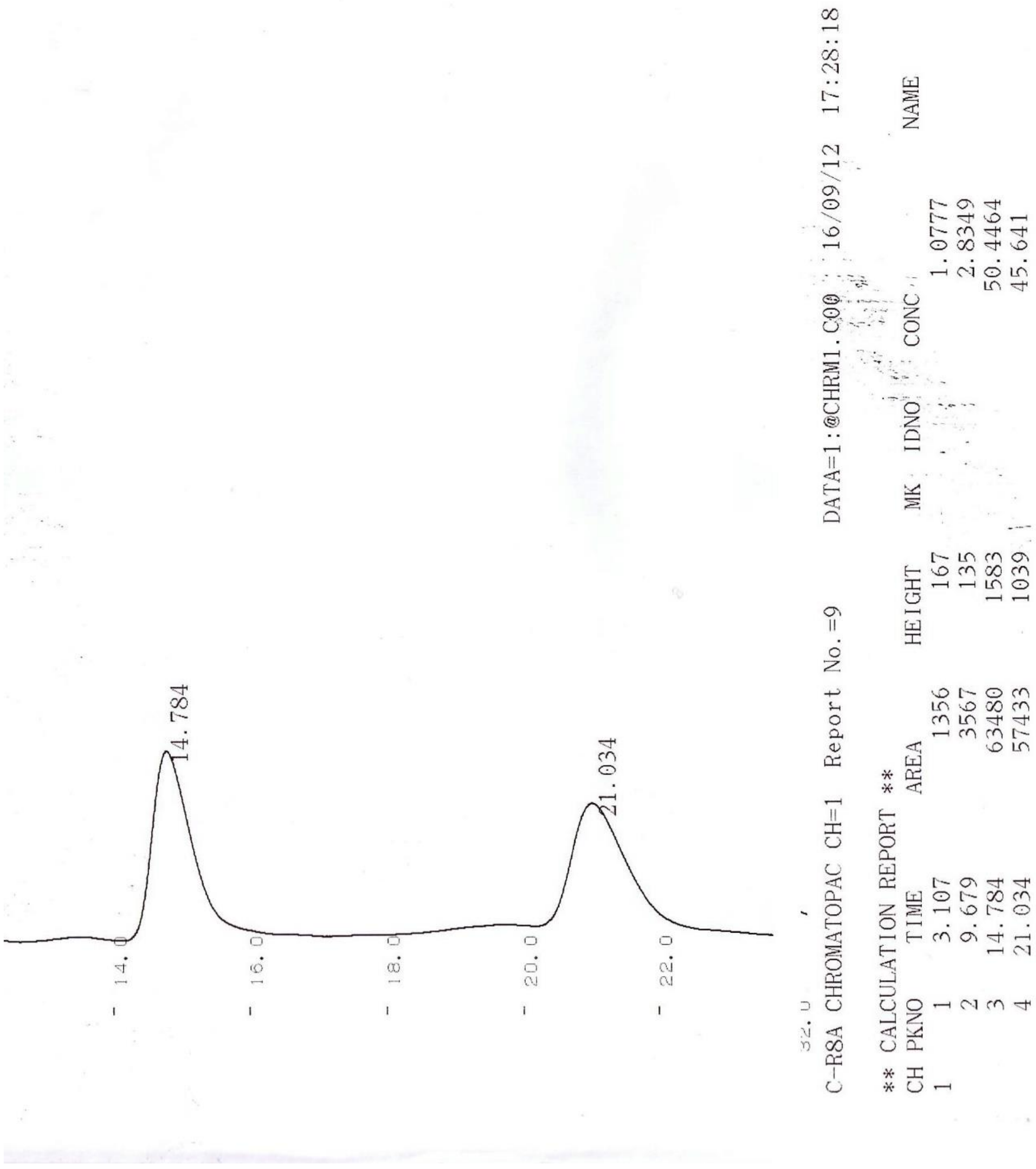

(*R*)-4,4,4-Trifluoro-3-(4-fluorophenyl)-1-phenylbutan-1-one ((*R*)-7c)

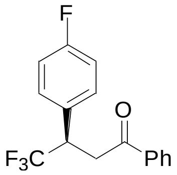

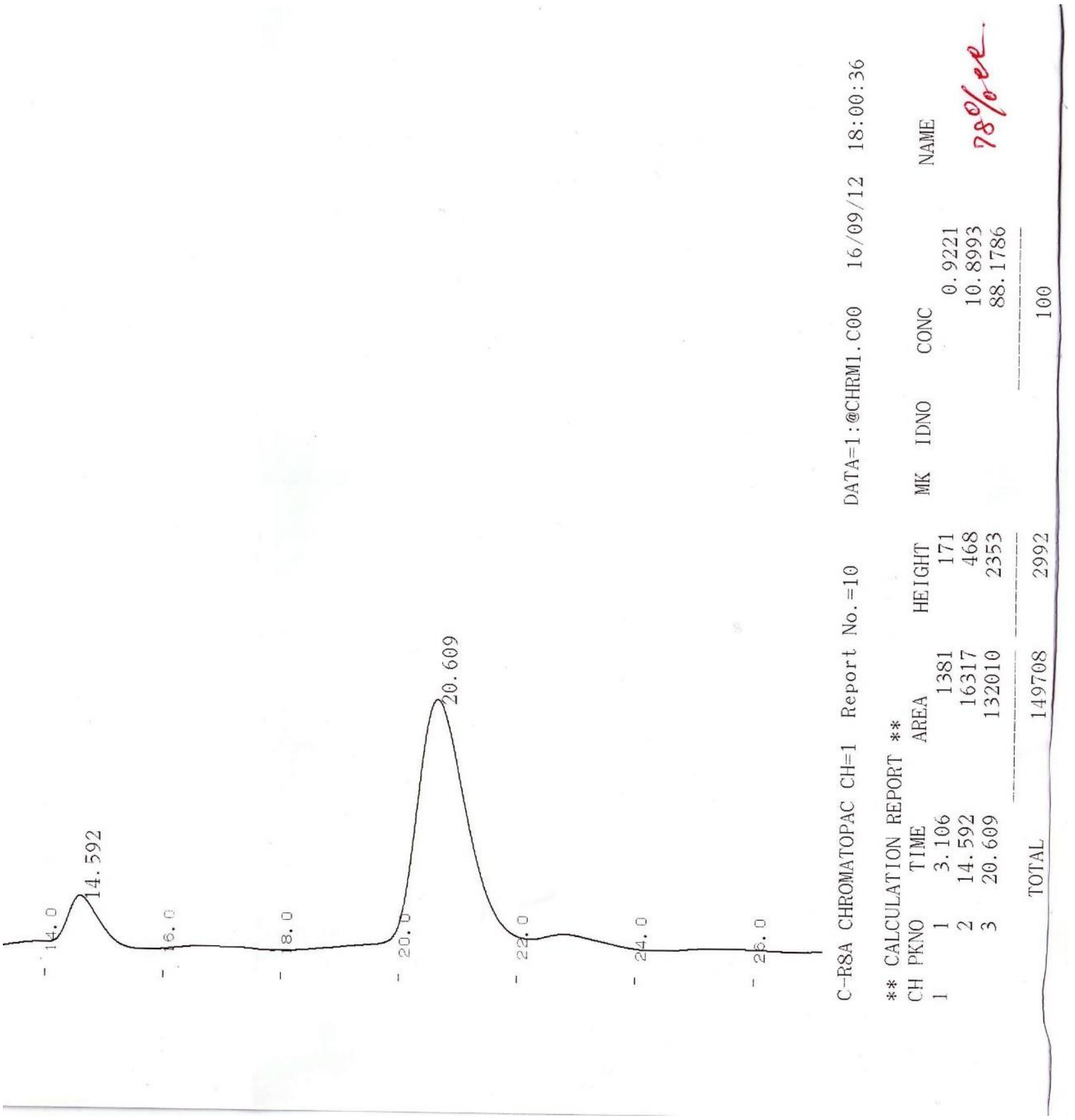

5. Computational Details

Full optimization for each compounds was carried out by Gaussian 09W [32] software using the B3LYP/6-311++G** level of theory, and the solvent effect was obtained as the single point calculation by the polarizable continuum model using the integral equation formalism variant (IEFPCM) or the conductor-like polarizable continuum model (CPCM). Frequency calculation was also performed for confirmation of optimized stationary points and transition state model by no and 1 negative frequency, respectively.

1Fa

E = –568.396577048, –568.403758070 (in THF)

C1	2.9357	1.2128	0.1820
H2	2.5416	2.0942	–0.3284
H3	4.0177	1.1852	0.0327
H4	2.7260	1.2941	1.2498
C5	2.3112	–0.0655	–0.3889
H6	2.5256	–0.1233	–1.4640
C7	0.8536	–0.0643	–0.2402
C8	–0.3414	–0.0310	–0.1254
C9	–1.7841	0.0049	0.0141
O10	2.7988	–1.2455	0.2629
H11	3.7377	–1.3282	0.0575
F12	–2.2004	–0.6445	1.1259
F13	–2.2512	1.2733	0.0994
F14	–2.4138	–0.5731	–1.0364

3F-Ca

E = –567.829356193, –567.901266570 (in THF)

C1	2.9283	–1.1541	0.0969
H2	2.3860	–2.0791	–0.1032
H3	3.3114	–1.1975	1.1247
H4	3.7997	–1.1136	–0.5702

C5	2.0370	0.0310	–0.1071
C6	0.7666	0.0165	–0.4666
C7	–0.4371	0.0285	–0.9527
C8	–1.5888	–0.0177	–0.0595
O9	2.7374	1.2429	0.1404
H10	2.1350	1.9730	–0.0425
F11	–2.4107	1.0816	–0.1988
F12	–2.4038	–1.1041	–0.3022
F13	–1.3819	–0.0831	1.3234

3F-Oa

E = –567.823099021, –567.899844205 (in THF)

C1	–3.0296	1.1395	–0.1747
H2	–2.6690	2.0245	0.3634
H3	–4.1135	1.0696	–0.0515
H4	–2.8107	1.2563	–1.2403
C5	–2.3976	–0.1775	0.3550
H6	–2.5905	–0.1734	1.4583
C7	–0.9082	–0.0452	0.2579
C8	0.2954	–0.0159	0.1362
C9	1.7225	0.0140	–0.0116
O10	–2.8355	–1.3003	–0.2678
F11	2.1634	–0.7087	–1.0795
F12	2.2100	1.2762	–0.1906
F13	2.3791	–0.4857	1.0741

1Fb

E = –760.176083913, –760.183467700 (in THF)

C1	–0.6070	1.4656	–0.2595
H2	–0.6673	1.9307	–1.2515
C3	0.7024	0.8123	–0.1692
C4	1.7701	0.2645	–0.1238

C5	3.0535	–0.4089	–0.0705
O6	–0.6404	2.4774	0.7560
H7	–1.5021	2.9101	0.7067
C8	–1.7449	0.4597	–0.1316
C9	–2.6171	0.2536	–1.2018
C10	–1.9337	–0.2492	1.0602
C11	–3.6706	–0.6546	–1.0857
H12	–2.4761	0.8020	–2.1274
C13	–2.9870	–1.1512	1.1767
H14	–1.2594	–0.0907	1.8942
C15	–3.8567	–1.3569	0.1030
H16	–4.3439	–0.8085	–1.9212
H17	–3.1290	–1.6969	2.1025
H18	–4.6755	–2.0612	0.1954
F19	4.0838	0.4394	–0.2969
F20	3.2763	–0.9827	1.1349
F21	3.1423	–1.3916	–0.9973

3F-Cb

E = –759.630921556, –759.695201006 (in THF)

C1	0.4261	1.0959	–0.1989
C2	–0.7851	0.6009	–0.4672
C3	–1.9199	0.1686	–0.8740
C4	–2.9846	–0.2983	0.0004
O5	0.6060	2.4896	–0.0080
H6	–0.2319	2.9195	–0.2103
C7	1.6514	0.3221	–0.0861
C8	1.6498	–1.0851	–0.2239
C9	2.8913	0.9505	0.1589
C10	2.8251	–1.8173	–0.1307
H11	0.7106	–1.5955	–0.4061
C12	4.0665	0.2061	0.2547
H13	2.9247	2.0265	0.2694
C14	4.0490	–1.1808	0.1109

H15	2.7895	–2.8962	–0.2441
H16	5.0047	0.7187	0.4431
H17	4.9646	–1.7565	0.1861
F18	–2.7829	–0.2435	1.3776
F19	–4.1554	0.4019	–0.1789
F20	–3.3093	–1.6149	–0.2252

3F-Ob

E = –759.610861467, –759.682660875 (in THF)

C1	–0.6196	1.3903	–0.7080
H2	–0.6589	1.4164	–1.8275
C3	0.6748	0.6859	–0.4194
C4	1.7581	0.2186	–0.1532
C5	3.0412	–0.3431	0.1622
O6	–0.6503	2.6075	–0.1320
C7	–1.7718	0.4199	–0.3155
C8	–1.9424	–0.8136	–0.9538
C9	–2.6784	0.7984	0.6726
C10	–2.9944	–1.6582	–0.6026
H11	–1.2471	–1.1163	–1.7323
C12	–3.7342	–0.0444	1.0298
H13	–2.5369	1.7644	1.1427
C14	–3.8956	–1.2754	0.3948
H15	–3.1148	–2.6119	–1.1064
H16	–4.4325	0.2616	1.8024
H17	–4.7157	–1.9304	0.6691
F18	4.0791	0.4404	–0.2456
F19	3.2213	–0.5381	1.4982
F20	3.2422	–1.5594	–0.4229

1Ha

E = –270.587474540, –270.594534928 (in THF)

C1	–1.7619	1.2009	–0.1789
H2	–1.3771	2.0913	0.3238
H3	–2.8426	1.1575	–0.0168
H4	–1.5681	1.2879	–1.2499
C5	–1.0959	–0.0610	0.3815
H6	–1.3062	–0.1236	1.4575
C7	0.3570	–0.0458	0.2008
C8	1.5532	–0.0049	0.0599
C9	3.0008	0.0365	–0.1136
O10	–1.6057	–1.2500	–0.2581
H11	–2.5487	–1.3048	–0.0623
H12	3.3178	0.9861	–0.5534
H13	3.5166	–0.0818	0.8432
H14	3.3345	–0.7671	–0.7758

3H-Ca

E = –269.970093212, –270.047887418 (in THF)

C1	1.8936	–1.1257	0.0959
H2	1.3690	–2.0748	–0.0252
H3	2.4049	–1.1433	1.0689
H4	2.6815	–1.0710	–0.6709
C5	0.9332	0.0178	–0.0176
C6	–0.3658	–0.0230	–0.2860
C7	–1.6072	–0.0359	–0.6623
C8	–2.7424	–0.0842	0.3439
O9	1.6367	1.2654	0.1269
H10	0.9896	1.9635	–0.0238
H11	–2.4232	–0.1091	1.4030
H12	–3.4069	0.7803	0.2191
H13	–3.3765	–0.9629	0.1702

3H-Oa

E = –269.987804539, –270.078085013 (in THF)

C1	–1.8318	1.1392	–0.1656

H2	–1.4530	2.0257	0.3590
H3	–2.9136	1.0766	–0.0132
H4	–1.6427	1.2569	–1.2378
C5	–1.1783	–0.1768	0.3436
H6	–1.3556	–0.1731	1.4505
C7	0.3110	–0.0432	0.2062
C8	1.5127	0.0112	0.0653
C9	2.9634	0.0624	–0.1129
O10	–1.6724	–1.2961	–0.2615
H11	3.4810	0.1938	0.8427
H12	3.3411	–0.8599	–0.5660
H13	3.2591	0.8921	–0.7629

1Hb

E = –462.367245869, –462.374910305 (in THF)

C1	0.7215	1.1462	–0.1593
H2	0.7228	1.7432	–1.0802
C3	1.8780	0.2494	–0.1953
C4	2.8271	–0.4907	–0.2556
C5	3.9731	–1.3894	–0.3213
O6	0.8883	2.0306	0.9701
H7	0.1270	2.6240	0.9878
H8	4.5844	–1.1842	–1.2043
H9	4.6082	–1.2728	0.5611
H10	3.6513	–2.4334	–0.3677
C11	–0.6018	0.3918	–0.0930
C12	–1.5544	0.5612	–1.0995
C13	–0.8866	–0.4588	0.9816
C14	–2.7764	–0.1119	–1.0396
H15	–1.3418	1.2199	–1.9355
C16	–2.1061	–1.1265	1.0453
H17	–0.1507	–0.5953	1.7661
C18	–3.0542	–0.9562	0.0329
H19	–3.5076	0.0265	–1.8281
H20	–2.3181	–1.7833	1.8816

H21	–4.0024	–1.4794	0.0827

3H-Cb

E = –461.781456797, –461.849393931 (in THF)

C1	–0.7357	0.8308	–0.0329
C2	–1.9142	0.1603	–0.1598
C3	–3.0016	–0.4108	–0.4295
C4	–4.1424	–1.0465	0.2873
O5	–0.7629	2.2648	0.0427
H6	–1.6305	2.5372	–0.2737
H7	–4.0458	–1.0333	1.3850
H8	–5.0861	–0.5512	0.0335
H9	–4.2642	–2.0907	–0.0209
C10	0.5713	0.2579	–0.0092
C11	0.7784	–1.1524	–0.0664
C12	1.7441	1.0634	0.0738
C13	2.0505	–1.7011	–0.0495
H14	–0.0876	–1.8032	–0.1255
C15	3.0144	0.4954	0.0927
H16	1.6359	2.1394	0.1186
C17	3.1943	–0.8899	0.0326
H18	2.1594	–2.7811	–0.0987
H19	3.8810	1.1482	0.1553
H20	4.1867	–1.3260	0.0493

3H-Ob

E = –461.778352466, –461.861828646 (in THF)

C1	–0.7448	1.0495	0.6889
H2	–0.7716	0.9682	1.8065
C3	–1.8626	0.1568	0.2247
C4	–2.8055	–0.4881	–0.1759
C5	–3.9463	–1.2587	–0.6678
O6	–0.8706	2.3236	0.2477
H7	–4.5995	–1.5752	0.1516

H8	–4.5531	–0.6685	–1.3620
H9	–3.6233	–2.1598	–1.1986
C10	0.5932	0.3488	0.3087
C11	0.9854	–0.8532	0.9102
C12	1.4441	0.9326	–0.6293
C13	2.1936	–1.4633	0.5760
H14	0.3364	–1.3156	1.6494
C15	2.6543	0.3243	–0.9740
H16	1.1362	1.8743	–1.0699
C17	3.0348	–0.8759	–0.3734
H18	2.4825	–2.3931	1.0560
H19	3.3041	0.7907	–1.7082
H20	3.9764	–1.3468	–0.6353

2Fa

E = –569.662705667,   –569.669891067 (in THF)

C1	2.8704	1.2120	0.1179
H2	2.3726	2.1072	–0.2624
H3	3.8998	1.2151	–0.2527
H4	2.8938	1.2586	1.2096
C5	2.1445	–0.0506	–0.3538
H6	2.1145	–0.0655	–1.4499
C7	–1.7288	0.0068	–0.0186
O8	2.7962	–1.2397	0.1242
H9	3.7286	–1.1928	–0.1169
F10	–1.7824	–0.1629	1.3197
F11	–2.3820	1.1702	–0.2921
F12	–2.4845	–0.9810	–0.5702
C13	–0.3448	0.0123	–0.5705
H14	–0.2997	0.1269	–1.6484
C15	0.7429	–0.1041	0.1835
H16	0.6560	–0.2279	1.2594

4F-Oa

E = –569.080892049, –569.158580004 (in THF)

C1	–3.0643	–1.0846	0.0790
H2	–2.6676	–2.0223	–0.3322
H3	–4.0901	–0.9537	–0.2805
H4	–3.0975	–1.1659	1.1715
C5	–2.2240	0.1483	–0.3352
H6	–2.1605	0.1164	–1.4508
C7	1.6740	–0.0234	–0.0221
O8	–2.7153	1.3341	0.1409
F9	1.7373	–0.0986	1.3290
F10	2.4117	–1.0782	–0.4963
F11	2.3981	1.0877	–0.3724
C12	0.3000	–0.0059	–0.5690
H13	0.2596	0.0359	–1.6533
C14	–0.8063	–0.0091	0.1821
H15	–0.7208	–0.0326	1.2670

2Fb

E = –761.442998032, –761.450752358 (in THF)

C1	–0.4857	1.2356	–0.6039
H2	–0.3424	1.2246	–1.690
C3	2.9648	–0.4257	0.0766
O4	–0.6198	2.5869	–0.1408
H5	–1.4500	2.9417	–0.4815
C6	–1.7019	0.3831	–0.2654
C7	–2.2372	–0.4846	–1.2215
C8	–2.2869	0.4426	1.0052
C9	–3.3381	–1.2841	–0.9150
H10	–1.7947	–0.5334	–2.2112
C11	–3.3889	–0.3532	1.3108
H12	–1.8857	1.1198	1.7507
C13	–3.9160	–1.2201	0.3523

H14	–3.7467	–1.9500	–1.6666
H15	–3.8363	–0.2974	2.2968
H16	–4.7738	–1.8385	0.5911
F17	4.1081	0.1664	–0.3629
F18	2.9497	–0.2957	1.4203
F19	3.1093	–1.7536	–0.1870
C20	1.7627	0.1417	–0.5968
H21	1.7833	0.0627	–1.6785
C22	0.7493	0.6915	0.0635
H23	0.7678	0.7593	1.1470

4F-Cb

E = –760.881059270, –760.943671947 (in THF)

C1	–0.5236	1.1758	–0.1071
C2	2.9668	–0.3555	0.0265
O3	–0.7837	2.5776	–0.0735
H4	–1.3230	2.8091	–0.8395
C5	–1.6855	0.3241	–0.0354
C6	–1.6363	–1.0821	–0.2454
C7	–2.9748	0.8718	0.2149
C8	–2.7814	–1.8681	–0.1891
H9	–0.6976	–1.5570	–0.4885
C10	–4.1122	0.0745	0.2725
H11	–3.0689	1.9365	0.3876
C12	–4.0347	–1.3071	0.0753
H13	–2.6944	–2.9365	–0.3629
H14	–5.0724	0.5382	0.4783
H15	–4.9228	–1.9270	0.1221
F16	3.5367	–1.5409	–0.3868
F17	3.6017	0.6431	–0.6719
F18	3.5394	–0.2055	1.3319
C19	1.5318	–0.3520	–0.0563
H20	1.0822	–1.3077	0.1664
C21	0.8203	0.8541	–0.1388

H22	1.4435	1.7413	–0.2369

4F-Ob

E = –760.870749534, –760.943160273 (in THF)

C1	–0.5091	1.2123	–0.6736
H2	–0.3539	1.0430	–1.7671
C3	2.9994	–0.3344	0.1313
O4	–0.5851	2.5248	–0.3305
C5	–1.7512	0.3605	–0.3131
C6	–1.9150	–0.9419	–0.8033
C7	–2.7418	0.8885	0.5163
C8	–3.0370	–1.6990	–0.4693
H9	–1.1567	–1.3661	–1.4561
C10	–3.8678	0.1349	0.8574
H11	–2.6078	1.9036	0.8721
C12	–4.0200	–1.1620	0.3668
H13	–3.1486	–2.7051	–0.8611
H14	–4.6294	0.5613	1.5030
H15	–4.8951	–1.7480	0.6272
F16	4.1286	0.3463	–0.2423
F17	2.9281	–0.2329	1.4793
F18	3.2842	–1.6494	–0.1361
C19	1.7901	0.1347	–0.5808
H20	1.8653	0.0737	–1.6622
C21	0.6982	0.6059	0.0293
H22	0.6635	0.6648	1.1147

2Ha

E = –271.839948035, –271.845389714 (in THF)

C1	–1.8333	1.1797	–0.0917
H2	–1.3610	2.0914	0.2831
H3	–2.8429	1.1272	0.3288

H4	–1.9154	1.2516	–1.1799
C5	–1.0136	–0.0498	0.3063
H6	–0.9263	–0.0873	1.3997
C7	2.8763	0.0446	–0.1610
O8	–1.6559	–1.2613	–0.1544
H9	–2.5768	–1.2421	0.1307
C10	1.4922	–0.0052	0.4150
H11	1.4257	–0.0165	1.5027
C12	0.3630	–0.0322	–0.2923
H13	0.3999	–0.0286	–1.3814
H14	3.4689	–0.8147	0.1716
H15	3.4095	0.9405	0.1758
H16	2.8584	0.0471	–1.2533

4H-Ca

E = –271.204717656, –271.279129958 (in THF)

C1	1.5202	1.3037	0.0259
H2	0.9167	2.0515	–0.5004
H3	2.5628	1.4616	–0.2729
H4	1.4533	1.5539	1.1109
C5	1.0452	–0.0869	–0.2906
C6	–2.8293	–0.1584	0.0093
O7	1.9789	–1.1061	0.2033
H8	2.6154	–1.2634	–0.4971
C9	–1.4198	0.3754	0.0949
H10	–1.3116	1.4579	0.0378
C11	–0.3027	–0.4275	–0.0766
H12	–0.4856	–1.5064	–0.0465
H13	–3.3073	–0.0184	–0.9793
H14	–3.5065	0.3111	0.7359
H15	–2.8500	–1.2374	0.2079

4H-Oa

E = –271.235872940, –271.323498797 (in THF)

C1	–1.9163	1.1079	–0.0882
H2	–1.4671	2.0344	0.2943
H3	–2.9296	1.0182	0.3185
H4	–1.9990	1.1850	–1.1794
C5	–1.0958	–0.1547	0.2895
H6	–0.9813	–0.1123	1.4034
C7	2.8362	0.0707	–0.1673
O8	–1.6895	–1.3165	–0.1371
C9	1.4509	0.0265	0.4202
H10	1.3949	0.0178	1.5100
C11	0.3112	–0.0207	–0.2753
H12	0.3566	–0.0343	–1.3675
H13	3.4376	–0.7875	0.1574
H14	3.3819	0.9688	0.1473
H15	2.8043	0.0630	–1.2605

2Hb

E = –463.620454871, –463.626716310 (in THF)

C1	0.7565	0.7745	–0.6212
H2	0.9697	0.5098	–1.6637
C3	3.8659	–1.2335	0.6643
O4	0.8472	2.2057	–0.4617
H5	0.1162	2.6082	–0.9462
C6	–0.6336	0.2596	–0.2712
C7	–1.2643	–0.6762	–1.0964
C8	–1.2914	0.6910	0.8875
C9	–2.5247	–1.1780	–0.7711
H10	–0.7675	–1.0138	–2.0005
C11	–2.5515	0.1932	1.2129
H12	–0.8172	1.4243	1.5301
C13	–3.1716	–0.7447	0.3855
H14	–3.0020	–1.9011	–1.4231
H15	–3.0504	0.5367	2.1125
H16	–4.1525	–1.1310	0.6388
C17	2.8124	–0.5951	–0.1907

H18	2.8820	–0.7857	–1.2611
C19	1.8156	0.1675	0.2581
H20	1.7198	0.3777	1.3220
H21	3.8468	–2.3239	0.5592
H22	4.8663	–0.9075	0.3595
H23	3.7314	–0.9890	1.7204

4H-Cb

E = –463.024162178, –463.090478358 (in THF)

C1	–0.6767	0.9547	0.0143
C2	–4.0691	–1.0038	–0.0362
O3	–0.6333	2.3908	–0.0115
H4	–0.1987	2.6976	0.7939
C5	0.5702	0.2937	–0.0106
C6	0.7241	–1.1351	0.0694
C7	1.8071	1.0283	–0.0853
C8	1.9726	–1.7405	0.0768
H9	–0.1500	–1.7639	0.1474
C10	3.0429	0.3999	–0.0812
H11	1.7703	2.1078	–0.1680
C12	3.1593	–0.9965	0.0001
H13	2.0237	–2.8246	0.1455
H14	3.9401	1.0107	–0.1471
H15	4.1289	–1.4815	0.0015
C16	–2.5758	–0.7951	–0.0643
H17	–1.9803	–1.6908	–0.1946
C18	–2.0045	0.4493	0.0299
H19	–2.7263	1.2645	0.1224
H20	–4.4474	–1.4902	–0.9479
H21	–4.3985	–1.6373	0.8011
H22	–4.5964	–0.0482	0.0595

4H-Ob

E = –463.028204220, –463.108999137 (in THF)

C1	0.7338	0.9510	–0.5387
H2	0.9427	0.6881	–1.6074
C3	3.9412	–1.0824	0.6013
O4	0.7691	2.2920	–0.3009
C5	–0.6422	0.2911	–0.2515
C6	–0.9102	–1.0419	–0.5940
C7	–1.6603	1.0330	0.3508
C8	–2.1546	–1.6169	–0.3393
H9	–0.1324	–1.6358	–1.0663
C10	–2.9089	0.4631	0.6149
H11	–1.4427	2.0672	0.5929
C12	–3.1628	–0.8646	0.2712
H13	–2.3413	–2.6500	–0.6163
H14	–3.6868	1.0576	1.0849
H15	–4.1323	–1.3089	0.4710
C16	2.8460	–0.4505	–0.2149
H17	2.9390	–0.5485	–1.2975
C18	1.7996	0.2192	0.2752
H19	1.7028	0.3454	1.3555
H20	4.0137	–2.1604	0.4127
H21	4.9220	–0.6570	0.3553
H22	3.7719	–0.9395	1.6722

DABCO

E = –345.421363954l, –345.424760724 (in toluene)

N1	0.0000	0.0000	1.2859
C2	–0.0001	1.3852	0.7820
H3	–0.8819	1.8937	1.1831
H4	0.8815	1.8939	1.1833
C5	–1.1996	–0.6927	0.7820
H6	–1.1990	–1.7106	1.1831
H7	–2.0809	–0.1836	1.1833
C8	1.1997	–0.6925	0.7820
H9	2.0809	–0.1830	1.1831
H10	1.1994	–1.7103	1.1833

C11	1.1996	–0.6927	–0.7820
H12	2.0809	–0.1836	–1.1833
H13	1.1990	–1.7106	–1.1831
C14	–1.1997	–0.6925	–0.7820
H15	–1.1994	–1.7103	–1.1833
H16	–2.0809	–0.1831	–1.1831
N17	0.0000	0.0000	–1.2859
C18	0.0001	1.3852	–0.7820
H19	0.8819	1.8937	–1.1831
H20	–0.8815	1.8939	–1.1833

Substrate ***(R,E)*-6h** (R^1: Ph, R^2: Me in Table 3)
E = –800.769760546, –800.774966608 (in toluene)

C1	–1.6574	0.1078	0.4576
C2	–0.6256	0.7096	–0.1369
H3	–0.6306	0.8235	–1.2156
C4	–2.7876	–0.4060	–0.3984
C5	0.6076	1.2629	0.5252
H6	0.4804	1.2882	1.6122
O7	0.7494	2.5988	0.0245
H8	1.5983	2.9444	0.3231
F9	–3.9654	0.1888	–0.0720
F10	–2.6098	–0.2168	–1.7200
F11	–2.9761	–1.7406	–0.2144
C12	–1.8460	–0.1503	1.9296
H13	–2.8472	0.1507	2.2478
H14	–1.7403	–1.2159	2.1546
H15	–1.1214	0.3970	2.5303
C16	1.8221	0.3982	0.2080
C17	2.3529	–0.4565	1.1777
C18	2.4116	0.4346	–1.0611
C19	3.4500	–1.2659	0.8869
H20	1.9086	–0.4888	2.1677
C21	3.5098	–0.3708	–1.3511
H22	2.0136	1.1032	–1.8160

C23	4.0304	–1.2251	–0.3791
H24	3.8537	–1.9221	1.6498
H25	3.9595	–0.3329	–2.3371
H26	4.8854	–1.8517	–0.6064

Transition state **TS-8h**

E = –1146.14793316, –1146.16029984 (in toluene)

C1	1.0036	–2.0077	0.5781
C2	–0.2749	–1.5964	0.1851
H3	–0.5089	–1.8005	–0.8550
C4	1.9127	–2.5133	–0.4449
C5	–1.2822	–0.8945	0.8393
H6	0.4622	0.2805	0.1976
O7	–1.0116	–0.3707	2.1312
H8	–1.6030	–0.7824	2.7736
F9	2.5839	–3.6513	–0.0991
F10	1.3601	–2.7420	–1.6649
F11	2.9869	–1.6108	–0.7374
N12	0.8813	1.2228	0.0373
C13	–0.1338	2.2699	0.4114
H14	–1.0211	2.0839	–0.1929
H15	–0.3982	2.0912	1.4525
C16	1.2354	1.3351	–1.4216
H17	1.9355	0.5292	–1.6396
H18	0.3186	1.1636	–1.9859
C19	2.1205	1.3687	0.8794
H20	1.8150	1.2316	1.9160
H21	2.7872	0.5538	0.6020
C22	–2.5773	–0.5666	0.2756
C23	–3.4439	0.3474	0.9279
C24	–3.0583	–1.1305	–0.9363
C25	–4.6826	0.6906	0.3920
H26	–3.1304	0.7995	1.8610
C27	–4.2877	–0.7707	–1.4676
H28	–2.4706	–1.8805	–1.4522

C29	–5.1185	0.1467	–0.8146
H30	–5.3136	1.3932	0.9276
H31	–4.6150	–1.2293	–2.3954
H32	–6.0842	0.4121	–1.2282
C33	2.7090	2.7811	0.5917
H34	2.7628	3.3708	1.5088
H35	3.7217	2.7002	0.1925
C36	1.8339	2.7552	–1.6430
H37	2.8469	2.6845	–2.0429
H38	1.2348	3.3191	–2.3603
N39	1.8800	3.5065	–0.3813
C40	0.5184	3.6587	0.1515
H41	0.5716	4.2387	1.0748
H42	–0.0724	4.2335	–0.5641
C43	1.5412	–2.0737	1.9924
H44	1.0138	–1.3775	2.6421
H45	1.4273	–3.0764	2.4256
H46	2.6113	–1.8410	2.0342

Product **(*R*)-7h** (R[1]: Ph, R[2]: Me in Table 3)
E = –800.803754065, –800.806147466 (in toluene)

C1	–1.8912	0.5690	0.0512
C2	–0.5463	–0.1727	0.0347
H3	–0.5307	–0.9256	–0.7613
C4	–3.0294	–0.4060	–0.1956
C5	0.6414	0.7570	–0.2065
H6	–0.4035	–0.7170	0.9729
O7	0.4693	1.9213	–0.5149
H8	–1.9123	1.2644	–0.7923
F9	–4.2311	0.2135	–0.2209
F10	–2.9004	–1.0496	–1.3836
F11	–3.1059	–1.3701	0.7582
C12	–2.1147	1.3547	1.3512
H13	–3.0639	1.8920	1.3295
H14	–2.1167	0.6878	2.2174

H15	–1.3158	2.0859	1.4742
C16	2.0248	0.1938	–0.0787
C17	2.2731	–1.1491	0.2370
C18	3.1111	1.0549	–0.2930
C19	3.5801	–1.6200	0.3359
H20	1.4538	–1.8372	0.4045
C21	4.4142	0.5847	–0.1915
H22	2.9078	2.0897	–0.5393
C23	4.6514	–0.7546	0.1233
H24	3.7613	–2.6608	0.5788
H25	5.2469	1.2586	–0.3579
H26	5.6685	–1.1220	0.2020

DABCO-H^+

E = –345.802987802, –345.851815869 (in toluene)

N1	1.2261	0.0032	–0.0018
C2	0.7392	–0.7534	–1.2257
H3	1.1552	–0.2512	–2.0994
H4	1.1558	–1.7592	–1.1656
C5	0.7358	1.4401	–0.0418
H6	1.1535	1.9454	0.8294
H7	1.1491	1.8939	–0.9429
C8	0.7428	–0.6827	1.2644
H9	1.1616	–1.6893	1.2619
H10	1.1591	–0.1298	2.1068
C11	–0.8170	–0.6629	1.2215
H12	–1.2098	–1.6798	1.2473
H13	–1.2140	–0.1327	2.0877
C14	–0.8228	1.3859	–0.0364
H15	–1.2184	1.9159	0.8306
H16	–1.2226	1.8687	–0.9287
N17	–1.2872	–0.0023	0.0019
C18	–0.8205	–0.7280	–1.1816
H19	–1.2155	–1.7439	–1.1526
H20	–1.2180	–0.2437	–2.0741

H21	2.2460	0.0055	–0.0033

6. References

1. R. R. Milburn, K. McRae, J. Chan, J. Tedrow, R. Larsen, M. Faul, *Tetrahedron Lett.* **2009**, *50*, 870–872.
2. K. M. Maloney, J. Y. L. Chung, *J. Org. Chem*. **2009**, *74*, 7574–7576.
3. G. M. Coppola, *Synthesis* **1988**, 81–84.
4. X.-M. Tao, W.-F. Li, X. Ma, X.-M. Li, W.-Z. Fan, L.-F. Zhu, X.-M. Xie, Z.-G. Zhang, *J. Org. Chem.* **2012**, *77*, 8401–8409.
5. S.-F. Zhou, D.-P. Li, K. Liu, J.-P. Zou, O. T. Asekun, *J. Org. Chem*. **2015**, *80*, 1214–1220.
6. T. Konno, T. Takehana, M. Mishima, T. Ishihara, *J. Org. Chem*. **2006**, *71*, 3545–3550.
7. S. Martinez-Erro, A. Sanz-Marco, A. B. Gómez, A. Vázquez-Romero, M. S. G. Ahlquist, B. Martín-Matute, *J. Am. Chem. Soc.* **2016**, *138*, 13408–13414.
8. A. Sanz-Marco, G. Blay, C. Vila and J. R. Pedro, *Org. Lett.* **2016**, *18*, 3538–3541.
9. H. Kawai, Z. Yuan, T. Kitayama, E. Tokunaga and N. Shibata, *Angew. Chem. Int. Ed.* **2013**, *52*, 5575–5579.
10. P. Kwiatowski, A. Cholewiak, A. Kasztelan, *Org. Lett*. **2014**, *16*, 5930–5933.
11. S.-X. Wu, D. Pan, C.-Y. Cao, Q. Wang, F.-X. Chen, *Adv. Synth. Catal.* **2013**, *355*, 1917–1923.
12. The turbo Grignard reagent was used. See, A. Krasovskiy, P. Knochel, *Angew. Chem. Int. Ed.* **2004**, *43*, 3333–3336.
13. V. Ya. Sosnovskii, I. S. Ovsyannikov, I. A. Aleksandrova, *Z. Organich. Khim.* **1992**, *28*, 518–526.
14. Y.-C. Yang, Y.-A. Shen, X.-L. Wang, Y. Zhang, D.-W. Wang, X.-D. Shi, *Tetrahedron Lett.* **2016**, *57*, 2280–2282.
15. M. Sugiura, Y. Ashikar, M. Nakajima, *J. Org. Chem.* **2015**, *80*, 8830–8835.
16. V. Bizet, X. Pannecoucke, J.-L. Renaud and D. Cahard, *Adv. Synth. Cat.* **2013**, *355*, 1394–1402.
17. D. Cahard, V. Bizet, X.-Y. Dai, S. Gaillard, J.-L. Renaud, *J. Fluorine Chem.* **2013**, *155*, 78–82.
18. H. Ueki, T. Chiba, T. Kitazume, *J. Org. Chem.* **2006**, *71*, 3506–3511.

19. V. Bizet, X. Pannecoucke, J.-L. Renaud, D. Cahard, *Angew. Chem. Int. Ed.* **2012**, *51*, 6467–6470.
20. X.-L. Xia, M. Wu, R.-H. Jin, T.-Y. Cheng, G.-H. Liu, *Green Chem.* **2015**, *17*, 3916–3922.
21. M. Wu, L.-Y. Kong, K.-W. Wang, R.-H. Jin, T.-Y. Cheng, G.-H. Liu, *Cat. Sci. Technol.* **2015**, *5*, 1750–1757.
22. H.-X. Zheng, Z.-F. Xiao, C. A.-Z. Yao, Q.-Q. Li, X.-S. Ning, Y.-B. Kang, Y. Tang, *Org. Lett.* **2015**, *17*, 6102–6105.
23. E. J. Corey, R. K. Bakshi, S. Shibata, *J. Am Chem. Soc.* **1987**, *109*, 5551–5553.
24 E. J. Corey, C. J. Helal, *Angew. Chem. Int. Ed*. **1998**, *37*, 1986–2012.
25. L. R. Jefferies, S. P. Cook, *Tetrahedron* **2014**, *70*, 4204–4207.
26. S. Okusu, Y. Sugita, E. Tokunaga, N. Shibata, *Beilstein J. Org. Chem.* **2013**, *9*, 2189–2193.
27. D. J. Wilger, N. J. Gesmundo and D. A. Nicewicz, *Chem. Sci.* **2013**, *4*, 3160–3165.
28. T. Konno, S. Yamada, A. Tani, M. Nishida, T. Miyabe, T. Ishihara, *J. Fluorine Chem.* **2009**, *130*, 913–921.
29. Y. Li, Z.-S. Ye, T. M. Bellman, T. Chi, M.-J. Dai, *Org. Lett.* **2015**, *17*, 2186–2189.
30. A. Morigaki, T. Miyabe, K. Tsukade, S. Arimitsu, T. Ishihara, T. Konno, *Synthesis* **2013**, *45*, 101–105.
31. A. Morigaki, T. Tanaka, T. Miyabe, T. Ishihara, T. Konno, *Org. Biomol. Chem.* **2013**, *11*, 586–595.
32. Gaussian 09W (Rev. D.01), M. J. Frisch, G. W. Trucks, H. B. Schlegel, G. E. Scuseria, M. A. Robb, J. R. Cheeseman, G. Scalmani, V. Barone, B. Mennucci, G. A. Petersson, H. Nakatsuji, M. Caricato, X. Li, H. P. Hratchian, A. F. Izmaylov, J. Bloino, G. Zheng, J. L. Sonnenberg, M. Hada, M. Ehara, K. Toyota, R. Fukuda, J. Hasegawa, M. Ishida, T. Nakajima, Y. Honda, O. Kitao, H. Nakai, T. Vreven, J. A. Montgomery, Jr., J. E. Peralta, F. Ogliaro, M. Bearpark, J. J. Heyd, E. Brothers, K. N. Kudin, V. N. Staroverov, R. Kobayashi, J. Normand, K. Raghavachari, A. Rendell, J. C. Burant, S. S. Iyengar, J. Tomasi, M. Cossi, N. Rega, N. J. Millam, M. Klene, J. E. Knox, J. B. Cross, V. Bakken, C. Adamo, J. Jaramillo, R. Gomperts, R. E. Stratmann, O. Yazyev, A. J. Austin, R. Cammi, C. Pomelli, J. W. Ochterski, R. L. Martin, K. Morokuma, V. G. Zakrzewski, G. A. Voth, P. Salvador, J. J. Dannenberg, S. Dapprich, A. D. Daniels, Ö. Farkas, J. B. Foresman, J. V. Ortiz, J. Cioslowski, D. J. Fox, Gaussian: Wallingford, CT, 2009.
